# Mitochondrial Unfolded Protein Response (mtUPR) Activation Improves Pathological Alterations in Cellular Models of Ethylmalonic Encephalopathy

**DOI:** 10.3390/antiox14060741

**Published:** 2025-06-16

**Authors:** José Manuel Romero-Domínguez, Paula Cilleros-Holgado, David Gómez-Fernández, Rocío Piñero-Pérez, Diana Reche-López, Ana Romero-González, Mónica Álvarez-Córdoba, Alejandra López-Cabrera, Marta Castro De Oliveira, Andrés Rodríguez-Sacristán, Susana González-Granero, José Manuel García-Verdugo, Angeles Aroca, José A. Sánchez-Alcázar

**Affiliations:** 1 Centro Andaluz de Biología del Desarrollo (CABD), Consejo Superior de Investigaciones Científicas (CSIC), Universidad Pablo de Olavide, 41013 Sevilla, Spain; jmromdom@upo.es (J.M.R.-D.); pcilhol@upo.es (P.C.-H.); dgomfer1@acu.upo.es (D.G.-F.); dreclop@alu.upo.es (D.R.-L.); aromgon1@upo.es (A.R.-G.); malvcor@upo.es (M.Á.-C.); alopcab2@alu.upo.es (A.L.-C.); 2 Neuropediatria, Neurolinkia, C. Jardín de la Isla, 8, Local 4 y 5, 41014 Sevilla, Spain; martadecastro@neurolinkia.com; 3 FEA Pediatría, Centro Universitario Hospitalar de Faro, R. Leão Penedo, 8000-386 Faro, Portugal; 4 Neuropediatría, Servicio de Pediatría, Hospital Universitario Virgen Macarena, 41009 Sevilla, Spain; arodriguezsacristan@us.es; 5 Departamento de Farmacología, Radiología y Pediatría de la Facultad de Medicina de la Universidad de Sevilla, 41009 Sevilla, Spain; 6 Laboratory of Comparative Neurobiology, Cavanilles Institute of Biodiversity and Evolutionary Biology, University of Valencia and CIBERNED-ISCIII, 46980 Valencia, Spain; susana.gonzalez@uv.es (S.G.-G.); j.manuel.garcia@uv.es (J.M.G.-V.); 7 Instituto de Bioquímica Vegetal y Fotossíntesis, Consejo Superior de Investigaciones Científicas, Universidad de Sevilla, 41092 Sevilla, Spain; aaroca@us.es

**Keywords:** ethylmalonic encephalopathy, ETHE1, mitochondrial diseases, SIRT3, mtUPR, bioenergetics, H_2_S, protein persulfidation

## Abstract

Ethylmalonic encephalopathy (EE) is a serious metabolic disorder that usually appears in early childhood development and the effects are seen primarily in the brain, gastrointestinal tract, and peripheral vessels. EE is caused by pathogenic variants in the gene that encodes the ETHE1 protein, and its main features are high levels of acidic compounds in body fluids and decreased activity of the mitochondrial complex IV, which limits energy production in tissues that require a large supply of energy. ETHE1 is a mitochondrial sulfur dioxygenase that plays the role of hydrogen sulfide (H_2_S) detoxification, and, when altered, it leads to the accumulation of this gaseous molecule due to its deficient elimination. In this article, we characterised the pathophysiology of ETHE1 deficiency in cellular models, fibroblasts, and induced neurons, derived from a patient with a homozygous pathogenic variant in *ETHE1*. Furthermore, we evaluated the effect of the activation of the mitochondrial unfolded protein response (mtUPR) on the mutant phenotype. Our results suggest that mutant fibroblasts have alterations in ETHE1 protein expression levels, associated with elevated levels of H_2_S and protein persulfidation, mitochondrial dysfunction, iron/lipofuscin accumulation, and oxidative stress. We also identified a cocktail of compounds consisting of pterostilbene, nicotinamide, riboflavin, thiamine, biotin, lipoic acid, and L-carnitine that improved the cellular and metabolic alterations. The positive effect of the cocktail was dependent on sirtuin 3 activation (SIRT3) and was also confirmed in induced neurons obtained by direct reprogramming. In conclusion, personalised precision medicine in EE using patient-derived cellular models can be an interesting approach for the screening and evaluation of potential therapies. In addition, the activation of the SIRT3 axe of mtUPR is a promising therapeutic strategy for rescuing *ETHE1* pathogenic variants.

## 1. Introduction

Ethylmalonic encephalopathy (EE) is an ultrarare and serious disease that usually develops in early childhood development. It has a progressive pattern and often ends in a fatal outcome. Genetically, this condition has an autosomal recessive inheritance and is determined by pathogenic variants in the gene encoding the ETHE1 protein [1]. Currently, less than 100 cases have been reported worldwide [2]. This pathology is usually diagnosed by a genetic analysis after the observation of certain typical biochemical markers and mainly affects tissues like the central nervous system, the gastrointestinal tract, and the peripheral blood vessels [3].

Clinically, this disorder often starts with symptoms such as developmental regression, hypotonia, petechiae, acrocyanosis, and chronic diarrhea. The neurological characteristics comprise spastic diplegia, ataxia, and developmental delay; however, as more patients with this condition are identified, the spectrum of this neurological phenotype continues to expand [4]. Brain abnormalities are common on magnetic resonances images [5].

From a biochemical and molecular point of view, this disease is characterised by the detection of high levels of acidic compounds in body fluids, both organic, such as ethylmalonic acid (EMA), methylsuccinic acid, and lactate, and inorganic, such as hydrogen sulfide (H_2_S), sulfite (SO_3_^2−^), and thiosulfate (S_2_O_3_^2−^); therefore, generalised acidosis is present [6,7]. Another characteristic abnormality of the disease is mitochondrial energy dysregulation associated with problems in proteins present in these organelles, which especially affects tissues that need a high energy supply, such as neuronal and muscular tissues [5].

The main cause of all metabolic alterations related to EE is the accumulation of H_2_S due to the impaired elimination of this molecule caused by pathogenic variants in the mitochondrial sulfur dioxygenase called Ethylmalonic Encephalopathy 1 (ETHE1), which is responsible for catalysing a key process in its main detoxification pathway [1,8]. This route starts when sulfide quinone oxidoreductase (SQOR) binds H_2_S and transfers it to glutathione (GSH), producing glutathione persulfide (GSSH). This GSSH is the substrate of ETHE1, which oxidises sulfane sulfur, producing sulfite and regenerating GSH. Sulfite is later oxidised to sulfate. If ETHE1 is dysfunctional, GSSH decomposes again into GSH and H_2_S, resulting in the accumulation of the latter [7,9].

Although H_2_S has various but poorly studied metabolic functions depending on cellular tissue, such as signaling in neurons or vascular endothelium, its excessive accumulation can lead to serious metabolic abnormalities and apoptotic processes [9].

Currently, the main therapeutic measures used to treat EE are mainly based on low-protein diets that restrict the amount of sulfur-containing amino acids and the use of compounds to alleviate the symptoms of this disease [10]. Some examples are the use of substances that improve mitochondrial performance, energy metabolism, and molecules with antioxidant properties such as coenzyme Q_10_, L-carnitine, and vitamin C, compounds that try to prevent the endogenous production of H_2_S such as N-acetylcysteine to “bypass” metabolic processes that lead to the production of H_2_S, antibiotics such as metronidazole that aim to reduce the amount of H_2_S produced by bacteria in the microbiota, and components that aim to neutralise the generalised acidosis that occurs in patients suffering from this disease, such as bicarbonate [11].

A novel method to combat this pathology that has recently gained strength is liver transplantation, since an important part of the detoxification of EMA, H_2_S, and other sulfured metabolites occurs in this organ; however, it is a method of addressing the disease that draws some controversy, since, although, in some cases, an improvement in symptoms is observed, in others, it is evident that the concentration values of EMA or C4/C6-acylcarnitines are not normalised, but are highly fluctuating in the time after the transplant, probably due to problems associated with the transplant itself. This technique only partially corrects the pathology and also carries the risks of the intervention itself, which are accentuated due to the young age of the patients [12,13].

In this article, we characterised the disease pathophysiology in cellular models, fibroblasts, and induced neurons (iNs), derived from a patient with a homozygous pathogenic variant in *ETHE1*. Moreover, we propose a potential pharmacological treatment that was able to significantly correct the mutant cellular phenotype.

## 2. Materials and Methods

### 2.1. Reagents

Anti-mitochondrially encoded Cytochrome C Oxidase Subunit II (mt-CO2) (ab79393), anti-NADH:Ubiquinone Oxidoreductase protein 8 (NDUFS8) (ab170936), anti-voltage dependent anion channel (VDAC) (ab14734), anti-Activating Transcription Factor 5 (ATF5) (ab184923), anti-sirtuin 1 (SIRT1) (ab110304), anti-nuclear respiratory factor 2 (Nrf2) (ab62352), anti-NFS1 cysteine desulfurase (NFS1) (ab58623), anti-LYR motif containing protein 4 (LYRM4) (ab253001), anti-frataxin (FXN) (ab219414), anti-NCOA4 (ab86707), anti-DMT1 (ab55735), anti-Mt-Ft (ab124889), anti-manganese superoxide dismutase (MnSOD) (ab68155), anti-peroxisome proliferator-activated receptor γ coactivator 1α (PGC1α) (ab191838), Goat anti-Rabbit IgG H&L (HRP) (ab6721), Rabbit anti-Mouse IgG H&L (HRP) (ab6728), Rabbit anti-Goat IgG H&L (HRP) (ab6741), Complex I (ab109720) Enzyme Activity Dipstick Assay Kit, Complex IV (ab109876) Enzyme Activity Dipstick Assay Kit, and L-Lactate detection kit (ab65330) were purchased from Abcam (Cambridge, UK).

Anti-β-actin (MBS448085) was purchased from MyBioSource (San Diego, CA, USA).

Anti-Ethylmalonic Encephalopathy 1 (ETHE1) (PA5-56040), anti-Cytochrome C Oxidase Subunit IV (COX-IV) (2A7B2), anti-heat shock protein 60 (HSP60) (MA3-012), anti-heat shock protein 70 (HSP70) (MA3-028), anti-sirtuin 3 (SIRT3) (PA5-13222), anti-TfR1 (13-6800), anti-iron sulfur cluster assembly scaffold protein (ISCU) (MA5-26595), anti-Mfrn2 (PA5-42498), anti- (GPX4) (MA5-32827), and nicotinamide (A15970.30) were purchased from Thermo Fisher (Waltham, MA, USA).

Anti-phosphorylated PGC1α (P-PGC1α) (AF6650) was purchased from R&D Systems (Minneapolis, MN, USA).

Anti-eukaryotic translation initiation factor 2α (eif2α) (9722S), anti-phospho-eif2α (9721S), anti-mitochondrially encoded NADH Dehydrogenase Subunit 1 (mt-ND1) (6888S), anti-mitochondrial transcription factor A (TFAM) (7495S), and anti-Activating Transcription Factor 4 (ATF4) (11815S) were purchased from Cell Signaling (Danvers, MA, USA).

Anti-Tau (sc-21796), anti-Cystathionine beta synthase (CBS) (sc-133154), anti-Cystathionine gamma lyase (CSE) (sc-374249), anti-Short-chain Acyl-CoA dehydrogenase (SCAD) (sc-365953), anti-dynamin related protein 1 (DRP1) (sc-32898), superoxide dismutase 1 (SOD1) (sc-101523), anti-ferritin light chain (FTL) (sc-74513), anti-Forkhead Box O3 (FOXO3A) (sc-48348), D-galactose (sc-202564), deferiprone (sc-211220), paraformaldehyde (PFA) (sc-253236B), oligomycin (sc-203342), antimycin A (sc-202467A), FCCP (sc-203578), DAPI (sc-3598), CPI-613 (sc-482709), thiamine (sc-205859), Biotin (sc-20476), and HEPES (sc-29097) were purchased from Santa Cruz Biotechnology (Dallas, TX, USA).

Anti-optic atrophy type 1 (OPA1) (HPA036926), Perl’s Prussian blue (03899), Sudan black, Luperox^®^ DI (tert-butyl peroxide) (168521), α-LA (62320), sodium hydrosulfide hydrate (NaHS H_2_O) (161527-100G), and donkey serum (D9663) were purchased from Sigma-Aldrich (Saint Louis, MO, USA).

Mitotracker^TM^ Deep Red FM (M22426), MitoSOX^TM^ Red (M36008), BODIPY^®^ 581/591 C11 (D3861), and ThiolTracker^TM^ Violet (T10096) were purchased from Invitrogen^TM^ Molecular Probes (Eugene, OR, USA). PBS (102309) was purchased from Intron Biotechnology (Seongnam, South Korea). Bovine Serum Albumin (BSA) (A6588.0100) was purchased from Applichem (Darmstadt, Germany). 3-TYP (HY-108331) was purchased from MedChemExpress (Sollentuna, Sweden).

### 2.2. Ethical Statements

The ethical committee of Hospital Universitario Virgen Macarena y Virgen del Rocío in Sevilla, Spain, granted approval in compliance with the Declaration of Helsinki principles, along with the International Conferences on Harmonisation and Good Clinical Practice Guidelines.

### 2.3. Patients and Fibroblast Culture

Fibroblasts were cultured from a skin biopsy obtained from a patient exhibiting a homozygous pathogenic variant in the *ETHE1* gene: c.488G>A (pArg163Gln). This variant has been detected in at least 9 patients reported in the literature with EE (https://www.ncbi.nlm.nih.gov/clinvar/variation/214322/, accessed on 5 May 2025). This variant is located at exon 4 where most of the pathogenic variants are concentrated (10/39, 25.6 %) [14]. Persulfide dioxygenase activity measured in recombinant human ETHE1 proteins (both wild-type and p.Arg163Gln) expressed in *E. coli* showed that the p.Arg163Gln recombinant *E. coli* only exhibited ~10% of wild-type catalytic activity, indicating that this variant impacts protein function [15]. This variant causes a combination of decreased protein stability and activity. Although structural analysis indicates that mutations do not change the protein’s folding, this variant exhibits increased susceptibility to proteolysis and reduced thermal stability [15].

A second patient was later included in the study harbouring a compound heterozygous pathogenic variant: c.488G>A (pArg163Gln) and c.221dup (p.Tyr74Ter).

Control fibroblasts were derived human skin primary fibroblasts from healthy volunteer donors. These control cells were sex- and age-matched. Patient and control samples were collected in compliance with the provisions set by the Helsinki Declarations of 1964, as updated in 2001. Fibroblasts derived from the patient and controls were maintained in culture conditions of 37 °C with 5% CO_2_, using DMEM (Dulbecco’s Modified Eagle Medium) fortified with 4.5 g/L glucose, L-glutamine, and pyruvate, alongside a 1% Pen-Strep antibiotic mixture (Thermo Fisher, Waltham, MA, USA) and 10–20% Fetal Bovine Serum (FBS) (Thermo Fisher, Waltham, MA, USA). Experiments were conducted using fibroblasts that had undergone fewer than eight passages.

### 2.4. Immunoblotting

Western blotting assays were performed using standard methods previously published by our group [16].

### 2.5. Quantification of Endogenous H_2_S

The determination of sulfide levels was carried out using ultra-performance liquid chromatography coupled with tandem mass spectrometry (UPLC-MS/MS), following prior methodologies and utilising monobromobimane for derivatisation [17].

Briefly, 70–80 mg of the frozen cells were homogenised in liquid nitrogen and metabolites were extracted in homogenous mixture of Tris-HCl buffer (100 mM, pH = 8.5; EDTA 1 mM), with shaking for 30 min at 4 °C. Samples were then sonicated for 5 min in ice-bath and centrifuged for 15 min, at 12,500× *g* at 4 °C; 100 μL of supernatants were derivatised with 25 μL of 15 mM monobromobimane (MBB) solution for 30 min at room temperature, and stopped by adding 25 μL of 5% formic acid. The mixture was subjected to centrifugation at 800× *g* for 10 min, and 1 μL of the supernatant was injected into the LC–MS/MS system for analysis. A calibration curve for NaHS concentrations was established ranging from 2.5 µM to 100 µM, and the H_2_S concentration in the samples was determined using this standard curve. The results are presented mean values ± SD of three different biological replicates.

### 2.6. Protein Persulfidation Assay: In-Gel Persulfide Detection in Cell Lysates

For in-gel persulfidation detection, a total of 100 mg of cell lysates was grounded in liquid nitrogen and total protein extract was prepared in 50 mM Tris-HCl, pH 8, supplemented with 1% protease inhibitor (cOmplete™, Sigma-Aldrich) and 2% SDS; then, it was incubated with 5 mM 4-chloro-7-nitrobenzofurazan (Cl-NBF) at 37 °C for 30 min, protected from light. A methanol/chloroform precipitation step was conducted to remove excess Cl-NBF, and the resulting protein pellets were rinsed with cold methanol, dried, and subsequently re-dissolved in 50 mM Tris-HCl pH 8 containing 2% SDS. Proteins were then incubated with 25 µM DAz-2:Cy-5 pre-click mix at 37 °C for 30 min (1 mM DAz-2 (Cayman Chemical, Ann Arbor, MI, USA), 1 mM Cyanine5 alkyne (Lumiprobe, Cockeysville, MD, USA), 2 mM copper(II)-TBTA complex (Lumiprobe), 4 mM ascorbic acid, 15 mM PBS, and 30% acetonitrile, mixed overnight at RT and quenched with 20 mM EDTA). After incubation, methanol/chloroform precipitation was conducted, and the resulting pellets were rinsed with methanol as previously detailed. The protein labelling was analysed by SDS-PAGE. Following SDS-PAGE, the gels underwent a 30 min fixation in a solution containing 12.5% methanol and 4% acetic acid, protected from light. The gel was imaged at 640 nm for Cy5 signal on an Ettan DIGE imager (GE Healthcare, Madrid, Spain). The same samples were examined by Western blot to check for equal loading using anti-β-actin to normalise the Cy5 signal. ImageJ software version 2.9.0 was utilised to quantify the signal from the protein immunoblot [18].

### 2.7. Immunofluorescence Microscopy

Fibroblasts, both treated and untreated, were cultured on glass coverslips of 1 mm width for a duration of 72 h using standard growth medium, with or without CoC3 supplementation. These cells were rinsed twice using PBS, fixed with 4% PFA for 15 min at ambient temperature, and subsequently treated with a blocking buffer (comprising 1% BSA in PBS) and permeabilised using 0.1% saponin within the blocking buffer for 1 h. Meanwhile, primary antibodies were prepared in a 1:100 dilution using antibody buffer (consisting of 0.5% BSA and 0.1% saponin in PBS). The fibroblasts were exposed to the primary antibodies overnight at 4 °C, and then washed twice with PBS. The secondary antibodies were proportionally diluted 1:400 in the antibody buffer, but their exposure time to the cells was shortened to 2 h at room temperature. The coverslips underwent another two rounds of washing with PBS and were marked with DAPI (at a concentration of 1 µg/mL in PBS) for 5 min, and then washed once more with PBS. Finally, they were mounted onto microscope slides with 10 µL of Mowiol.

Neurons were cultured on 4-well μ-SLIDE plates (Ibidi, Gräfelfing, Germany, 80426). The cells underwent three PBS washes before being fixed in 4% PFA for 10 min at room temperature, followed by permeabilisation with 0.1% Triton X-100 for 10 min. After that, a blocking buffer containing 5% donkey serum in PBS was applied for 1 h. Primary antibodies were diluted at a 1:100 ratio in PBS with 5% donkey serum and left on the cells overnight at 4 °C. The next morning, neurons were washed twice with PBS before adding the secondary antibodies, which were diluted 1:400 in PBS with 5% donkey serum and incubated for 2 h at room temperature. Finally, cells had two PBS washes, and were incubated for 10 min in PBS with 1 µg/mL DAPI, and washed three more times in PBS. Samples were viewed on a Leica mDMRE upright fluorescence microscope (Leica Microsystems GmbH, Wetzlar, Germany). Images were captured with a DeltaVision system (Applied Precision; Issaquah, WA, USA) using an Olympus IX-71 microscope with a 40× objective. The images were processed using softWoRx and Fiji-ImageJ version 2.9.0 software. The microscope conditions were kept consistent across all experiments.

### 2.8. Mitochondrial Complexes Activity

The activities of Complex I and IV were assessed following the guidelines provided by the Complex I (ab109720) and Complex IV (ab109876) Enzyme Activity Dipstick Assay Kits. Signal intensity measurements were obtained utilising the Chemidoc™ MP Imaging System and subsequently analysed with ImageLab™ software, version 6.1.

### 2.9. Mitochondrial Bioenergetics

Mitochondrial respiratory function of control and mutant fibroblasts was measured using Mitochondrial MitoStress test assay with an XF24 extracellular flux analyser (Seahorse Bioscience, Billerica, MA, USA, 102340-100) according to a previously published protocol [16]. The parameters studied were as follows: (1) basal respiration; (2) ATP production; (3) maximal respiration; and (4) spare respiratory capacity.

### 2.10. Determination of L-Lactate Levels

L-Lactate levels were evaluated using the L-Lactate detection kit (ab65330) according to the manufacturer’s protocol. The colour intensity was measured through a POLARstar Omega plate reader (BMGLabtech, Offenburg, Germany).

### 2.11. Analysis of Mitochondrial Network and Measurement of Mitochondrial Tubular/Rounded Ratio

The measurement of mitochondrial tubular/rounded ratio was conducted by using Mitotracker^TM^ DeepRed, a fluorescent dye insensitive to mitochondrial membrane potential. Both untreated and treated cells were cultured on 1 mm glass coverslips using DMEM glucose for three days. Following this period, the cells were incubated with 100 nM MitotrackerTM DeepRed at 37 °C for 45 min prior to fixation.

Image acquisition was performed utilising a DeltaVision system (Applied Precision; Issaqua, WA, USA) paired with an Olympus IX-71 fluorescent microscope equipped with a 40× objective. The intensity of cell fluorescence and the proportion of rounded versus tubular mitochondria were measured utilising Fiji-ImageJ software version 2.9.0. The rounded mitochondria were defined as 0.2–0.5 µm^2^ and tubular mitochondria as >0.5 µm^2^. The area of the mitochondrion was determined by taking into account both the major and minor axes of the organelle.

### 2.12. Measurement of Cell Membrane Lipid Peroxidation

Lipid peroxidation was evaluated using 4,4-difluoro-5-(4-phenyl- 1,3-butadienyl)-4-bora-3a,4a-diaza-s-indacene-3-undecanoic acid (BODIPY^®^ 581/591 C11) (Thermo Fisher), a lipophilic fluorescent dye [19,20]. Cells were incubated at 37 °C with 2 μM BODIPY^®^ 581/591 C11 for a duration of 30 min. As a positive control for lipid peroxidation, 500 μM Luperox^®^ was applied for 15 min. The assessment of lipid peroxidation in fibroblasts was conducted utilising the light and fluorescence Axio Vert A1 microscope equipped with a 20× objective lens. Image analysis was performed using Fiji-ImageJ software version 2.9.0.

### 2.13. Determination of Iron Accumulation

Iron deposition was assessed in fibroblasts and induced neurons through Perl’s Prussian blue (PPB) staining [21]. Images were acquired utilising a Zeiss Axio Vert A1 microscope, configured for both light and fluorescence microscopy, equipped with a 20× objective lens, and subsequently analysed via Fiji-ImageJ software, version 2.9.0.

Iron levels were additionally measured via inductively coupled plasma mass spectrometry (ICP-MS) employing an Agilent 7800 mass spectrometer (Agilent Technologies, Santa Clara, CA, USA). For ICP-MS analysis, cell extracts underwent acid digestion using nitric acid.

### 2.14. Determination of Lipofuscin Accumulation

Lipofuscin build-up was investigated through Sudan black staining in fibroblasts from both control and patient groups. Observations were performed using light and fluorescence microscopy on an Axio Vert A1 microscope (Zeiss, Oberkochen, Germany) equipped with a 20× objective, and the acquired images were analysed with Fiji-ImageJ software version 2.9.0.

### 2.15. Measurement of Mitochondrial Reactive Oxygen Species (ROS) Generation

Following the manufacturer’s guidelines, MitoSOX^TM^ Red was applied at a concentration of 5 μM to evaluate mitochondrial superoxide production in fibroblasts. Prior to this, cells were cultured on coverslips to 80% confluency. Confirmation of mitochondrial localisation for the MitoSOX^TM^ Red signal was achieved through MitoTracker^TM^ Deep Red FM staining, used at 100 nM for 45 min at 37 °C, which functions independently of mitochondrial membrane potential. The nuclei of the cells were counterstained with DAPI at 1 μg/mL. Post-staining imaging was performed using a DeltaVision system integrated with an Olympus IX-71 fluorescence microscope equipped with a 40× oil objective lens. The resulting images were processed using the Fiji-ImageJ software version 2.9.0.

### 2.16. TEM Analysis

Cells were seeded on 8-well Permanox chamber slides (Nunc, Thermo Fisher Scientific, Waltham, MA, USA). The cells underwent a triple wash using 0.1 M phosphate buffer (PB). Thereafter, the cells were fixed using a 3.5% glutaraldehyde solution, pre-equilibrated with 0.1 M PB, for either 5 min at 37 °C or 55 min at 4 °C. Post-fixation was performed with 2% OsO_4_ for 1 h at ambient temperature, followed by rinsing, dehydration, and embedding in Durcupan resin (Sigma-Aldrich, Saint Louis, MO, USA). Ultrathin sections of 70 nm thickness were subsequently cut using a diamond knife and analysed by a transmission electron microscope (FEI Tecnai G2 Spirit Bio-Twin, Thermofisher Scientific, Waltham, MA, USA) equipped with a 20-megapixel digital camera Xarosa, with images captured using Radius image acquisition software version 2.1 (EMSIS GmbH, Münster, Germany).

### 2.17. Direct Reprogramming

Neurons were generated from mutant and control fibroblasts by direct neuronal reprogramming following the methodology outlined by Drouin-Ouellet et al. [22,23]. Fibroblasts from control subjects and *ETHE1* mutant patients were seeded into μ-Slide 4-Well Ibidi plates (Ibidi) and maintained in DMEM Glutamax medium (61965059) supplemented with 1% Pen-Strep solution and 10% FBS.

On the following day, dermal fibroblasts were subjected to transduction using a singular lentiviral vector harbouring transcription factors specific to neural lineage (ASCL1 and BRN2), along with two shRNA targeting the REST complex. These elements were constructed as previously outlined, utilising an unregulated, universal phosphoglycerate kinase (PGK) promoter [24]. The plasmid was a gift from Dr. Malin Parmar (Developmental and Regenerative Neurobiology, Lund University, Lund, Sweden). The detailed protocol has been previously published [16]. At day 21 post-infection, cells were treated with CocT and the medium was changed every 2–3 days for 10 more days. Neuronal cells were recognised through Tau protein expression. The nuclei underwent staining using DAPI. Cells positive for both DAPI and Tau were classified as induced neurons (iNs). Conversion efficiency was determined by the ratio of Tau+ cells to the initial number of fibroblasts planted for conversion. Neuronal purity was assessed by calculating the proportion of Tau+ cells relative to the total cell count in the plate post-reprogramming.

### 2.18. Determination of Reduced Glutathione Levels

In order to evaluate reduced glutathione (GSH) levels, cells were cultured in DMEM glucose medium for a duration of 72 h. Following this period, the cells underwent two washes with 1× PBS and were then stained with 20 μM ThiolTracker^TM^ Violet for 30 min at 37 °C. Next, the cells were rinsed with 1× PBS and subsequently fixed with 4% PFA for a period of 10 min. Finally, image acquisition was carried out using a DeltaVision system equipped with an Olympus IX-71 fluorescence microscope (Applied Precision; Issaquah, WA, USA), and the images were analysed with Fiji-ImageJ software version 2.9.0.

### 2.19. Determination of the Labile Iron Pool (LIP)

To quantify LIP levels, cells were placed in 12-well plates. After a three-day period, cells were treated with HBSS containing 20 mM HEPES and 0.25 μM Calcein-AM at 37 °C for 15 min. Next, the cells were rinsed twice with HBSS and then incubated with HBSS plus 20 mM HEPES, 150 mM NaCl, and 5 mM glucose for another 10 min. Cells were subsequently exposed to 500 μM deferiprone for 10 min. Following deferiprone treatment, the initial fluorescence levels were recorded using a POLARstar Omega microplate reader (BMG Labtech, Offenburg, Germany). After an additional hour of incubation, a second fluorescence measurement was performed using the same reader. The LIP value was determined by calculating the ratio of the second fluorescence measurement to the initial one. Results were normalised to the protein content per sample.

### 2.20. Transfection with the Human ETHE1 Plasmid

For the cDNA complementation assays, we introduced human *ETHE1* cDNA tagged with FLAG (HG14681-CF, SinoBiological, Beijing, China) into cells from both control and EE patient groups. Initially, cells were placed in 6-well plates containing DMEM glucose medium with 10% FBS, without antibiotics, and allowed to grow for three days. Then, the cells underwent transfection with Lipofectamine^®^ 2000 (11668027, Thermo Fisher Scientific, Waltham, MA, USA) and FLAG-tagged human *ETHE1* cDNA in Opti-MEMTM I reduced serum medium (31985062, Thermo Fisher Scientific, Waltham, MA, USA) for a duration of 24 h. To detect the FLAG tag, the anti-DYKDDDDK tag antibody (A00187) was sourced from GenScript (Piscataway, NJ, USA).

### 2.21. Cell Survival Determination After Exposure to SIRT Inhibitors

The cell survival assay utilised a restrictive culture medium where galactose served as the primary carbon source. The goal was to eliminate glycolysis as an energy source by substituting glucose with galactose, thus forcing cells to depend solely on oxidative phosphorylation for ATP synthesis [25,26]. To additionally limit the survival of fibroblasts derived from patients, NaHS at a concentration of 100 µM was introduced to the galactose medium. Under these specific cell culture conditions, fibroblasts with the *ETHE1* mutation did not survive.

The galactose medium was formulated using DMEM that lacked glucose and glutamine, to which 10 mM galactose, 100 µM NaHS, 1% Pen-Strep antibiotic solution, and 10% FBS were added. Cells were seeded in 24-well plates in DMEM glucose. After 24 h, cells were treated for 72 h with CoC3. Next, medium was removed, cells were washed twice with PBS prior to the addition of the galactose with NaHS medium, and, with the purpose of inhibiting SIRT3 and SIRT1, SIRT inhibitors 3-TYP and EX-527 were added separately (T0). Then, the treatment was re-applied and images were taken in 24 h intervals for 72 h (T72). Cell counting and representative images were acquired using the BioTek^TM^ CytationTM 1 cell imaging multimode reader (Biotek, Winooski, VT, USA).

To ensure the specific inhibition of each SIRT with each compound (3-TYP to inhibit SIRT3 and EX-527 to inhibit SIRT1), the selected concentrations were 50 nM for 3-TYP, as this compound exhibits an IC50 (half maximum inhibitory concentration) of 16 nM for SIRT3 and 150 nM for EX-527, which is the IC50 of this compound for SIRT1 of 98 nM. The IC50 of 3-TYP for SIRT1 and SIRT2 are 88 nM and 92 nM, respectively, which requires a higher concentration of 3-TYP to inhibit these sirtuins. On the other hand, the IC50 of EX-527 for SIRT2 and SIRT3 is approximately 20 µM and 50 µM, respectively.

### 2.22. Quantitative Real-Time PCR (qPCR)

Gene expression of the *ETHE1* and *COX-IV* genes were evaluated through qPCR in both untreated and treated control and mutant fibroblasts. Total RNA was extracted using the RNeasy Mini Kit (74104, Qiagen, Venlo, The Netherlands). cDNA synthesis was performed using 1 µg of RNA with the iScript cDNA KIT (170-8891, BioRad, Hercules, CA, USA). Following this, qPCR was performed in accordance with the standard procedures and the SYBR Green Protocol. The *ETHE1* primers used were 5′-CTT CGT CCT GAA TGA CCA CAG C-3′ (FW) and 5′-CAG ACA GTC TCC TGG AAG TGT G-3′ (RV). The *COX-IV* primers used were 5′-GCA TGT CAA GCA CCT GTC TG-3′ (FW) and 5′-CAA CCG TCT TCC ACT CGT TC-3′ (RV). Actin served as a housekeeping control gene, with primers 5′-AGAGCTACGAGCTGCCTGAC-3′ (FW) and 3′-AGCACTGTGTTGGCGTACAG-5′ (RV). Primer selection was aided by the Primer3 online tool (accessed on 18 March 2025; https://primer3.ut.ee/).

### 2.23. Statistical Analysis

For this study, we employed nonparametric statistical methods, which do not rely on any assumptions regarding distribution, due to the questionable reliability of normality tests with the small sample sizes utilised. To assess the differences between groups, we applied the Mann–Whitney test for comparisons involving two groups and the Kruskal–Wallis test for multiple groups. The data are presented as mean ± SD from three independent experiments, with a *p*-value < 0.05 being considered statistically significant. All statistical analyses were conducted using GraphPad Prism 9.4.1 (GraphPad Software, San Diego, CA, USA).

## 3. Results

### 3.1. Pathological Variants of ETHE1 Cause Low Expression Levels of the ETHE1 Mutant Protein and Altered Sulfur Metabolism

**Figure 1:**
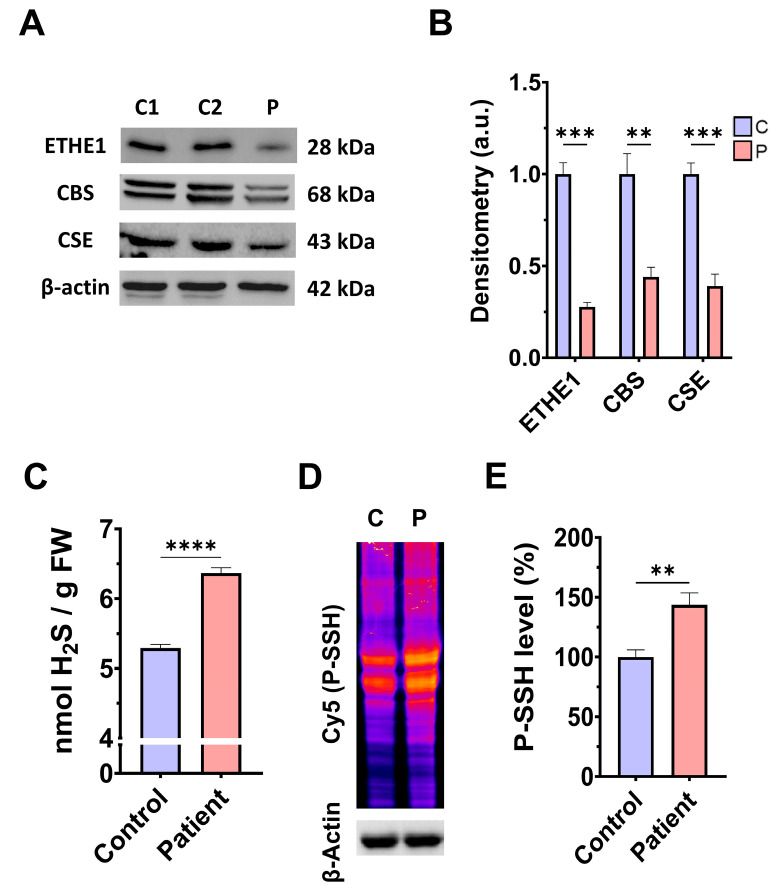
H_2_S metabolism and quantification and protein persulfidation levels. (**A**) Cellular extracts from the control (C1 and C2) and EE patient (P) were used to perform a Western blot analysis. SDS polyacrylamide gels were used to separate protein extracts and then the samples were immunostained using an antibody against ETHE1, CBS, CSE, and β-actin; the latter was used as a loading control. (**B**) Densitometry of Western blotting. The control samples (C1 and C2) were unified in one value (C) which represents the mean of the measurements of two control samples. Densitometry was referred to the control. (**C**) Cellular extracts from control (C) and fibroblasts derived from the EE patient (P) cells were used to quantify the amount of H_2_S by ultra-performance liquid chromatography–tandem mass spectrometry (UPLC-MS/MS). (**D**) Cellular extracts from control (C) and fibroblasts derived from the EE patient (P) cells were used to evaluate protein persulfidation levels by NBF-Cy5 in-gel detection assay. β-actin was used as loading control. (**E**) Quantification of fluorescence of the NBF/Cy5 in-gel detection assay which correlates to protein persulfidation. Data represent the mean ± SD of 3 independent experiments. ** *p* < 0.01, *** *p* < 0.001, and **** *p* < 0.0001 between control and patient fibroblasts. a.u.: arbitrary units.

First, we examined the pathological alterations of *ETHE1* pathogenic variants in EE-patient-derived fibroblasts. A Western blotting assay was performed to verify the expression levels of the mutant protein ETHE1, in addition to the expression levels of CBS and CSE, two of the three key enzymes that participate in the production of H_2_S. Fibroblasts derived from the EE patient showed reduced expression levels of the mutant ETHE1 protein, CBS, and CSE, with respect to control cells (Figure 1A,B). Low ETHE1 expression levels were confirmed by fluorescence microscopy (Figure S1A,B), and corresponded to decreased levels of ETH1 transcripts (Figure S1C). Next, we examined the intracellular H_2_S levels and total protein persulfidation in extracts of control and *ETHE1* mutant cells, two of the expected alterations of the disease. Recently, a growing body of evidence has shown that protein persulfidation, a post-translational modification of cysteine residues (R-SH) to persulfides (R-S-SH) elicited by H_2_S, is a fundamental mechanism of H_2_S-mediated signaling pathways [27]. Fibroblasts derived from the EE patient showed higher levels of H_2_S and protein persulfidation (Figure 1C–E).

### 3.2. Mitochondrial Function and Bioenergetics in Fibroblasts Derived from the EE Patient

**Figure 2:**
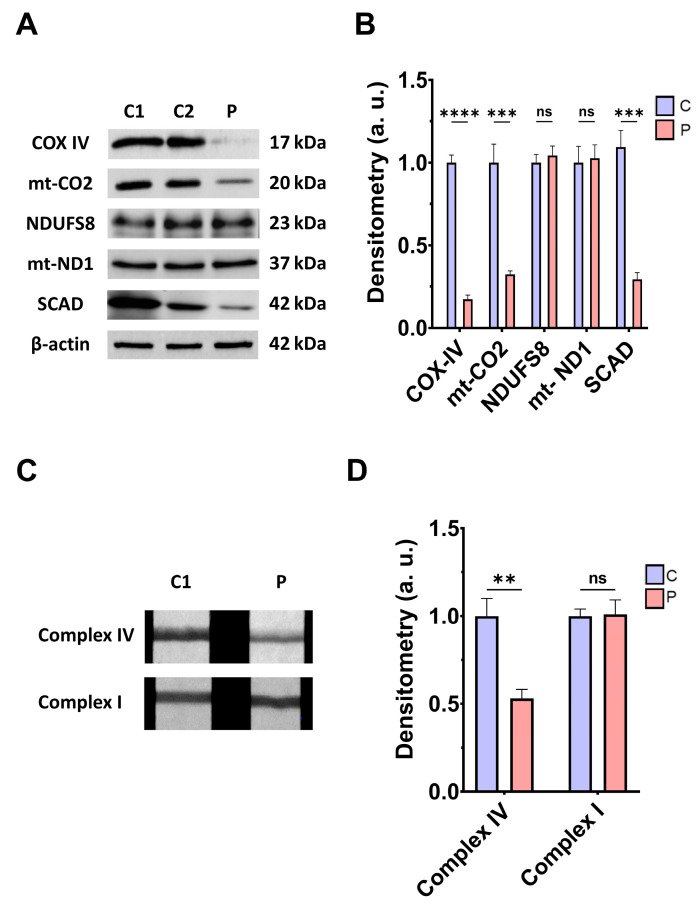
Mitochondrial function. (**A**) Protein expression levels of the COX-IV and mt-CO2 subunits of complex IV, NDUFS8, and mt-ND1 subunits of complex I, and SCAD. Cell extracts from control cells (C1 and C2) and fibroblasts derived from EE patient (P) cells were used to perform Western blot analysis. Samples were immunostained using antibodies against COX-IV, mt-CO2, NDUFS8, mt-ND1, SCAD, and β-actin; the latter was used as a loading control. (**B**) Densitometry of Western blotting. Control samples (C1 and C2) were unified in one value (C), which represents the mean of the measurements of the two control samples. Densitometry was referred to the control. (**C**) Complex IV and complex I activity through dipstick assays. (**D**) Densitometry of complex IV and complex I activity assays. Measurements were referred to the control. Data represent the mean ± SD of 3 independent experiments. ** *p* > 0.05, *** *p* < 0.001, and **** *p* < 0.0001 between control and patient fibroblasts; ns, non-significative. a.u.: arbitrary units.

Next, we addressed the consequences of *ETHE1* mutations on the mitochondrial proteins involved in bioenergetics, the activities of mitochondrial complex IV and complex I, L-lactate levels, and bioenergetic parameters. *ETHE1* mutations induced a marked reduction in the expression levels of the subunits of complex IV (subunits COX-IV and mt-CO2), without affecting the expression levels of the subunits of complex I (NDUFS8 and mt-ND1) (Figure 2A,B). Consequently, the activity of complex IV, but not complex I, was significantly reduced in fibroblasts derived from the EE patient compared to control cells (Figure 2C,D). Low expression levels of subunits IV of cytochrome c oxidase (COX-IV) were confirmed by fluorescence microscopy, and corresponded to decreased levels of COX-IV transcripts (Figure S2A–C). Moreover, expression levels of SCAD, a mitochondrial enzyme involved in fatty acid β-oxidation, specifically in the oxidation of short-chain acyl-CoA (C4–C6), which is known to be inhibited by high levels of H₂S, were also reduced in EE fibroblasts compared to control cells (Figure 2A,B).

**Figure 3:**
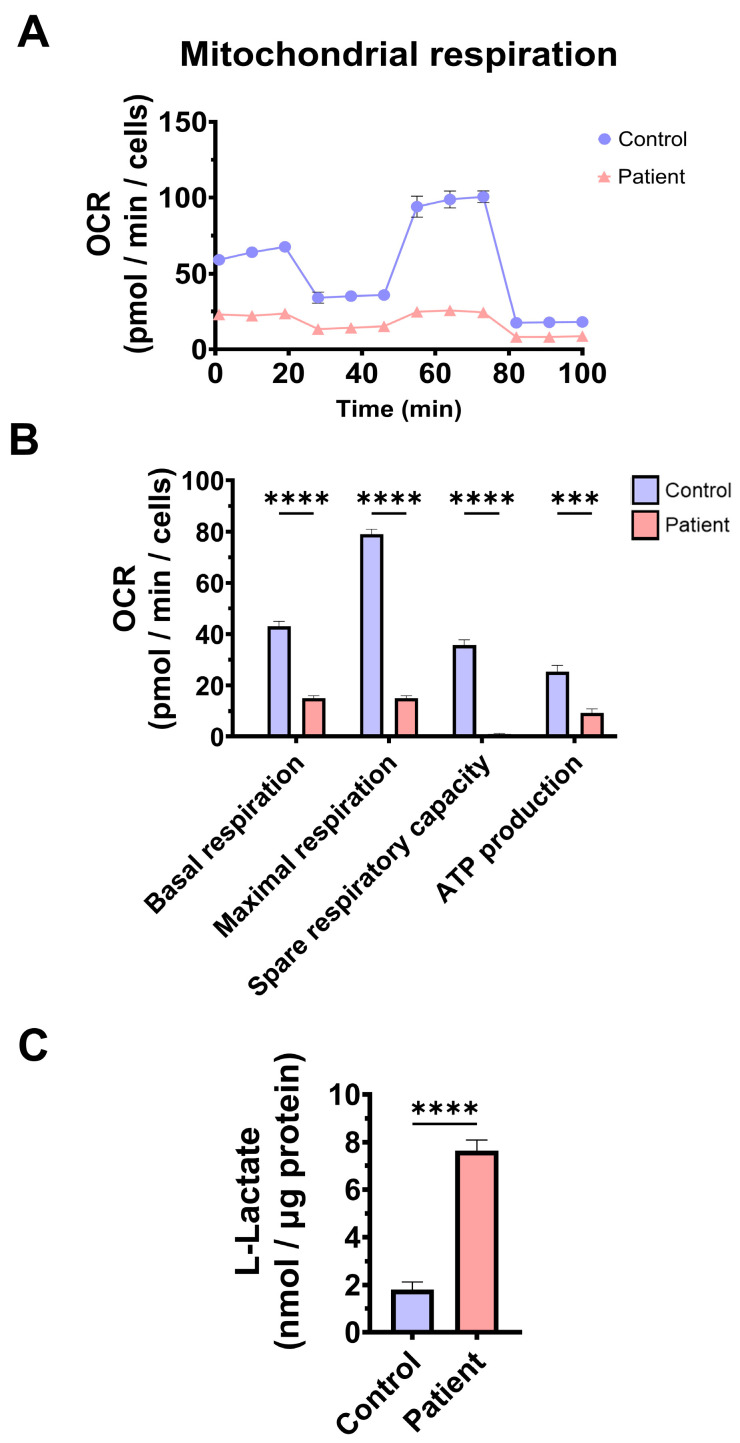
Mitochondrial bioenergetics of control and EE fibroblasts. (**A**) Mitostress bioenergetic assay in control and EE mutant cell lines. The mitochondrial respiration profile was measured with a Seahorse XFe24 analyser. (**B**) Basal and maximal respiration, spare respiratory capacity, and mitochondrial ATP production. (**C**) Quantification of the amount of L-lactate present in control (C) and EE fibroblasts (P) using a L-lactate detection assay kit. Nanomols of L-lactate were referred to micrograms of protein for normalisation. *** *p* < 0.001 and **** *p* < 0.0001 between the control and patient fibroblasts.

To confirm mitochondrial dysfunction in patient-derived fibroblasts, a Mitostress test assay was performed using the XF24 extracellular flux analyser (Seahorse Bioscience, Billerica, MA, USA, 102340-100). The results showed that maximal, basal, and spare respiration, and mitochondrial ATP production were significantly reduced in *ETHE1* mutant fibroblasts (Figure 3A,B). These alterations were associated with an increase in lactate levels in *ETHE1* mutant cells (Figure 3C). These data suggest marked mitochondrial dysfunction in ETHE1 fibroblasts.

### 3.3. Mitochondrial Mass and Dynamics in ETHE1 Fibroblasts

**Figure 4:**
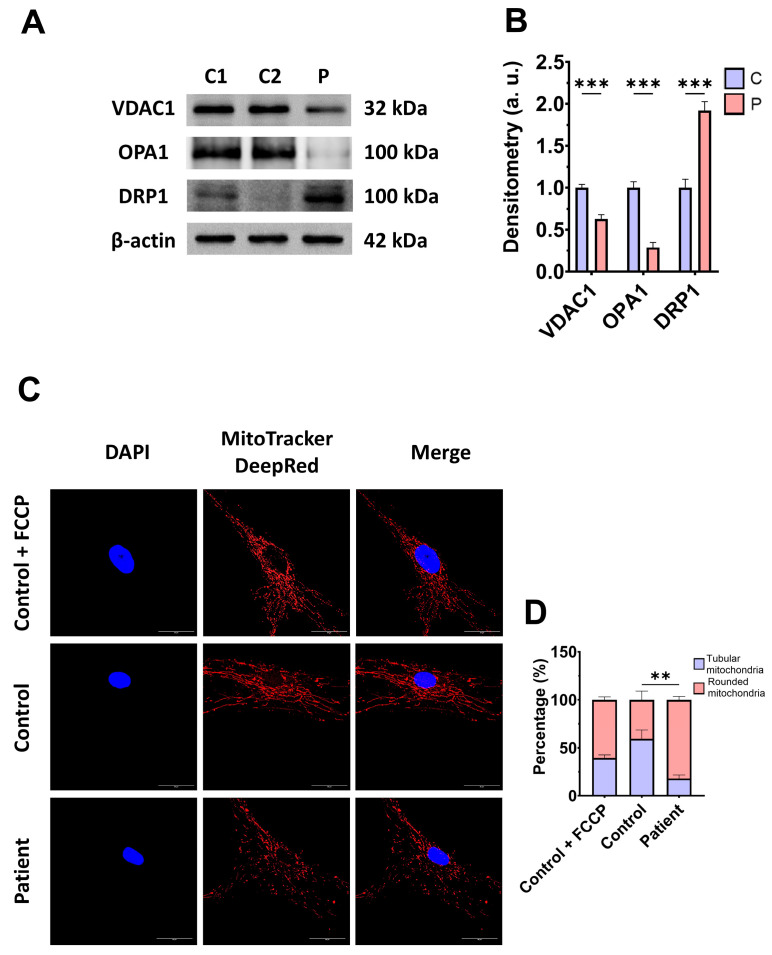
Expression levels of proteins related to mitochondrial mass and fusion/fission dynamics. (**A**) Cellular extracts from control (C1 and C2) and EE fibroblasts were analysed by Western blot. Membranes were immunostained using antibodies against VDAC1, OPA1, DRP1, and β-actin, the latter used as a loading control. (**B**) Densitometry of Western blotting. The control samples (C1 and C2) were unified in one value (C), which represents the mean of the measurements of the two control samples. (**C**) Representative images of the mitochondrial network using MitoTracker^TM^ DeepRed. Control cells treated with FCCP at 500 μm were used as a positive control of mitochondrial fragmentation. The cell nuclei were stained with DAPI. Scale bar: 50 μm. (**D**) Quantification of fluorescence intensity. Data were referred to the control and represent the mean ± SD of 3 independent experiments. ** *p* < 0.001, and *** *p* < 0.0001 between the control and patient fibroblasts. a.u.: arbitrary units.

Subsequently, we examine the mitochondrial mass by measuring the VDAC1 expression levels and mitochondrial morphology by MitoTracker^TM^ DeepRed staining, as markers of mitochondrial content and dynamics, in control and mutant fibroblasts. The expression levels of VDAC1 were decreased in EE fibroblasts with respect to the control cells, indicating a lower mitochondrial mass in mutant cells (Figure 4A,B). Additionally, *ETHE1* mutant fibroblasts showed a lower mitochondrial tubular/rounded ratio associated with mitochondrial fragmentation (Figure 4C,D). Consistent with these findings, the expression levels of the mitochondrial fusion protein OPA1 decreased while the expression levels of the fission protein DRP1 increased (Figure 4A,B).

### 3.4. The ETHE1 Mutation Causes Oxidative Stress and Lipid Peroxidation

**Figure 5:**
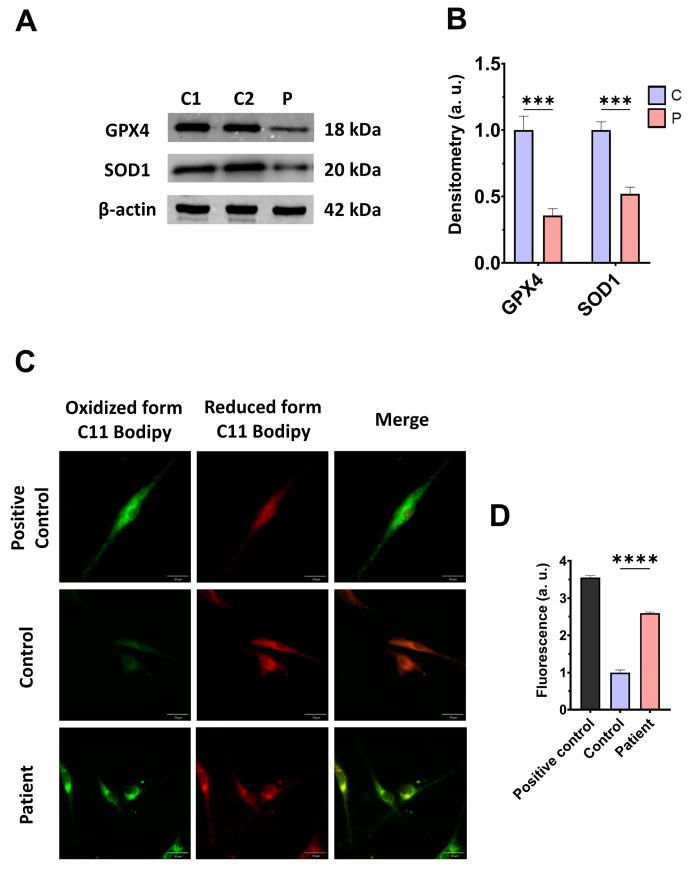
Oxidative stress and membrane lipid peroxidation in EE fibroblasts. (**A**) Cellular extracts from control (C1 and C2) and fibroblasts derived from the EE patient (P) fibroblasts were analysed by Western blot. Membranes were immunostained using antibodies against GPX4, SOD1, and β-actin, the latter used as a loading control. (**B**) Densitometry of Western blotting. The control samples (C1 and C2) were unified in one value (C), which represents the mean of the measurements of the two control samples. (**C**) Representative images of membrane lipid peroxidation with BODIPY 581/591 C11 staining. Control cells treated with Luperox at 500 μm were used as a positive control. Scale bar: 50 μm. (**D**) Quantification of the fluorescence intensity. Data were referred to the control and represent the mean ± SD of 3 independent experiments. *** *p* < 0.001 and **** *p* < 0.0001 between control and patient fibroblasts. a.u.: arbitrary units.

As expected, mitochondrial dysfunction in *ETHE1* mutant cells was associated with increased mitochondrial reactive oxygen species (ROS) production (Figure S3) and oxidative stress. For that reason, we analysed the expression levels of the antioxidant enzymes GPX4 and SOD1 to evaluate the protective mechanisms of cellular antioxidants. Both antioxidant enzymes were significantly decreased in *ETHE1* mutant cells (Figure 5A,B). Furthermore, since oxidative stress can cause cell membrane damage, the levels of cell membrane lipid peroxidation were examined. *ETHE1* mutant cells exhibited elevated lipid peroxidation in their cell membranes when contrasted with control fibroblasts (Figure 5C,D). These findings suggest that mutant cells are under oxidative stress and the protective antioxidant mechanisms are defective, leading to the lipid peroxidation of the cell membranes.

### 3.5. The ETHE1 Mutation Causes the Accumulation of Iron and Lipofuscin

**Figure 6:**
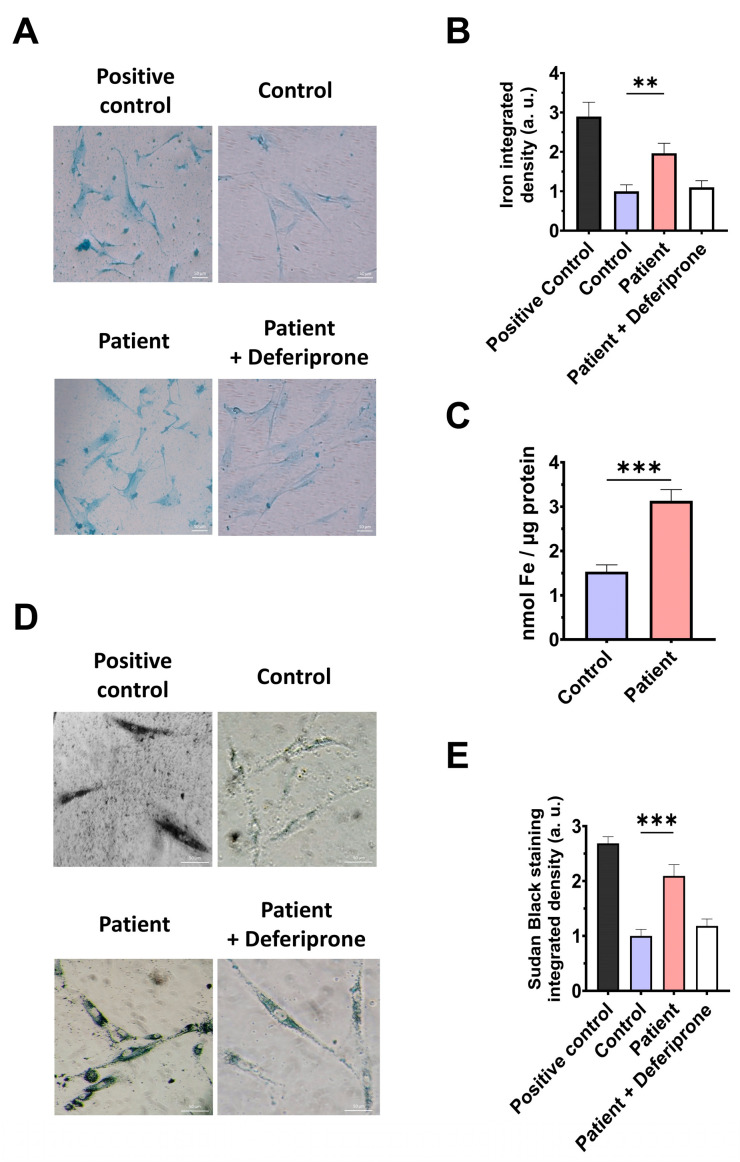
Iron and lipofuscin accumulation. (**A**) The fibroblasts from the control and the EE patient were subjected to Prussian blue staining. For the negative control, patient cells were treated with 100 µM Deferiprone. The images were taken using a Zeiss Axio Vert A1 microscope. As a positive control for iron accumulation, a PKAN (pantothenate-kinase-associated neurodegeneration) cell line was employed. Scale bar: 50 µm. (**B**) Quantification of Prussian-blue-staining-integrated density. The ImageJ software version 2.9.0 was utilised to analyse the images, with a minimum of 30 images examined for each condition and experiment. (**C**) Determination of iron content using ICP-MS. (**D**). Sudan black staining was used to evaluate the accumulation of lipofuscin. Patient cells treated with 100 µM Deferiprone were used as negative control. Images were acquired by a Zeiss Axio Vert A1 microscope. A PKAN (pantothenate-kinase-associated neurodegeneration) cell line was used as a positive control of lipofuscin accumulation [28]. Scale bar: 50 µm. (**E**). The quantification of Sudan-black-staining-integrated density (at least 30 images were analysed per each condition and experiment). Data represent the mean ± SD of 3 independent experiments. ** *p* < 0.01 and *** *p* < 0.001 between the control and patient fibroblasts. a.u.: arbitrary units.

However, as previous reports have highlighted the connection between mitochondrial dysfunction and iron metabolism dysregulation, we evaluated intracellular iron accumulation by Prussian blue staining. *ETHE1* mutant cells showed marked iron accumulation compared to control cells (Figure 6A,B). To verify the specificity of Prussian blue staining, fibroblasts from the EE patient underwent a 24 h treatment with 100 μM Deferiprone, an iron-chelating agent. Furthermore, iron overload in *ETHE1* mutant fibroblasts was confirmed by ICP-MS (Figure 6C). As iron can accumulate within lipofuscin granules, the accumulation of this pigment was analysed by Sudan black staining. To verify that the buildup of lipofuscin relies on iron, mutant cells were exposed to 100 μM Deferiprone for 24 h. The fibroblasts from the EE patient exhibited a notable increase in Sudan black staining relative to the control cells, suggesting an accumulation of lipofuscin (Figure 6D,E).

### 3.6. mtUPR Activation Increases the Expression Levels of the Mutant ETHE1 Protein and Normalises Sulfur Metabolism

**Figure 7:**
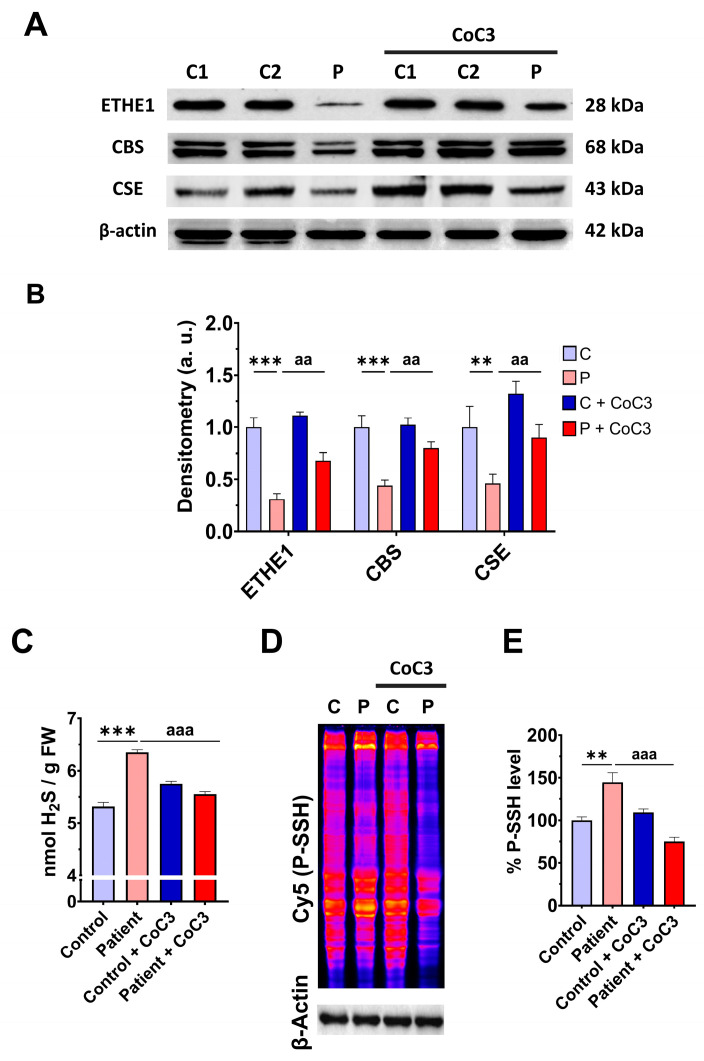
Effect of CoC3 treatment on H_2_S and protein persulfidation levels. (**A**) Cellular extracts from untreated and treated (for seven days with CoC3) control (C1 and C2) and EE fibroblasts (P) cells were analysed by Western blot. The membranes were immunostained using antibodies against ETHE1, CBS, CSE, and β-actin; the latter was used as a loading control. (**B**) Densitometric analysis of Western blots. The treated and untreated control samples were unified into one value for each condition (C and C + CoC3, respectively), representing the mean of each control experimental condition. Densitometry was referred to the untreated control value (C). (**C**) H_2_S levels were determined in cellular extracts by ultra-performance liquid chromatography–tandem mass spectrometry (UPLC-MS/MS). (**D**) Protein persulfidation levels were determined by the NBF-Cy5 in-gel detection assay. β-actin was used as loading control. (**E**) Quantification of fluorescence of NBF/Cy5 in-gel detection assay that correlates to protein persulfidation. Data represent the mean ± SD of 3 independent experiments. ** *p* < 0.01 and *** *p* < 0.001 between control and patient fibroblasts. ^aa^ *p* < 0.01 and ^aaa^ *p* < 0.001 between untreated and treated patient fibroblasts. a.u.: arbitrary units.

In previous work, we discovered a mitochondrial cocktail (CoC3) containing 1 μM Pterostilbene, 5 μM nicotinamide, 1 μM riboflavin, 1 μM thiamine, 1 μM biotin, 5 μM lipoic acid, and 1 μM L-carnitine that increases sirtuins’ activity and mtUPR activation [29]. CoC3 treatment for seven days improved pathological alterations in several mutant fibroblasts and induced neurons derived from mitochondrial patients. To examine the effect of CoC3 supplementation on patient-derived cells, *ETHE1* mutant fibroblasts were treated with CoC3 for one week, and the expression levels of the mutant protein ETHE1, CBS, and CSE, and H_2_S and protein persulfidation levels were evaluated. CoC3 treatment increased the levels of these proteins (Figure 7A,B) and, interestingly, reduced the levels of H_2_S (Figure 7C) and protein persulfidation (Figure 7D,E).

The upregulation of ETHE1 expression levels by CoC3 supplementation was also corroborated by fluorescence microscopy (Figure S4A,B) and assessing ETHE1 transcripts levels (Figure S4C). These data suggest that CoC3 upregulates the ETHE1 expression at both the transcriptional and protein levels, and may correct the main alterations in EE cell models.

### 3.7. Cocktail CoC3 Treatment Increases Cytochrome C Oxidase Activity and Mitochondrial Bioenergetics and Dynamics in ETHE1 Mutant Fibroblasts

**Figure 8:**
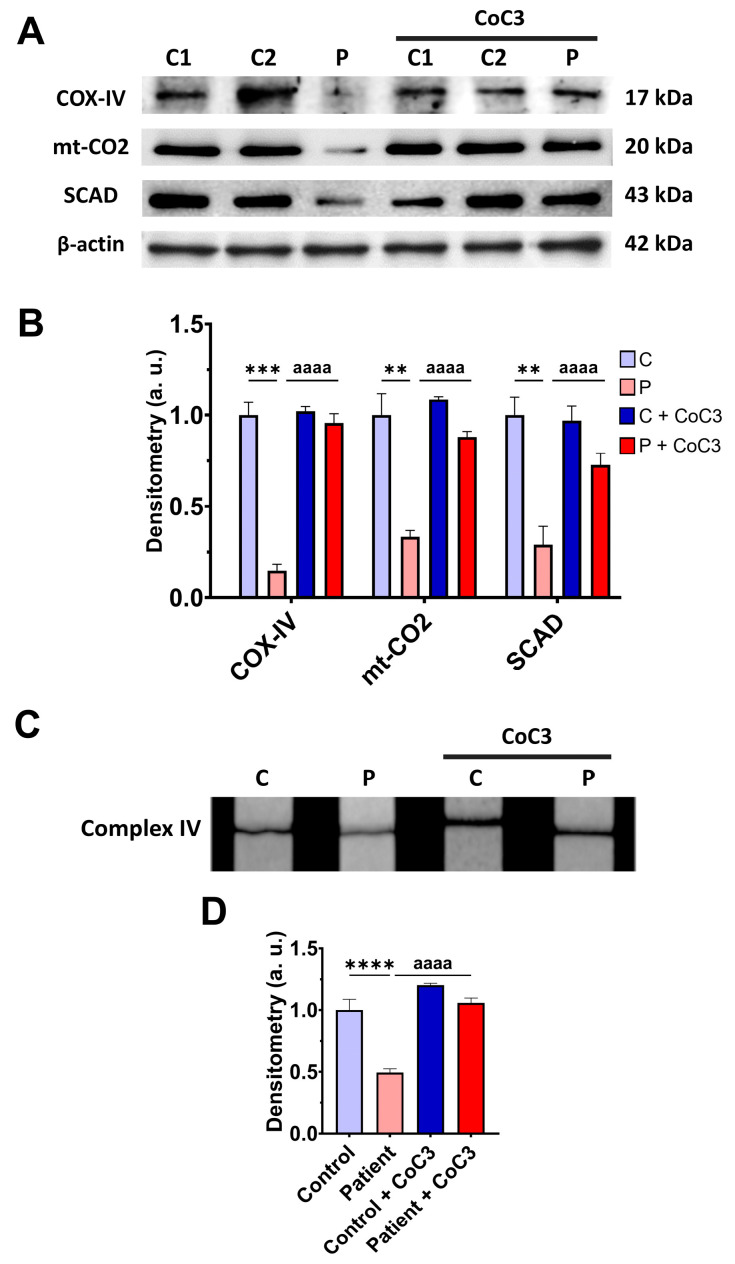
Effects of CoC3 treatment for seven days on mitochondrial function. (**A**) Cellular extracts from untreated and treated with CoC3 control (C1 and C2) and EE fibroblasts (P) cells were analysed by Western blot. The membranes were immunostained with antibodies against COX-IV, mt-CO2, SCAD, and β-actin; the latter was used as a loading control. (**B**) Densitometry of Western blotting. Untreated and treated control samples were unified in one value for each condition (C and C + CoC3, respectively), which represents the mean of each control measurement. Densitometry was referred to the untreated control value (C). (**C**) Complex IV activity by dipstick assay. (**D**) Densitometry of the complex IV activity assay. Measurements were referred to the control (C). Data represent the mean ± SD of 3 independent experiments. ** *p* > 0.05, *** *p* < 0.001, and **** *p* < 0.0001 between the control and patient’s fibroblasts. ^aaaa^ *p* < 0.0001 between the untreated and treated patient fibroblasts. a.u.: arbitrary units.

To address the beneficial effect of cocktail treatment on *ETHE1* mutant cells, we also examine the expression levels of the subunits of complex IV and SCAD, complex IV activity, mitochondrial bioenergetics, and L-lactate levels. CoC3 supplementation increased the expression levels of the COX-IV and mt-CO2 subunits of complex IV and SCAD (Figure 8A,B), and the activity of complex IV (Figure 8C,D).

The upregulation of the COX-IV subunit of complex IV expression levels by CoC3 supplementation was confirmed by fluorescence microscopy (Figure S5A,B) and assessing the COX-IV transcript levels (Figure S5C).

**Figure 9:**
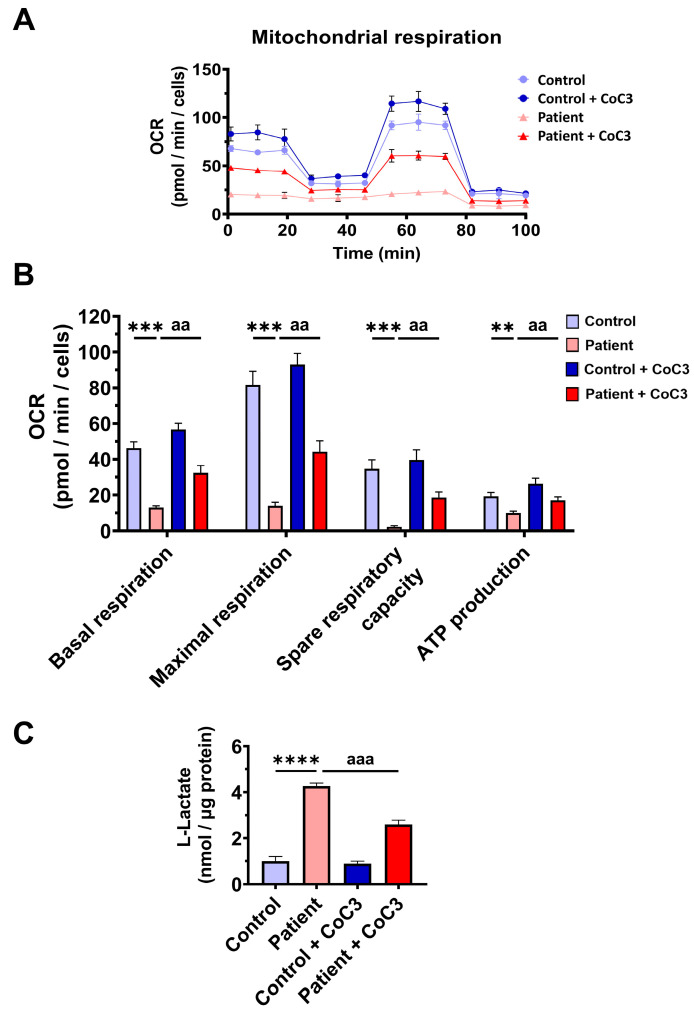
Effects of CoC3 treatment for seven days on mitochondrial bioenergetics. (**A**) Mitochondrial bioenergetics evaluated by the Seahorse analyser and normalised to 15,000 cells as described in Materials and Methods. (**B**) Values of basal and maximal respiration, spare respiratory capacity, and mitochondrial ATP production in control and patient fibroblasts. (**C**) Quantification of L-lactate levels in untreated and treated control (C) and patient (P) fibroblasts by an L-lactate detection assay kit. Nanomols of L-lactate were referred to micrograms of protein. ** *p* < 0.01, *** *p* < 0.001, and **** *p* < 0.0001 between the control and patient fibroblasts. ^aa^ *p* < 0.01 and ^aaa^ *p* < 0.001 between untreated and treated patient fibroblasts.

Likewise, CoC3 treatment restored basal, maximal, and spare respiration, as well as ATP production in *ETHE1* mutant cells (Figure 9A,B). As expected, CoC3 supplementation significantly decreased L-lactate levels (Figure 9C). These results suggest that CoC3 supplementation notably corrects the mitochondrial alterations in EE cellular models.

**Figure 10:**
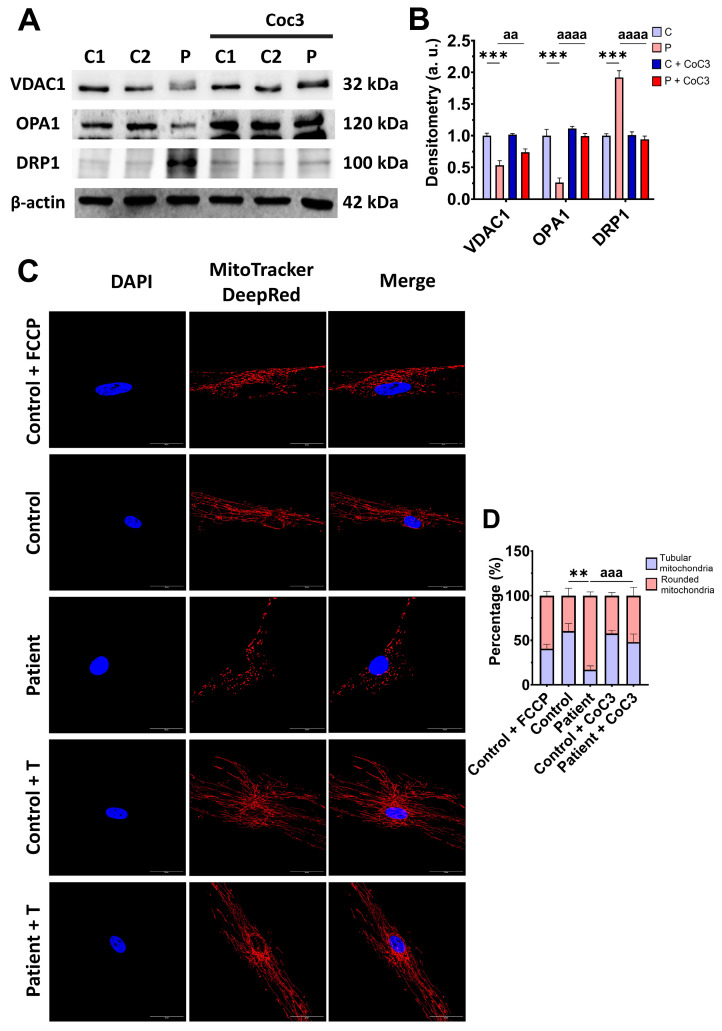
Effects of CoC3 treatment on protein expression levels related to mitochondrial mass and fusion/fission dynamics. (**A**) Cellular extracts from control (C1 and C2) and EE fibroblasts (P) fibroblasts were analysed by Western blot. The membranes were immunostained using antibodies against VDAC1 (serving as an indicator of mitochondrial mass), OPA1, DRP1, and β-actin, the latter used as a loading control. (**B**) Densitometry of Western blotting. The treated and untreated control samples were unified into one value for each condition (C and C + CoC3, respectively), representing the mean of each control experimental condition. Densitometry was referred to the untreated control value (C). (**C**) Representative images of the mitochondrial network using MitoTrackerTM DeepRed. Control cells treated with FCCP at 500 μm were used as a positive control of mitochondrial fragmentation. Cell nuclei were stained with DAPI. Scale bar: 50 μm. (**D**) Quantification of fluorescence intensity. Data were referred to the control and represent the mean ± SD of 3 independent experiments. ** *p* < 0.01 and *** *p* < 0.001 between the control and patient’s fibroblasts. ^aa^ *p* < 0.01, ^aaa^ *p* < 0.001, and ^aaaa^ *p* < 0.0001 between untreated and treated patient fibroblasts. a.u.: arbitrary units.

Confirming these results, CoC3 supplementation was able to restore the VDAC1, OPA1, and DRP1 expression levels (Figure 10A,B) and also correct the mitochondrial network and fragmentation (Figure 10C,D).

### 3.8. Cocktail CoC3 Treatment Improves Oxidative Stress and Lipid Peroxidation in EE Fibroblasts

**Figure 11:**
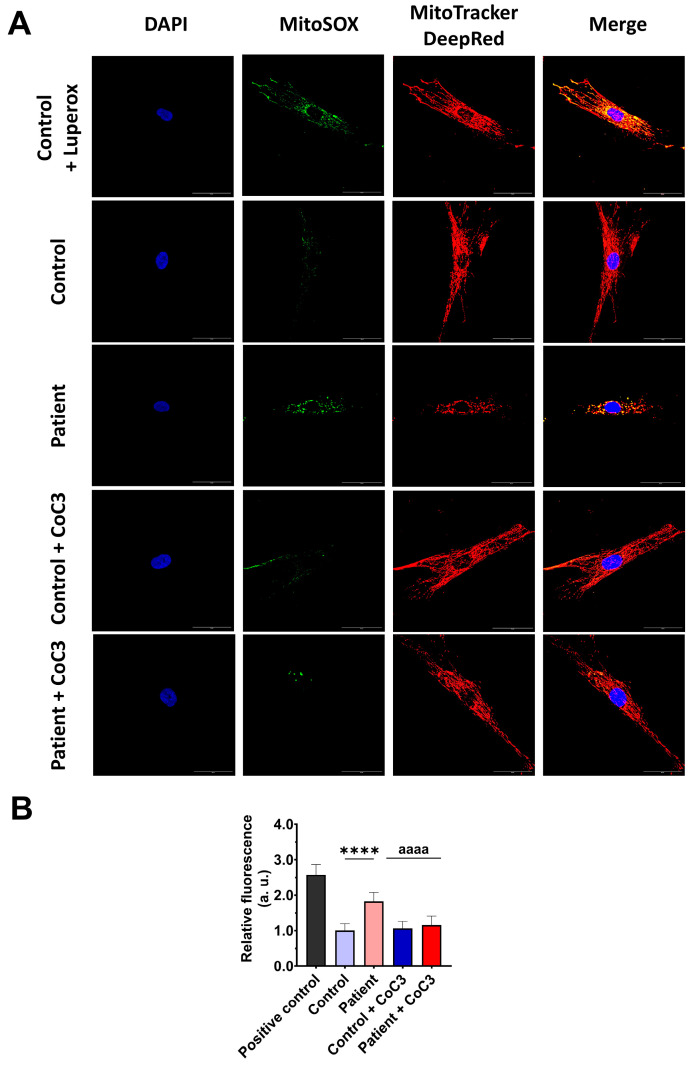
Effect of CoC3 treatment on mitochondrial superoxide anion levels. (**A**) Images illustrating control and EE fibroblasts, both untreated and treated with CoC3, stained using MitoSOX™ Red and MitoTracker™ DeepRed. DAPI staining was employed to reveal the nuclei. Images were taken under a wide-field fluorescence microscope using 40× lens and processed by ImageJ software. Scale bar = 50 µm. (**B**) Fluorescence quantification of the MitoSOXTM Red signal. Data were referred to the control and represent the mean ± SD of 3 separate experiments (at least 30 images for each condition and experiment were analysed). **** *p* < 0.0001 between the control and patient’s fibroblasts. ^aaaa^ *p* < 0.0001 between untreated and treated patient fibroblasts. a.u.: arbitrary units.

Next, we evaluated the effect of CoC3 supplementation on mitochondrial ROS, the expression levels of antioxidant enzymes, and lipid peroxidation. First, we corroborated that CoC3 treatment significantly decreased mitochondrial superoxide anion levels (Figure 11).

**Figure 12:**
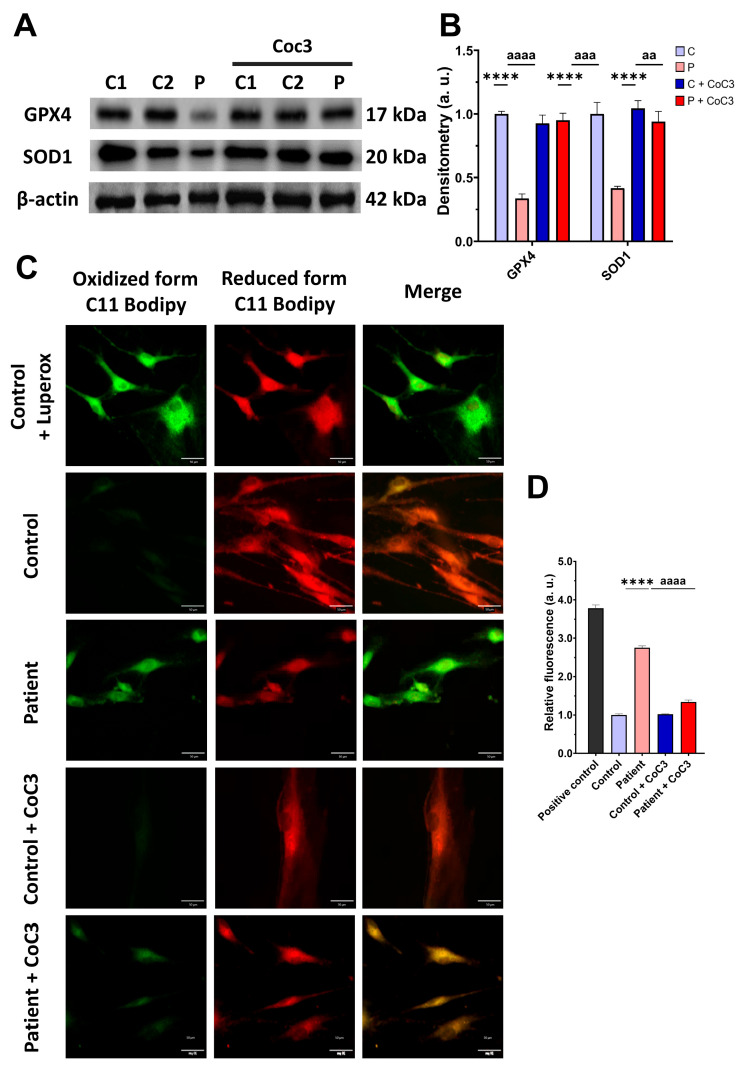
Effects of CoC3 treatment on antioxidant enzyme expression levels and lipid peroxidation. (**A**) Cellular extracts from untreated and treated with CoC3 control (C1 and C2) and EE (P) fibroblasts were analysed by Western blot. Antibodies targeting GPX4, SOD1, SOD2, and β-actin (utilised as a loading control) were used for immunostaining the samples. (**B**) Quantitative analysis of Western blotting. Untreated and treated control samples were unified in one value for each condition (C and C + CoC3, respectively), which represents the mean of each control measurement. Densitometry was referred to untreated control (C) value. (**C**) Representative images of membrane lipid peroxidation with BODIPY 581/591 C11 staining of untreated and treated with CoC3 control and EE patient fibroblasts. Control cells treated with Luperox at 500 μm were used as a positive control. Scale bar: 50 μm. (**D**) Quantification of the fluorescence intensity. Data were referred to the control and represent the mean ± SD of 3 independent experiments. **** *p* < 0.0001 between the control and patient fibroblasts. ^aa^ *p* < 0.01, ^aaa^ *p* < 0.001 and ^aaaa^ *p* < 0.0001 between untreated and treated patient fibroblasts. a.u.: arbitrary units.

Subsequently, we confirmed that the treatment increased the expression levels of the antioxidant enzymes GPX4 and SOD1 (Figure 12A,B) and decreased the membrane lipid peroxidation levels (Figure 12C,D) in EE fibroblasts.

Consistent with the beneficial effect of CoC3 supplementation on oxidative stress, the reduced glutathione levels were upregulated after CoC3 treatment (Figure S6).

### 3.9. Cocktail Treatment Prevents the Accumulation of Iron and Lipofuscin in ETHE1 Mutant Fibroblasts

**Figure 13:**
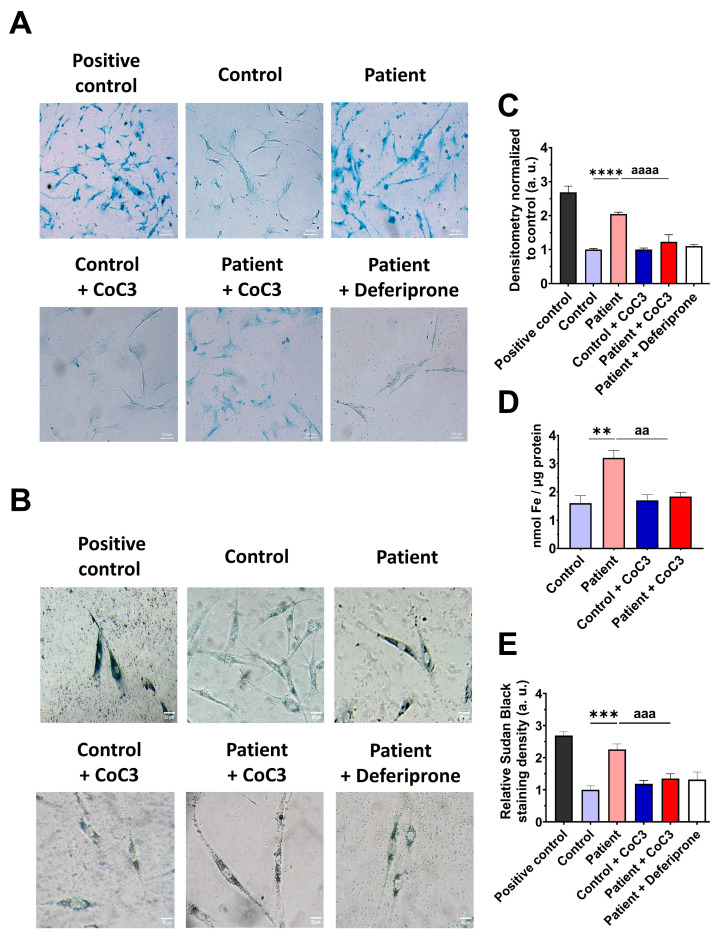
Effects of CoC3 treatment on iron and lipofuscin accumulation. (**A**) Untreated and treated control and EE patient’s fibroblasts were stained with Prussian blue staining. Patient’s cells treated with 100 µM Deferiprone were used as a negative control. A PKAN cell line (pantothenate-kinase-associated neurodegeneration) was used as a positive control of iron accumulation. Images were acquired by a Zeiss Axio Vert A1 microscope. Scale bar: 50 µm. (**B**) Measurement of the integrated density of Prussian blue staining. At least 30 images for each condition and experiment were examined using the ImageJ software version 2.9.0. Data were referred to the control and represent the mean ± SD of 3 independent experiments. (**C**) Quantification of iron content by ICP-MS of untreated and treated control and EE fibroblasts. (**D**) Lipofuscin accumulation was assessed by Sudan black staining. Mutant EE fibroblasts were treated with 100 µM Deferiprone as negative control. PKAN (pantothenate-kinase-associated neurodegeneration) fibroblasts served as a positive control for lipofuscin accumulation [28]. Images were acquired by a Zeiss Axio Vert A1 microscope. Scale bar: 50 µm. (**E**) Quantification of the density integrated with Sudan black staining (at least 30 images were analysed per condition and experiment). Data were referred to control and represent the mean ± SD of 3 independent experiments. ** *p* < 0.01, *** *p* < 0.001, and **** *p* < 0.0001 between control and patient fibroblasts. ^aa^ *p* < 0.01, ^aaa^ *p* < 0.001, and ^aaaa^ *p* < 0.0001 between untreated and treated patient fibroblasts. a.u.: arbitrary units.

Next, we examined the impact of CoC3 supplementation on iron overload in *ETHE1* mutant cells. Remarkably, following CoC3 treatment, mutant cells exhibited a significant decrease in iron accumulation, as assessed by Prussian blue staining and mass spectrometry (Figure 13A–C). In the same way, the accumulation of lipofuscin, addressed by Sudan black staining, was significantly reduced by CoC3 supplementation (Figure 13D,E). Moreover, to verify the specificity of Prussian blue staining for iron and the dependence of Sudan black staining on this trace element, mutant cells were exposed to 100 μM Deferiprone (Figure 13A,B,D,E).

Next, we evaluated the levels of the labile iron pool (LIP) in control and EE patient fibroblasts. The results showed that the higher levels of the LIP in *ETHE1* mutant cells were significantly reduced by CoC3 treatment (Figure S7).

In corroboration with the beneficial effect of CoC3 supplementation, the alterations in the expression levels of mitochondrial proteins involved in Fe-S cluster biogenesis, NFS1, LYRM4, ISCU, and FXN; proteins involved in iron storage, FTL, mt-FTL, and NCOA4; and iron transport proteins, TfR, DMT1, and Mfrn2, were also corrected (Figure S8).

**Figure 14:**
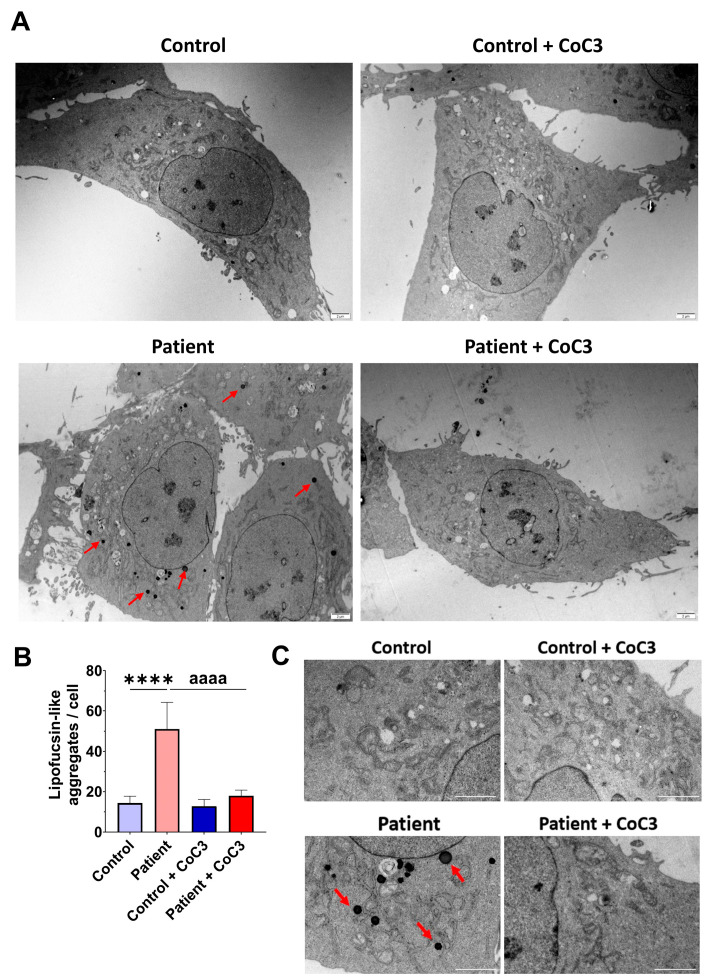
Electron microscopy images of untreated and treated control and EE patient’s fibroblasts. Cells were treated with CoC3 for seven days. (**A**) Illustrative examples of electron microscopy images. Scale bar: 2 µm. Red arrows highlight lipofuscin-like granules. (**B**) The measurement of lipofuscin-like aggregates per cell was conducted, analysing a minimum of 30 images for each condition and experiment. (**C**) Magnification of images of panel (**A**). Data represent the mean ± SD of 3 independent experiments. **** *p* < 0.0001 between control and patient fibroblasts. ^aaaa^ *p* < 0.0001 between untreated and treated patient fibroblasts.

To verify the lipofuscin presence in fibroblasts of the EE patient and assess the beneficial impact of CoC3 supplementation, electron microscopy was used to examine both control and *ETHE1* mutant cells. Compared to control fibroblasts, fibroblasts obtained from the EE patient exhibited an increased presence of intracellular lipofuscin-like granules. This accumulation was notably decreased following CoC3 supplementation (Figure 14).

Interestingly, the detailed examination of the TEM images in EE fibroblasts suggests that lipofuscin granules were formed within the mitochondria and released into the cytoplasm (Figure S9).

### 3.10. Functional Complementation with Wild-Type ETHE1 cDNA Reverts Pathophysiologic Characteristics in ETHE1 Mutant Fibroblasts

To verify ETHE1’s involvement in mitochondrial dysfunction and iron metabolism, we conducted cDNA complementation. In these assays, we introduced a FLAG-tagged human *ETHE1* cDNA into both control and *ETHE1* mutant fibroblasts. An immunofluorescence analysis using anti-ETHE1, anti-COX-IV, and anti-FLAG antibodies showed that mutant cells expressed low levels of ETHE1 and COX-IV, and no FLAG signal was detected. However, patient cells expressing recombinant ETHE1 (rETHE1) had a higher expression of ETHE1 (Figure S10) and COXIV (Figure S11) and the FLAG signal (Figures S10 and S11). Subsequently, we used Prussian blue staining to assess iron overload. The presence of recombinant ETHE1 notably reduced iron buildup in the mutant cells (Figure S12). Therefore, these data demonstrate a direct link between the expression of ETHE1, the downregulation of the subunits of complex IV, and iron overload.

### 3.11. CoC3 Cocktail Treatment Activates the Canonical and SIRT3 Transcriptional Axis of mtUPR, as Well as Mitochondrial Biogenesis in ETHE1 Fibroblasts

**Figure 15:**
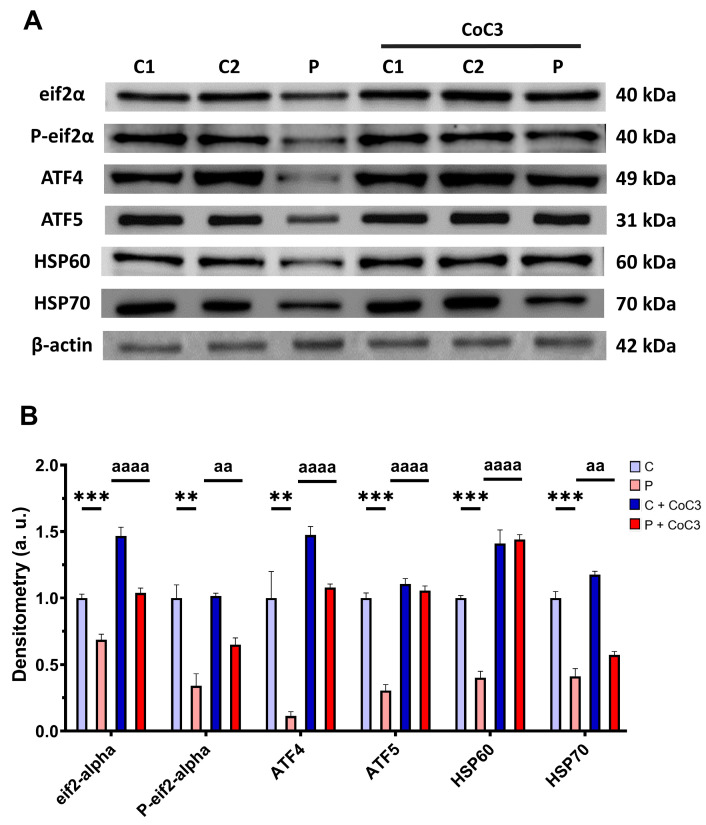
Effect of CoC3 treatment for seven days on the expression levels of proteins of the transcriptional canonical mtUPR axis. (**A**) Cellular extracts from untreated and treated with CoC3 control (C1 and C2) and fibroblasts derived from the EE patient (P) fibroblasts were analysed by Western blot analysis. The membranes were immunostained using antibodies against eif2α, P-eif2α, ATF4, ATF5, HSP60, HSP70, and β-actin, the latter used as a loading control. (**B**) Densitometry of Western blotting. Untreated and treated control samples were unified in one value for each condition (C and C + CoC3, respectively) which represents the mean of each control measurement. Densitometry was referred to the untreated control (C) value. Data were referred to the control and represent the mean ± SD of 3 independent experiments. ** *p* < 0.01 and *** *p* < 0.001 between control and patient fibroblasts. ^aa^ *p* < 0.01, and ^aaaa^ *p* < 0.0001 between untreated and treated patient fibroblasts. a.u.: arbitrary units.

Next, we assessed whether the positive impact of CoC3 was facilitated through the activation of mtUPR, a well-known mitochondrial compensatory mechanism [30,31]. We observed a reduction in the expression levels of proteins that participate in the transcriptional canonical axis of mtUPR eif2α, P-eif2α, ATF4, ATF5, HSP60, and HSP70 in the EE patient’s cells in comparison to control fibroblasts, which were significantly upregulated following CoC3 supplementation (Figure 15).

**Figure 16:**
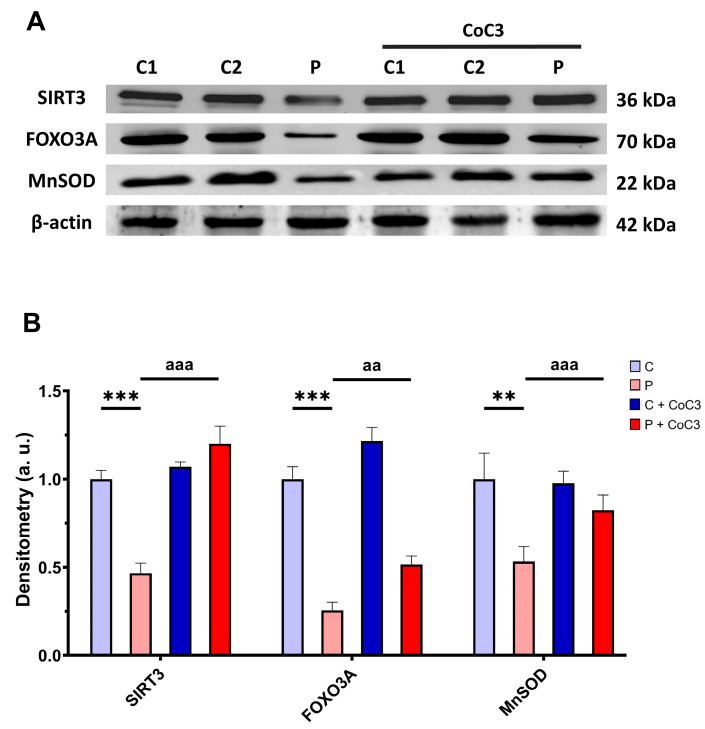
Effect of CoC3 administration on the expression levels of proteins of the SIRT3-mediated mtUPR pathway. (**A**) Cellular extracts from untreated and treated with CoC3 control (C1 and C2) and EE fibroblasts (P) were analysed by Western blot. Membranes were immunostained using antibodies against SIRT3, FOXO3A, MnSOD, and β-actin, the latter used as a loading control. (**B**) Densitometry of Western blotting. The untreated and treated control samples were unified into one value for each condition (C and C + CoC3, respectively) representing the mean of each control experimental condition. Densitometry was referred to the untreated control (C) value. Data were referred to control and represent the mean ± SD of 3 independent experiments. ** *p* < 0.01 and *** *p* < 0.001 between the control and patient fibroblasts. ^aa^ *p* < 0.01 and ^aaa^ *p* < 0.001 between untreated and treated patient fibroblasts. a.u.: arbitrary units.

Subsequently, we also analysed the expression levels of proteins involved in the SIRT3 axis of mtUPR SIRT3, FOXO3A, and MnSOD, and we found that the expression levels of these proteins were reduced in *ETHE1* mutant fibroblasts and CoC3 supplementation increased them (Figure 16).

**Figure 17:**
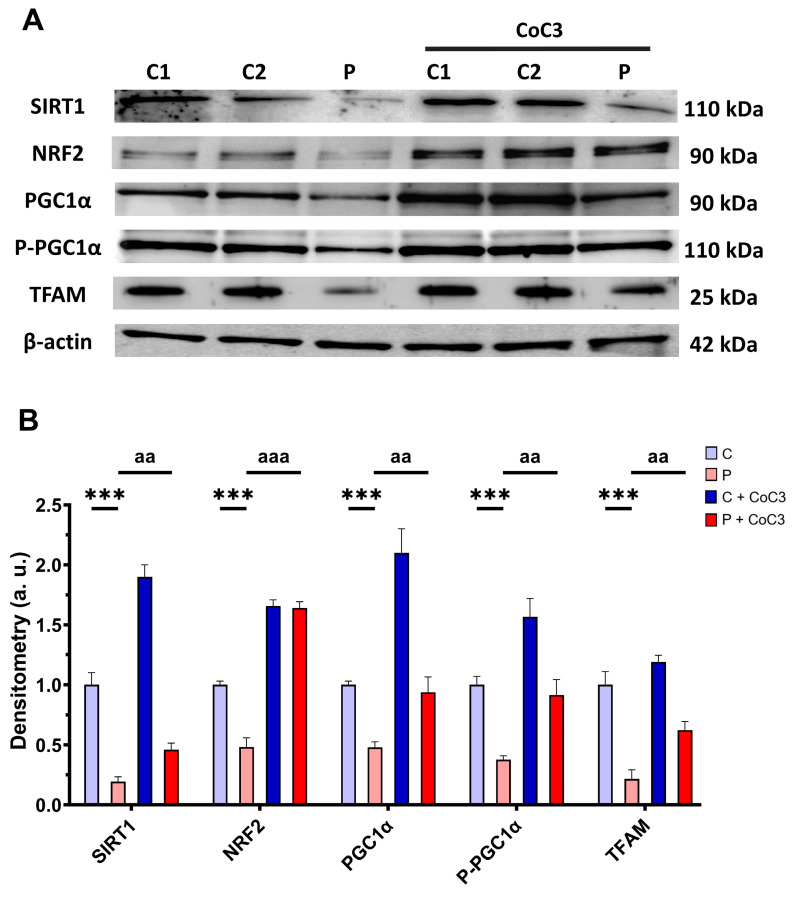
Effect of CoC3 treatment for seven days on the expression levels of proteins involved in SIRT1-mediated mitochondrial biogenesis. (**A**) Cellular extracts from untreated and treated with CoC3 control (C1 and C2) and EE fibroblasts (P) analysed by Western blot. Membranes were immunostained using antibodies against SIRT1, NRF2, PGC1α, P-PGC1α, TFAM, and β-actin, the latter used as a loading control. (**B**) Densitometry of Western blotting. Untreated and treated control samples were unified into one value for each condition (C and C + CoC3, respectively) which represent the mean of each control experimental condition. Densitometry was referred to the untreated control (C) value. Data were referred to the control and represent the mean ± SD of 3 independent experiments. *** *p* < 0.001 between the control and EE fibroblasts. ^aa^ *p* < 0.01 and ^aaa^ *p* < 0.001 between untreated and treated patient fibroblasts. a.u.: arbitrary units.

Furthermore, we evaluated the expression levels of proteins associated with SIRT1-mediated mitochondrial biogenesis, including SIRT1 itself, NRF2, PGC1α, P-PGC1α, and TFAM. We observed that the expression levels of the aforementioned proteins were downregulated in the EE patient’s fibroblasts and CoC3 treatment also elevated them (Figure 17).

**Figure 18:**
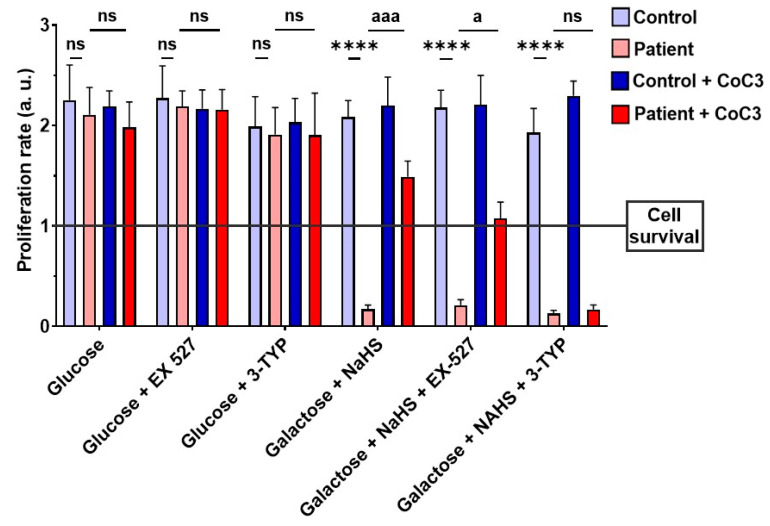
Proliferation ratio in stress medium of control and mutant cells under CoC3 supplementation in the presence of EX-527 or 3-TYP inhibitors. The proliferation ratio was determined as number of cells in T72 divided by number of cells in T0, in both the control and the mutant fibroblasts (values > 1: cell proliferation; values = 1: number of cells unchanged; values < 1: cell death). Data represent the mean ± SD of 3 independent experiments. EX-527 (150 nM): SIRT1 inhibitor, 3-TYP (50 nM): SIRT3 inhibitor. **** *p* < 0.0001 between control and patient fibroblasts. ^a^ *p* < 0.05, ^aaa^ *p* < 0.001 between untreated and treated patient fibroblasts; ns, non-significative. a.u.: arbitrary units.

Subsequently, to validate the impact of CoC3 on the activation of SIRT3 and SIRT1, 3-TYP was used as a specific inhibitor of SIRT3, while EX-527 served as an inhibitor of SIRT1. Therefore, we examined the effect of these inhibitors on the screening assay with a galactose medium with NaHS (stress medium). As expected, 3-TYP and EX-527 did not have negative consequences on control fibroblasts, and neither the glucose nor stress medium after 72 h (Figure 18, Figures S13, and S14). SIRT inhibitors did not affect the EE patient’s fibroblasts in the glucose medium either (Figure 18 and Figure S13). However, while *ETHE1* mutant fibroblasts were able to survive in the stress medium when treated with CoC3, the addition of 3-TYP (in the presence of CoC3) resulted in cell death (Figure 18 and Figure S14). On the other hand, in the presence of EX-527, although the proliferation rate of EE fibroblasts decreased, CoC3-treated cells were able to survive (Figure 18 and Figure S14).

Furthermore, 3-TYP prevented the increase in the expression levels of the ETHE1 protein and complex IV subunit COX-IV in *ETHE1* mutant cells under CoC3 treatment (Figure S15). These results imply that blocking SIRT3 prevents the beneficial impact of CoC3 supplementation. These results should be confirmed by the gene silencing of SIRT3 in future investigations.

### 3.12. Induced Neurons

Patient-derived fibroblasts, when utilised as cellular models, offered valuable insights into the disease’s pathophysiology. However, the most affected cell types in most metabolic mitochondrial disorders are those with high energy requirements, such as muscle cells and neurons [32,33]. Consequently, the direct conversion of fibroblasts obtained from patients into induced neurons (iNs) serves as a highly useful tool for investigating the pathogenesis of these disorders in general and EE in particular. Thus, both control and mutant fibroblasts underwent direct reprogramming into iNs. The reprogrammed cells exhibited a neuron-like shape and showed a positive immunoreactivity for Tau, a microtubule-associated protein typically present in the neuronal axons within the brains of vertebrates. In contrast, the unprogrammed cells lacked Tau staining.

Tau+ cells were employed to determine the efficiency of neuronal conversion, calculated as the proportion of Tau+ cells to the total number of seeded fibroblasts for conversion. This efficiency was approximately 32% for control cells (32.4% ± 3.8%) and 34% for *ETHE1* mutant cells (34.3% ± 5.1%). The neuronal purity (Tau+ cells relative to total cells in the plate after reprogramming) was approximately 71% (71.6% ± 8.1%) in control cells and up to 67% (67.7% ± 6.3%) in *ETHE1* mutant cells (Figure S15).

**Figure 19:**
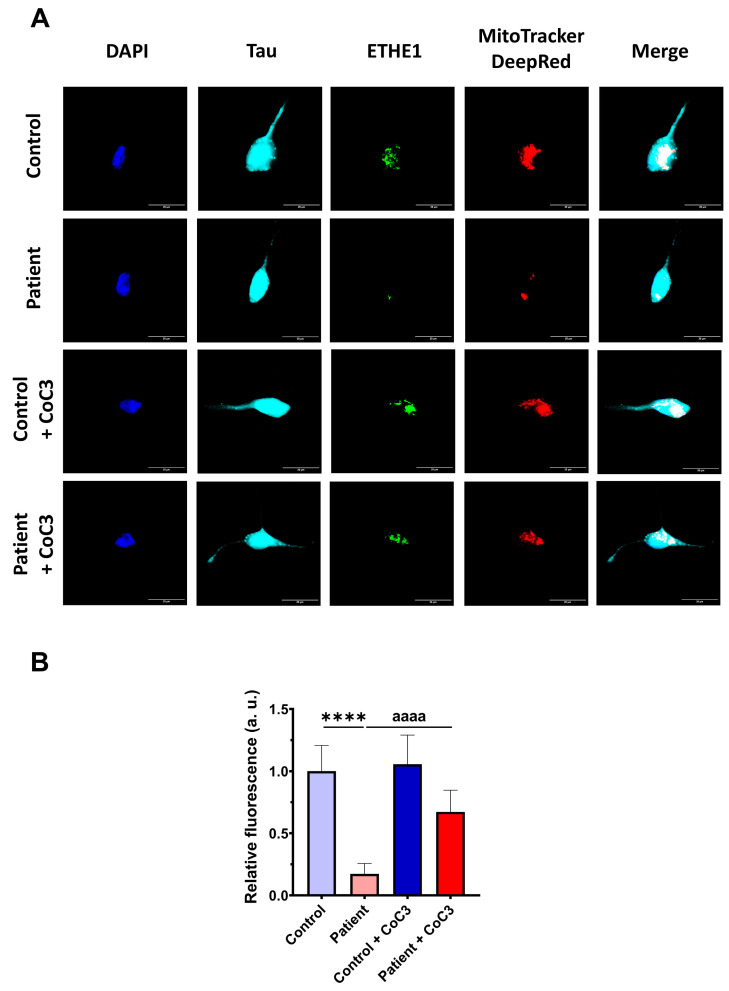
Effects of CoC3 treatment on ETHE1 expression levels in control and EE iNs. (**A**) iNs were fixed and immunostained with anti-ETHE1 antibodies. MitoTracker^TM^ DeepRed staining was employed to visualise mitochondria. The anti-Tau antibody signal was used as a neuronal marker. Nuclei were stained with DAPI. Scale bar: 50 μm. (**B**) Quantification of fluorescence intensity of ETHE1 antibody. Data were referred to control value. Images were analysed by Image J software version 2.9.0 (at least 30 images were analysed per each condition and experiment). **** *p* < 0.0001 between control and EE iNs. ^aaaa^ *p* < 0.0001 between untreated and treated patient iNs. a.u.: arbitrary units.

Then, the efficacy of CoC3 treatment was evaluated in control and *ETHE1* mutant iNs. The expression levels of ETHE1 and the complex IV subunit (COX-IV) were examined by an immunofluorescence assay. In mutant *ETHE1* iNs, the ETHE1 expression levels were almost completely absent (Figure 19). CoC3 supplementation partially reverted the ETHE1 levels on mutant *ETHE1* iNs as previously observed in fibroblasts (Figure 7 and Figure S4).

**Figure 20:**
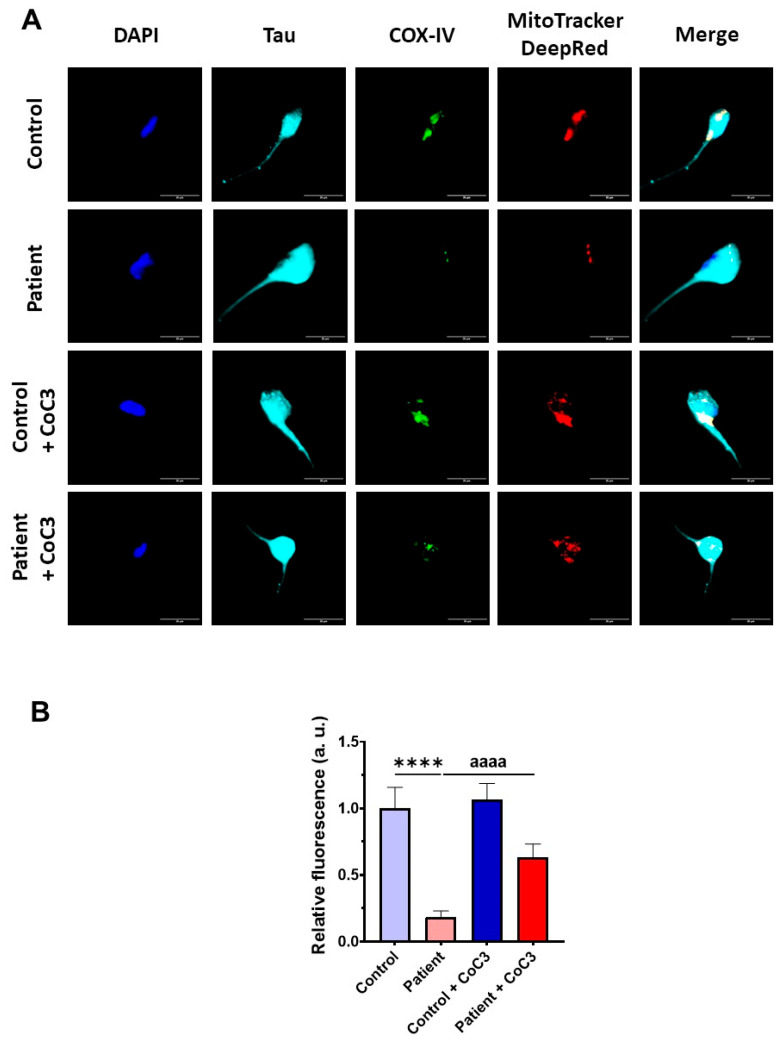
Effect of CoC3 treatment on COX-IV subunit expression levels in control and EE iNs. (**A**) iNs were fixed and immunostained with anti-COX-IV antibody. Mitochondria were visualised by MitoTracker^TM^ DeepRed staining. The Anti-Tau antibody signal was used as a neuronal marker. DAPI was used to stain the nuclei. Scale bar: 50 μm. (**B**) Quantification of the fluorescence intensity of the COX-IV antibody. Data were referred to control value. Images were analysed by Image J software version 2.9.0 (at least 30 images were analysed per each condition and experiment). **** *p* < 0.0001 between control and EE iNs. ^aaaa^ *p* < 0.0001 between untreated and treated patient iNs. a.u.: arbitrary units.

Furthermore, the COX-IV subunit expression levels were also almost completely absent and CoC3 supplementation partially reverted its levels in mutant *ETHE1* iNs (Figure 20) as previously observed in fibroblasts (Figure 8 and Figure S5).

**Figure 21:**
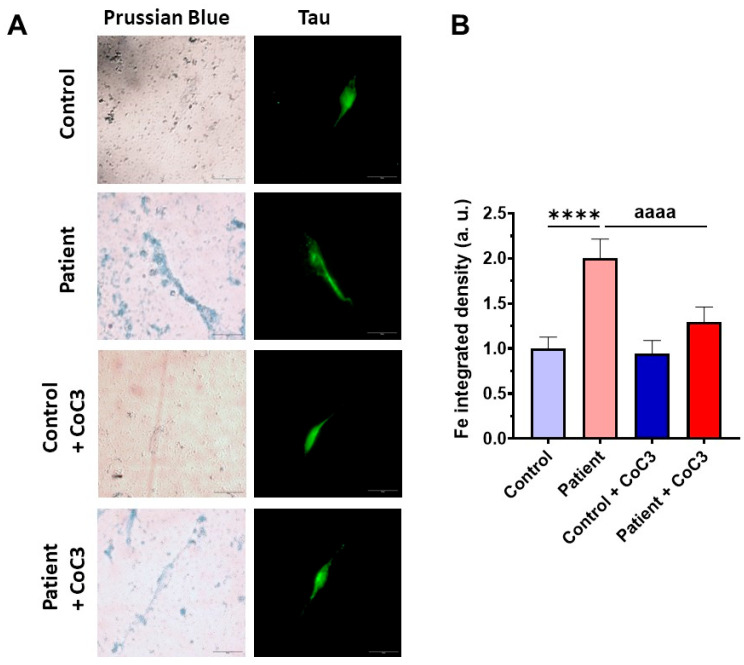
Effects of CoC3 treatment on iron overload in iNs generated derived from control and patient fibroblasts. (**A**) Representative images of Prussian blue staining were acquired with a Zeiss Axio Vert A1 microscope. Tau was used as a neuronal marker. Scale bar: 50 μm. (**B**) The quantification of Prussian blue staining was conducted using Image J software version 2.9.0, with a minimum of 30 images evaluated for each experimental condition. **** *p* < 0.0001 between control and patient iNs. ^aaaa^ *p* < 0.0001 between untreated and treated EE iNs. a.u.: arbitrary units.

To further investigate the physiopathological changes in iNs, we assessed the buildup of intracellular iron. Similar to the observations in fibroblasts, *ETHE1* mutant iNs exhibited iron overload; however, supplementation with CoC3 considerably mitigated iron accumulation, bringing it down to the levels observed in control iNs (Figure 21).

### 3.13. Effect of CoC3 Supplementations in Another Fibroblast Cell Line Derived from an EE Patient Recruited for This Work

During the completion of this work, a new patient was incorporated into the project. Fibroblasts derived from this patient (P2) showed similar physiological characteristics to the first patient (P1) and responded positively to CoC3 treatment as well (Figures S16–S19). Thus, both ETHE1 and COX-IV expression levels, mitochondrial bioenergetics, lipid peroxidation, and iron accumulation in P2 fibroblasts improved significantly after CoC3 supplementation.

## 4. Discussion

EE is a congenital disease that is still unknown in many aspects at the pathophysiological level and lacks a safe and effective treatment other than palliative therapies [6]. The symptoms are typically progressive and often lead to death in the first few years of life. Most patients have a severe phenotype with infantile onset, although a small portion manifests a milder clinical phenotype. To date, few cases classified as mild or atypical clinical phenotypes have been reported, showing a slight alteration in the metabolic profile and a slow neuromotor deterioration [2].

A common set of biochemical findings characterise EE: elevated levels of C4 and C5 plasma acylcarnitine species; the significantly increased urine excretion of ethylmalonic acid (EMA); elevated C4-6 acylglycines, particularly isobutyrylglycine and 2-methylbutyrylglycine; and persistent lactic acidemia [34]. Autozygosity mapping in a section of chromosome 19 revealed the location of the EE locus in 2004, and the gene was named *ETHE1* (Ethylmalonic Encephalopathy gene 1) [35].

In this work, the pathophysiology of EE has been studied using the patient’s own dermal fibroblasts as a biological model. The reasons are that the fibroblasts maintain the specific mutation that the patient possesses, and they can reproduce the main alterations that occur in the disease, such as increased H_2_S levels and mitochondrial dysfunction. Likewise, another advantage of working with dermal fibroblasts is that they are obtained by skin biopsies, which are less aggressive to patients than obtaining other cell types affected in this disease. In addition, fibroblasts can be direct reprogrammed to induced neurons, which are one of the most affected cell types in EE [36].

To investigate the pathological consequences of the mutation, we evaluated mitochondrial protein expression levels and mitochondrial function. Mutant cells exhibited a decreased expression of the mutant ETHE1 enzyme and complex IV subunits, linked to compromised mitochondrial function and iron accumulation, as well as increased oxidative stress and lipid peroxidation. Interestingly, supplementation with a cocktail, previously identified by our research group as a combination of activators of mtUPR, including pterostilbene, nicotinamide, riboflavin, thiamine, biotin, lipoic acid, and L-carnitine (CoC3), was able to correct the main pathological alterations [29]. This cocktail allowed *ETHE1* mutant cells to survive in the stress medium and significantly corrected the H_2_S levels and protein sulfidation, complex IV activity and, consequently, mitochondrial function, iron overload, and lipid peroxidation, among other metabolic defects.

### 4.1. Consequences of H_2_S Accumulation

ETHE1 is an iron-containing protein from the metallo β-lactamase family involved in the oxidation of mitochondrial sulfide to sulphate in the mitochondrial matrix [1]. Mutations in *ETHE1* that cause a loss of function result in sulfide toxicity and fatal disease [15,34]. H_2_S is a water-soluble molecule, gaseous at ambient pressure and temperature, which functions as a signaling molecule, but is toxic at high concentrations [7,37], similar to several gasotransmitters such as CO and NO [38]. In healthy humans, low concentrations of H_2_S (<25 μM) perform a variety of physiological tasks such as neuronal signaling, cardioprotection, heart rate regulation, vasorelaxation, angiogenesis, and epithelium-specific antioxidant, anti-inflammatory, and cytoprotective effects [34]. Cysteine β-synthase (CBS), cystathionine γ-lyase (CSE), and 3-mercaptopyruvate sulfotransferase (3-MST) synthesise H_2_S and play key roles in cysteine synthesis and metabolism. Furthermore, intestinal bacteria also produce a significant amount of H_2_S. However, at high concentrations, H_2_S is a potent toxin that inhibits several significant enzymes with antioxidant and energy-producing properties. Short-chain acyl CoA dehydrogenase (SCAD), carbonic anhydrase, and cytochrome c oxidase (mitochondrial electron transport chain complex IV) are the primary enzymes whose impairment results in clinical and laboratory findings during H_2_S intoxication [39].

The main pathomechanisms involved in EE are as follows [6]: (1) Bioenergetics dysfunction: Hydrogen sulfide (H_2_S), ethylmalonic acid (EMA), and thiosulfate accumulation provoke respiratory chain, tricarboxylic acid cycle, and creatine kinase inhibition, resulting in ATP depletion and increased lactate levels; (2) Increased protein persulfidation (P-SSH) by H_2_S; (3) Oxidative stress: H_2_S and EMA induce the generation of reactive species (RS) and oxidative damage to lipids and proteins, and decrease reduced glutathione (GSH) levels; (4) Mitochondrial permeability transition (mPT) induction: H_2_S and EMA induce mPT pore opening; (5) Mitochondrial dynamics alterations: levels of proteins involved in fusion (mitofusins 1 and 2, MFN1 and 2, and optic atrophy 1, OPA1) and fission (dynamin-related protein 1, DRP1) are altered; (6) Endoplasmic reticulum (ER)–mitochondria crosstalk disturbances: levels of proteins involved in communication between these organelles (inositol 1,4,5-triphosphate receptor—IP3R, and voltage-dependent anion-selective channel protein 1, VDAC1) are altered; (7) ER stress: levels of DNA damage inducible transcript 3 (DDIT3) increase; and (8) Increased apoptosis.

### 4.2. Protein Persulfidation

Among all these pathomechanisms, protein persulfidation may be of special interest because this post-translational modification has become increasingly recognised as the main mechanism by which H_2_S controls cellular functions [40]. Within biological systems, H_2_S exhibits a reactivity that can be categorised into three groups: (i) interacting with or neutralising reactive oxygen and reactive nitrogen species (ROS and RNS); (ii) binding to and/or engaging in subsequent redox reactions with metal centers; and (iii) reaction with proteins, herein called persulfidation [41]. Thus, Snyder’s group proposed that persulfidation, an oxidative post-translational modification of protein cysteine residues (P-SH), is the main mechanism for H_2_S signaling by regulating the protein structure and function [42,43,44].

Recent developments in persulfide labelling techniques have begun to unravel the role of this modification in pathophysiology mechanisms. Protein persulfuration levels are important for cellular defence against oxidative damage, although they decrease with ageing, leaving proteins vulnerable to oxidative damage [40]. Furthermore, ageing is one of the main risk factors for many neurodegenerative diseases and persulfidation has been shown to be dysregulated in Parkinson’s, Alzheimer’s, Huntington’s disease, and Spinocerebellar ataxia 3 [40]. Moreover, protein persulfidation regulates numerous biochemical processes via the allosteric influence on proteins [45].

In our work, we found that the H_2_S accumulation in *ETHE1* fibroblasts was associated with increased levels of protein persulfidation, and that CoC3 supplementation prevents both alterations. In *ETHE1* mutant cells, increased protein persulfidation may play two important roles as a protective mechanism. First, it may reduce the accumulation of free H_2_S, and, second, it may protect proteins from oxidative stress. However, increased and persistent protein persulfidation by continuous toxic H_2_S levels may have pathological consequences in cell function.

### 4.3. Mitochondrial Dysfunction, Iron/Lipofuscin Accumulation, and Lipid Peroxidation

Another pathological consequence of ETHE1 deficiency in mutant cells was mitochondrial dysfunction, iron/lipofuscin accumulation, and lipid peroxidation. The impairment of mitochondrial function, including increased ROS production and the dysregulation of lipid metabolism, may be one mechanism that contributes to alterations in cellular bioenergetics and is consistent with other studies in EE [46,47]. An increase in ROS production can induce mitochondrial dysfunction, in part through the oxidative damage to lipids (peroxidation) and oxidation of respiratory chain proteins [48]. Interestingly, mitochondrial dysfunction and excessive ROS production are common features of neurodegeneration [49].

Although the role of iron in lipid peroxidation has been extensively described [50,51,52,53,54,55], the effect of lipid peroxidation on iron overload has not been particularly addressed. For this reason, biological models of increased lipid peroxidation due to genetic or toxic causes are interesting to verify whether lipid oxidation per se may induce intracellular iron accumulation. In a recent publication, this relationship has been examined in cellular models of phospholipase-A2-associated neurodegeneration (PLAN) [56]. PLAN is a rare genetic neurodegenerative disease within the group of Neurodegeneration with Brain Iron Accumulation (NBIA) disorders, caused by mutations in the *PLA2G6* gene that encodes the calcium-independent Phospholipase A2 group VI (iPLA2β), an enzyme responsible for the scission of peroxidised fatty acids in the *sn*2-position of membrane glycerophospholipids [57,58]. Due to PLA2G6 mutations, lipid peroxidation plays a central role in the pathophysiology of PLAN [56,59,60]. In addition, iron accumulation has been described to be a relevant event in the pathophysiology of the disease [56,61]. The proposed hypothesis is that excessive lipid peroxidation in cell membranes damages lipids and proteins that alter both the normal function of organelles, such as mitochondria, and membrane-dependent processes such as vesicular traffic and autophagy/mitophagy. All these alterations can trigger iron accumulation in the form of iron-rich lipofuscin granules [62,63,64,65,66,67].

A growing body of evidence has reported disturbances in redox homeostasis in EE due to the increased generation of reactive species caused by the main accumulating metabolites. Regarding H_2_S, in vitro studies showed that this metabolite increases the malondialdehyde (MDA) [68] and F2-isoprostanes levels [69] in the brain of rodents, indicating lipid oxidative damage that was prevented by the exogenous supplementation of the antioxidants resveratrol and GSH [68].

Our findings in cellular models of EE suggest that the accumulation of toxic metabolites and mitochondrial dysfunction increase lipid peroxidation, which induces lipofuscinogenesis in mitochondria. Studies that describe that iron is probably accumulated in lipofuscin granules originating from damaged mitochondria have previously been reported [28,64,70]. In addition, lipofuscin granules recruit iron, resulting in a redox-active surface capable of catalysing the Fenton reaction and increasing the formation of free radicals [70], and, as a consequence, enhancing lipid peroxidation in a vicious cycle [71]. Another major and already demonstrated characteristic of lipofuscin is the ability to inhibit oxidised protein degradation by competitively binding and sequestering the proteasome [72].

Lipofuscin is a pigment by-product of breakdown intracellular catabolism frequently found inside the lysosomes and cytosol of ageing postmitotic cells [73]. Lipofuscin is a heterogeneous aggregate composed mainly of oxidised proteins and lipids. Metals such as iron, copper, zinc, aluminum, manganese, and calcium make up only 2% of lipofuscin [74]. Among them, iron is hypothesised to be the main source of free radicals via the Fenton reaction [75].

Lipofuscin accumulation is attributed to various hypotheses, primarily involving lysosomal and mitochondrial origins. Despite the interconnected nature of mitochondrial and lysosomal lipofuscinogenesis, lipofuscin can be generated through dysfunctional mitochondrial fission without alterations in the autophagosome–lysosome system [64,76]. The mitochondrial origin of lipofuscin is based on the fact that a mitochondrion contains a large reserve of cellular iron [77] and this iron contributes to mitochondrial lipid peroxidation, which alters the mitochondrial integrity and function. In turn, lipid peroxidation by-products disrupt the mitochondrial architecture and contribute to lipofuscin formation [78]. The literature describes alternative routes of lipofuscin formation in mitochondria due to lipid peroxidation, without lysosomal participation [64].

Our results in fibroblasts derived from the EE patient clearly showed that lipofuscin granules are formed in mitochondria in a similar way as other pathologies such as pantothenate-kinase-associated neurodegeneration (PKAN) and beta-propeller protein-associated neurodegeneration (BPAN) [28,79,80]. We proposed that mitochondrial dysfunction in EE induces ROS formation and the oxidation of lipids and iron-rich mitochondrial proteins. These oxidised compounds are aggregated in the form of lipofuscin granules which are finally released into the cytosol.

### 4.4. mtUPR Activation and Improvement

On a previous work, in order to find therapeutic candidates for mitochondrial diseases, we developed a screening culture medium in which cells bearing mitochondrial mutations could not survive unless they were treated with the right compounds [29]. Our screenings identified pterostilbene as a positive compound, whose efficacy was further boosted by the addition of nicotinamide and other common mitochondrial activity enhancers such as thiamine, riboflavin, L-carnitine, and lipoic acid. This combination (CoC3) activates SIRT3 and mtUPR, as well as enhance sirtuin levels and mitochondrial biogenesis and activity in several cell models of mitochondrial diseases [29].

In this work, we showed that this combination of compounds (CoC3) was also effective in correcting the main pathological alterations in the cellular model of EE. We also demonstrated that CoC3 activates mtUPR and that, specifically, SIRT3 activation is critical for its beneficial effects.

Sirtuins (SIRTs) are NAD+-dependent histone deacetylases that regulate the acetylation status of various proteins within the mitochondrial proteome. In addition, these enzymes are involved in modulating key metabolic pathways in both prokaryotic and eukaryotic organisms [81]. In mammalian cells, there are seven isoforms (SIRT1 to SIRT7), which are distributed in different cellular compartments: the nucleus (SIRT1, SIRT6, and SIRT7), the cytoplasm (SIRT2), and the mitochondria (SIRT3, SIRT4, and SIRT5).

SIRT3 is a key mitochondrial deacetylase that significantly contributes to the regulation of mitochondrial activity [82,83]; for instance, the removal of acetyl groups by SIRT3 from various subunits of complexes I to V in the mitochondrial electron transport chain indicates that this enzyme is essential for proper mitochondrial function [84]. SIRT3 also helps counteract oxidative stress by activating several antioxidant elements such as FOXO3A, IDH2, and MnSOD, which contributes to reducing or delaying oxidative damage and improves mitochondrial function and resilience [85,86]. In addition, the activation of sirtuins can stimulate mitochondrial biogenesis by increasing PGC-1α expression through SIRT3 and promoting its deacetylation through SIRT1 [87].

Although mtUPR activation pathways are not completely elucidated, several studies have correlated SIRT3 activation with mtUPR [88], suggesting it might be a key factor explaining its ability to increase one’s lifespan [89]. Indeed, recent animal model studies have verified the connection between sirtuins and mtUPR activation [90,91].

These investigations additionally indicated their role in sustaining mitochondrial proteostasis. The mtUPR not only is responsible for regulating faulty protein breakdown but also optimises the equilibrium of protein import and export within the mitochondrial space, while boosting the capacity for protein folding. [92]. These processes enhance mitochondrial performance and the general adaptation to cellular stress [93]. In this study, we could demonstrate that CoC3 activates mtUPR in *ETHE1* mutant fibroblasts, leading to a significant improvement in cellular bioenergetics. We suggest that the activation of mtUPR by CoC3 enhances both the expression and activity of chaperones, thereby stabilising the mutant protein. As a result, the mutant protein would not undergo immediate degradation, allowing it to persist within the cell with some remaining functionality. The slight increase in functionality of the aforementioned protein would be sufficient to significantly improve H_2_S metabolism and the pathological consequences of its accumulation. In this regard, the role of SIRT3 in improving the folding and stability of mitochondrial proteins has been well-documented [94,95]. The relevance of SIRT3 as a compensatory mechanism was confirmed, considering that its inhibition by 3-TYP suppresses the survival of *ETHE1* mutant cells in the galactose medium even with CoC3 treatment (Figure 18, Figures S13 and S14). In the future, these results should be confirmed by SIRT3 gene silencing.

## 5. Conclusions

In our work, we have identified a cocktail of mtUPR and mitochondrial booster agents that successfully partially restored the expression of the mutant enzyme, increased complex IV subunits, and significantly improved cell bioenergetics in *ETHE1* mutant cells. Additionally, cocktail supplementation reduced the H_2_S and protein persulfidation levels, as well as prevented iron overload and lipid peroxidation. Our findings indicate that the beneficial impact of the cocktail was mediated by the activation of SIRTs, especially SIRT3, and the promotion of antioxidant enzyme expression by triggering mtUPR, a crucial protective process in mitochondria. Therefore, the combination of pterostilbene, nicotinamide, riboflavin, thiamine, biotin, lipoic acid, and L-carnitine could be of help in correcting *ETHE1* mutations. Furthermore, we have demonstrated the value of personalised screenings in patient-derived cell models to assess how mutant cells behave under various therapeutic approaches and, consequently, to determine the best supplements and doses to use in controlled clinical trials. In future research, verifying our results using 3D systems (such as organoids) and animal models will be essential.

## Data Availability

The data supporting the findings of this study are not openly available due to reasons of sensitivity and to protect the privacy of individuals; however, the data are available from the corresponding author upon reasonable request. The data are stored in a controlled access data storage facility at Pablo de Olavide University (https://jazmin.upo.es/bscw/bscw.cgi, accessed on 22 December 2024).

## Supplementary Materials

The following supporting information can be downloaded at: https://www.mdpi.com/article/10.3390/antiox14060741/s1, Figure S1: ETHE1 localisation and expression levels; Figure S2: COX-IV localisation and expression levels; Figure S3: Levels of mitochondrial superoxide anion; Figure S4: Effects of CoC3 treatment on COX-IV localisation and expression levels; Figure S5: Effects of CoC3 treatment on COX-IV localisation and expression levels; Figure S6: Effects of CoC3 treatment on GSH levels; Figure S7: Effects of CoC3 treatment on LIP levels; Figure S8: Magnified images of TEM of EE patient’s fibroblasts; Figure S9: Effects of CoC3 treatment on the expression levels of iron-metabolism-related proteins; Figure S10: Effects of transfection with human *ETHE1* plasmid (cDNA) in ETHE1 expression levels; Figure S11: Effects of transfection with human *ETHE1* plasmid (cDNA) on COX-IV expression levels; Figure S12: Effects of cDNA complementation on iron accumulation; Figure S13: Effect of CoC3 supplementation on control and patient cells in glucose medium with EX-527 and 3-TYP after 0 h (T0) and 72 h (T72) of incubation; Figure S14: Effect of CoC3 supplementation on control and patient cells in stress medium (galactose + NaHS) with EX-527 and 3-TYP after 0 h (T0) and after 72 h (T72) of incubation; Figure S15: Effects of 3-TYP alongside treatment with CoC3 on expression levels of ETHE1 and COX-IV; Figure S16: Neuronal purity and conversion efficiency of iNs generated by direct reprogramming from control and EE patient’s fibroblasts; Figure S17: Effect of CoC3 supplementation on the expression levels of ETHE1 and COX-IV in fibroblasts from two EE patients; Figure S18: Effects of CoC3 supplementation on mitochondrial bioenergetics in fibroblasts from two EE patients; Figure S19: Effects of CoC3 treatment on lipid peroxidation in fibroblasts from two EE patients; Figure S20: Effect of CoC3 treatment on iron accumulation in fibroblasts from two EE patients.

## Abbreviations

3-TYP	3-(1H-1,2,3-triazol-4-yl) pyridine
α-LA	α-Lipoic acid
AMP	Adenosine monophosphate
AMPK	AMP-activated protein kinase
ATF4	Activating transcription factor 4
ATF5	Activating transcription factor 5
ATP	Adenosine triphosphate
BPAN	Beta-propeller protein-associated neurodegeneration
BSA	Bovine serum albumin
Calcein-AM	Calcein Acetoxymethyl ester
CBS	Cystathionine β-synthase
CO	Carbon monoxide
CoA	Coenzyme A
COX	Cytochrome c oxidase
COX-IV	Cytochrome c oxidase subunit IV
CSE	Cystathionine γ-lyase
Cy5	Cyanine 5
DAPI	4′,6-diamidino-2-phenylindole
DMEM	Dulbecco’s Modified Eagle Medium
DMSO	Dimethyl solfoxide
DMT1	Divalent Metal Transporter 1
DNA	Desoxyrribonucleic acid
DRP1	Dynamin-related protein 1
EDTA	Ethylene Diamine Tetraacetic Acid
EE	Ethylmalonic encephalopathy
eif2α	Eukaryotic translation initiation factor 2 alpha
P-eif2α	Phosphorilated eukaryotic translation initiation factor 2 alpha
EMA	Ethylmalonic acid
ER	Endoplasmic reticulum
ETHE1	Ethylmalonic encephalopathy 1
FBS	Fetal bovine serum
FCCP	Carbonyl cyanide-p-trifluoromethoxyphenylhydrazone
FM	Fluorescent marker
FTL	Ferritin light-chain
FXN	Frataxin
GPX4	Glutathione Peroxidase 4
GSH	Glutathione
GSSH	Glutathione persulfide
HBSS	Hanks’ Balanced Salt Solution
HEPES	4-(2-Hydroxyethyl)-1-piperazineethanesulfonic acid
HRP	Horseradish peroxidase
HSP60	Heat shock protein 60
HSP70	Heat shock protein 70
IC50	Half-maximal inhibitory concentration
ICP-MS	Inductively coupled plasma mass spectrometry
IDO1	indoleamine 2,3-dioxygenase 1
iNs	Induced neurons
ISCU	Iron–sulfur cluster assembly enzyme
LC	Liquid chromatography
LIP	Labile iron pool
Luperox^®^ DI	Luperox^®^ Diisopropyl
LYRM4	Lysine-rich motif containing 4
MetAP2	methionine aminopeptidase 2
MDA	malondialdehyde
Mfrn2	Mitoferrin 2
MnSOD	Manganese superoxide dismutase
MOI	Multiplicity of infection
MRI	Magnetic resonance imaging
MS	Mass spectrometry
3-MST	3-mercaptopyruvate sulfotransferase
mt-CO2	Mitochondrially encoded cytochrome c oxidase subunit 2
mt-FTL	Mitochondrial ferritin light chain
mt-ND1	Mitochondrially encoded NADH Dehydrogenase Subunit 1
NAD	nicotinamide adenine dinucleotide
NADH	Reduced nicotinamide adenine dinucleotide
NCOA4	Nuclear Receptor Coactivator 4
NDUFS8	NADH:Ubiquinone Oxidoreductase protein 8
NFS1	Nuclear Fe-S cluster protein 1
NRF2	Nuclear respiratory factor 2
C1-NBF	4-chloro-7-nitrobenzofurazan
NBIA	Neurodegeneration with Brain Iron Accumulation
NO	Nitric oxide
OCR	Oxygen consumption rate
OPA1	Optic atrophy 1
OR	Oxygen radicals
OXPHOS	Oxidative phosphorylation
PB	Phosphate buffer
PBS	Phosphate-buffered saline
PDH	Pyruvate dehydrogenase
PFA	Paraformaldehyde
PGK	Phosphoglycerate kinase
PKAN	Pantothenate-kinase-associated neurodegeneration
PLAN	Phospholipase-A2-associated neurodegeneration
PPB	Perl’s Prussian Blue
REST	RE1-Silencing Transcription factor
RNS	Reactive nitrogen species
ROS	Reactive oxygen species
RS	Reactive species
RSH	Cysteine residues
RSSH	Persulfided cysteine residues
RT	Room temperature
SCAD	Short-chain acyl-CoA dehydrogenase
SD	Standard deviation
SDS	Sodium Dodecyl Sulfate
SDS-PAGE	Sodium Dodecyl Sulfate Polyacrylamide Gel Electrophoresis
SH	Reduced sulfur
SIRT	Sirtuin
SIRT1	Sirtuin 1
SIRT3	Sirtuin 3
SOD1	Superoxide dismutase 1
SQOR	Sulfo-quinone oxidoreductase
SSH	Sulfane sulfur
TBTA	Tris(benzyltriazolylmethyl)amine
TCA	Tricarboxylic acid
TEM	Transmission electron microscopy
TfR	Tranferrin receptor
TTBS	Tris-Buffered Saline with Tween 20
UPLC	Ultra-Performance Liquid Chromatography
VDAC1	Voltage-dependent anion channel 1
