# Supplementary material for: Mitochondrial Unfolded Protein Response (mtUPR) Activation Improves Pathological Alterations in Cellular Models of Ethylmalonic Encephalopathy
Source: Antioxidants (Basel). 2025 Jun 16;14(6):741. doi: 10.3390/antiox14060741 (PMC12189359; doi:10.3390/antiox14060741)
Supplement: Supplementary file 1 [file antioxidants-14-00741-s001.zip › antioxidants-3629068-supplementary.pdf]

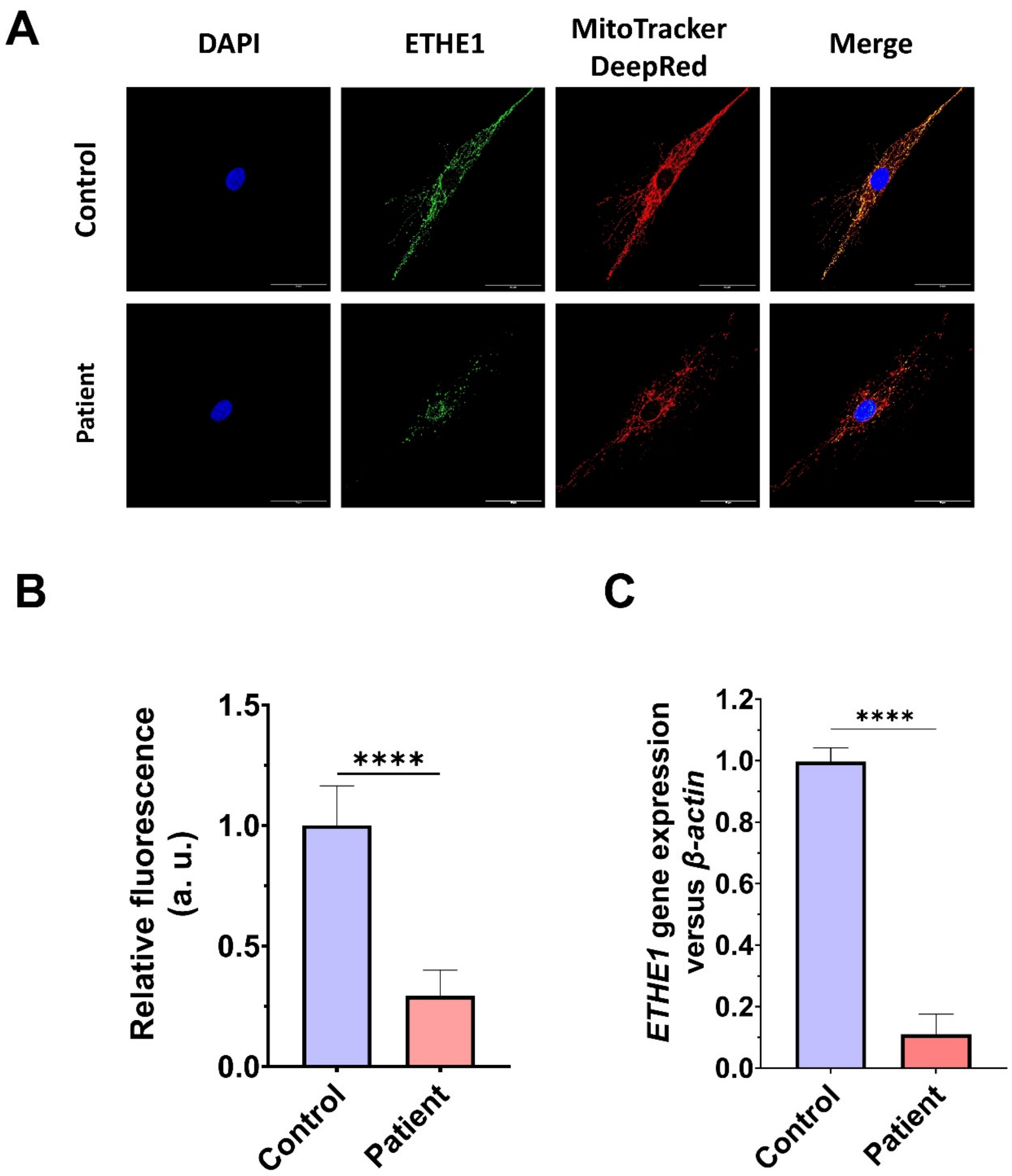

Figure S1. **ETHE1 localization and expression levels**. The immunofluorescence assay was performed in both control and patient fibroblasts. (**A**). Cells were fixed and immunostained with anti-ETHE1 antibody. MitoTracker™ DeepRed was used as a mitochondrial marker and nuclei were stained with DAPI. Scale bar: 50 µm. (**B**). Quantification of fluorescence intensity of the ETHE1 antibody. Images were analyzed by ImageJ software (at least 30 images were taken and analyzed from each condition and experiment). (**C**) *ETHE1* transcript levels in mutant (ETHE1) and control fibroblasts assessed by qPCR. Data were referred to control and represent the mean ± SD of 3 independent experiments. **** $p < 0.0001$ between control and patient fibroblasts. a.u.: arbitrary units.

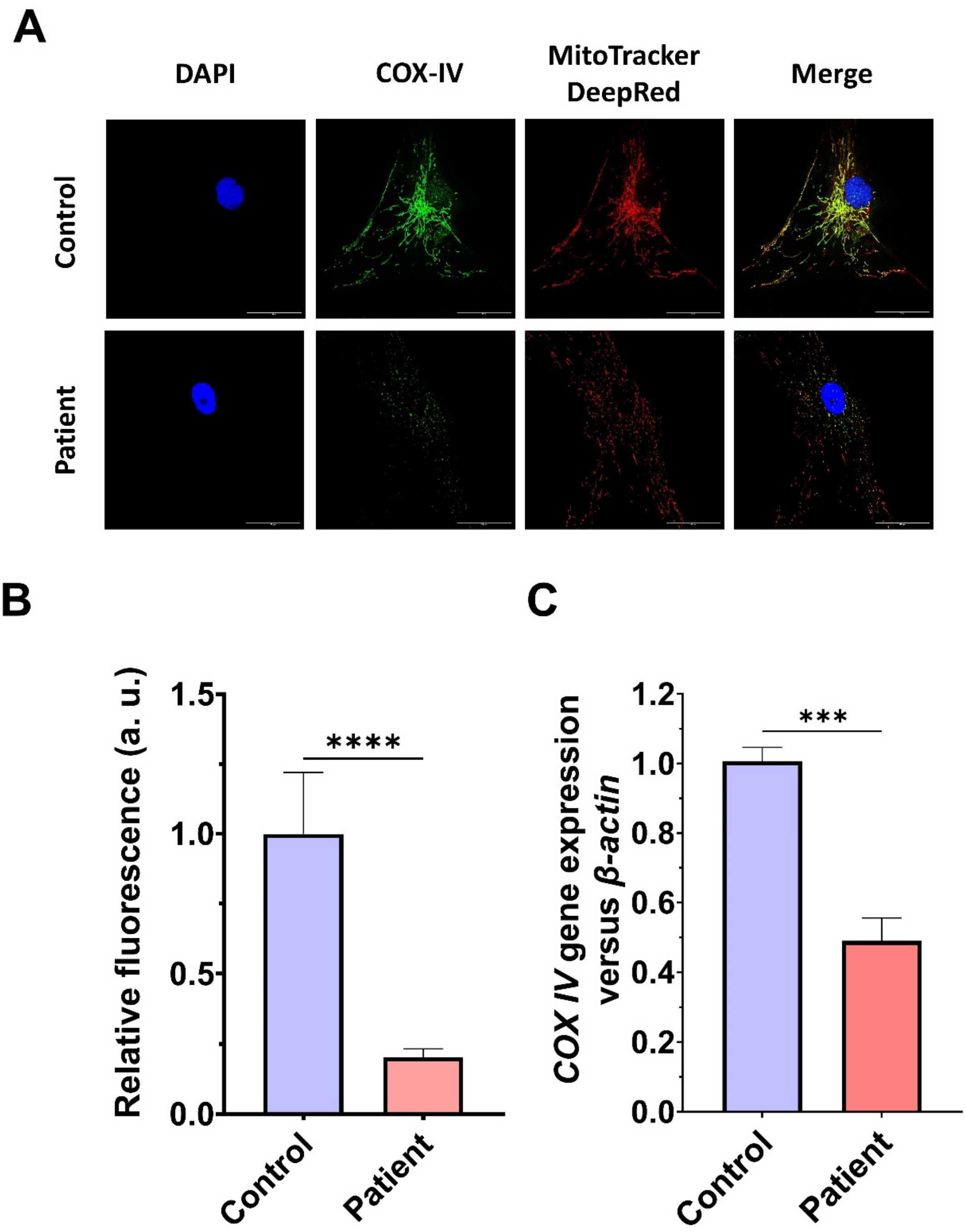

Figure S2. **COX-IV localization and expression levels**. The immunofluorescence assay was performed in both control and patient fibroblasts. (**A**). Cells were fixed and immunostained with anti-COX-IV antibody. MitoTracker™ DeepRed was used as a mitochondrial marker and nuclei were stained with DAPI. Scale bar: 50 µm. (**B**). Quantification of fluorescence intensity of the COX-IV antibody signal. (C) *COX-IV* transcript levels in mutant (ETHE1) and control fibroblasts assessed by qPCR. Images were analyzed by ImageJ software (at least 30 images were taken and analyzed from each condition and experiment). Data were referred to control and represent the mean ± SD of 3 independent experiments. **** $p < 0.0001$ between control and patient fibroblasts. a.u.: arbitrary units.

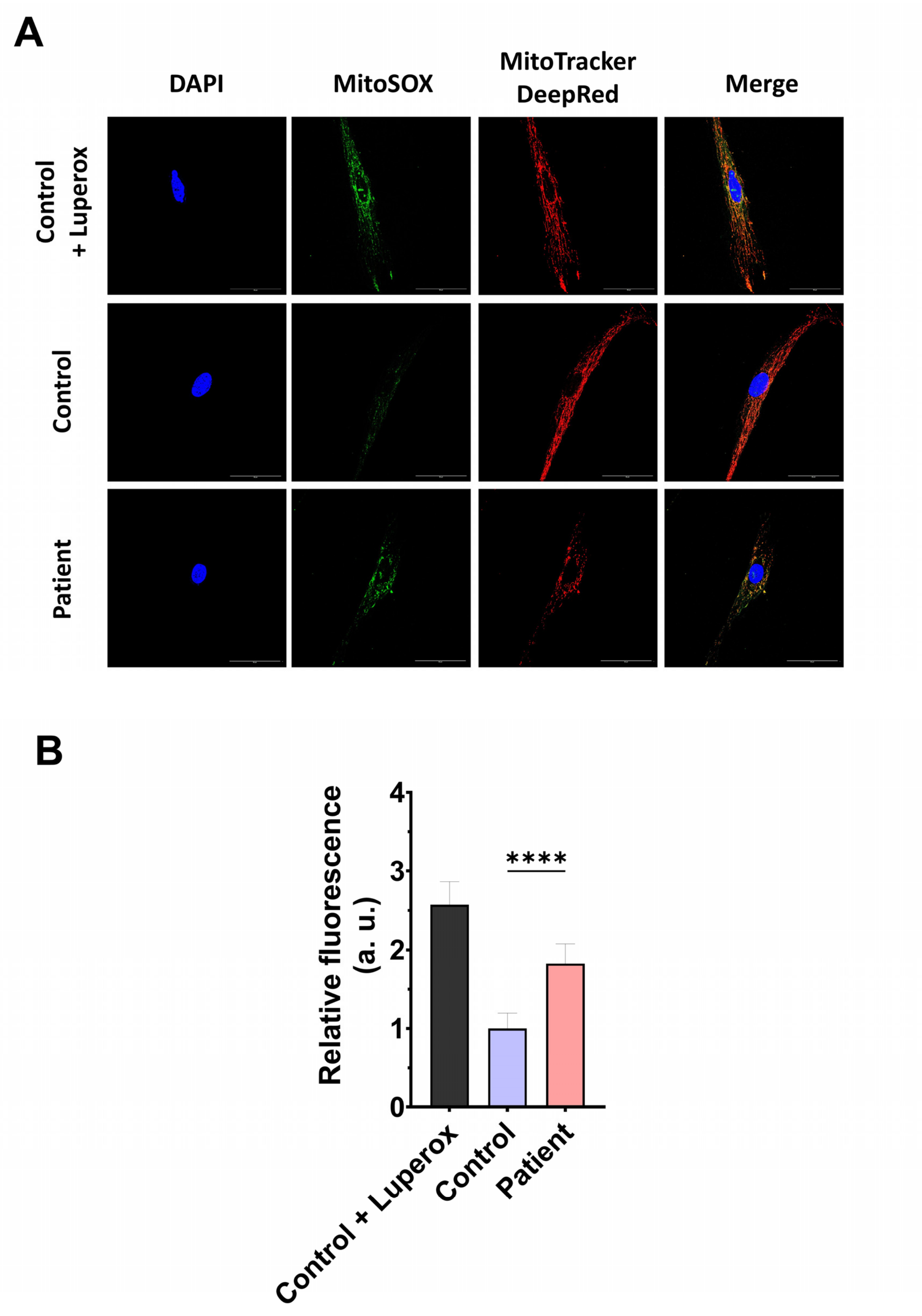

Figure S3. **Mitochondrial superoxide anion levels**. (**A**) Representative images of control and EE (patient) fibroblasts stained with MitoSOX™ Red and MitoTracker™ DeepRed. Nuclei were revealed by DAPI staining. Images were taken under a widefield fluorescence microscope using a 40× lens and processed by ImageJ software. Scale bar = 50 µm. (**B**) Fluorescence quantification of MitoSOX™ Red signal. Data were referred to control and represent the mean ± SD of 3 separate experiments (at least 30 images for each condition and experiment were analyzed). **** $p < 0.0001$ between control and patient fibroblasts. a.u.: arbitrary units.

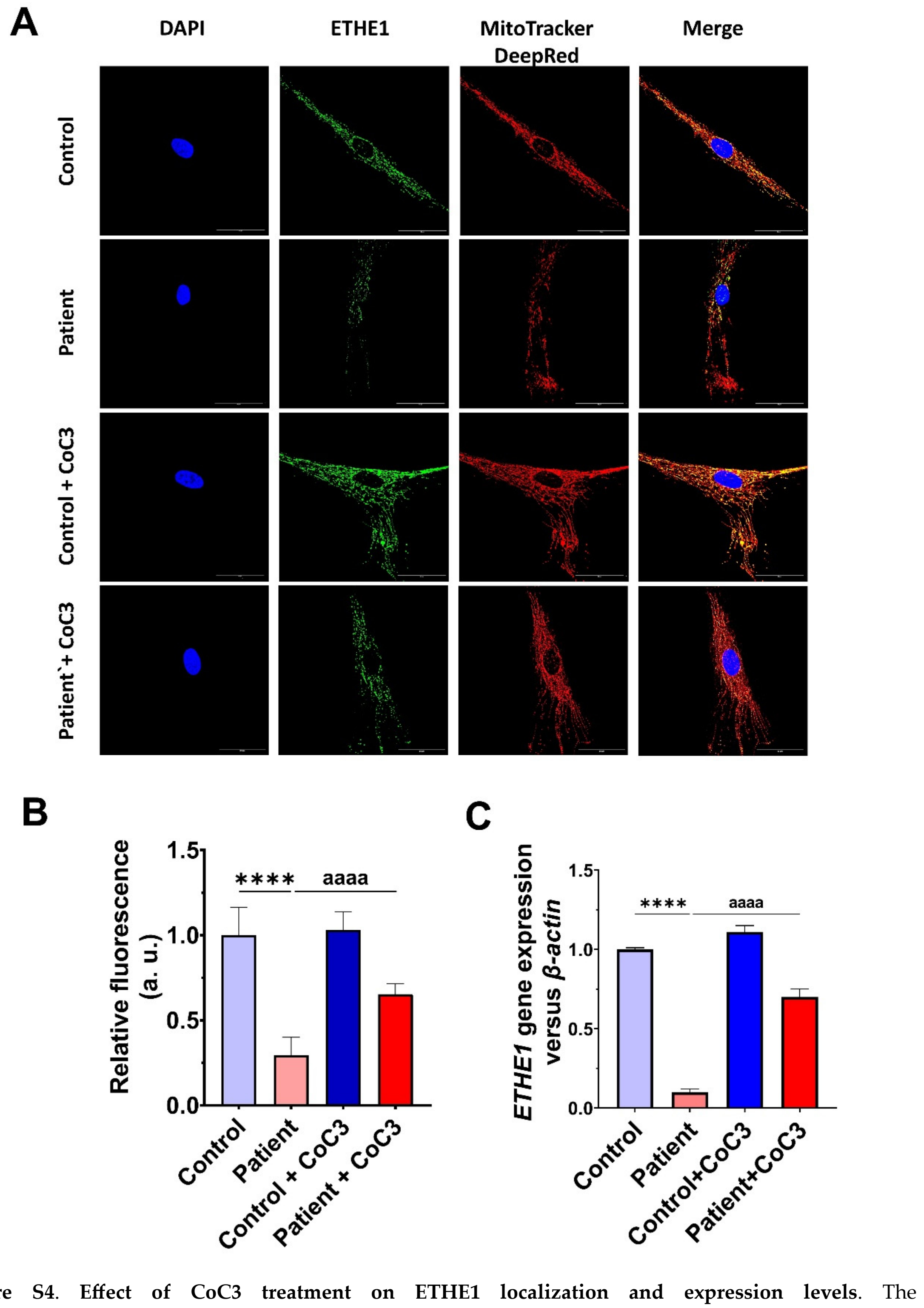

Figure S4. **Effect of CoC3 treatment on ETHE1 localization and expression levels**. The immunofluorescence assay was performed in control and EE patient fibroblasts untreated and treated for 7 days with CoC3. (**A**). Cells were fixed and immunostained with the anti-ETHE1 antibody. MitoTracker™ DeepRed was used as a mitochondrial marker and nuclei were stained with DAPI. Scale bar: 50 µm. (**B**). Quantification of fluorescence intensity of the ETHE1 antibody signal. Images were analyzed by ImageJ software (at least 30 images were taken and analyzed from each condition and experiment). (**C**) *ETHE1* transcript levels in CoC3 treated and untreated control and mutant ETHE1 fibroblasts assessed by qPCR. Data were referred to control and represent the mean ± SD of 3 independent experiments. **** $p < 0.0001$ between control and patient fibroblasts. ^{aaaa} $p < 0.0001$ between untreated and treated patient fibroblasts. a.u.: arbitrary units.

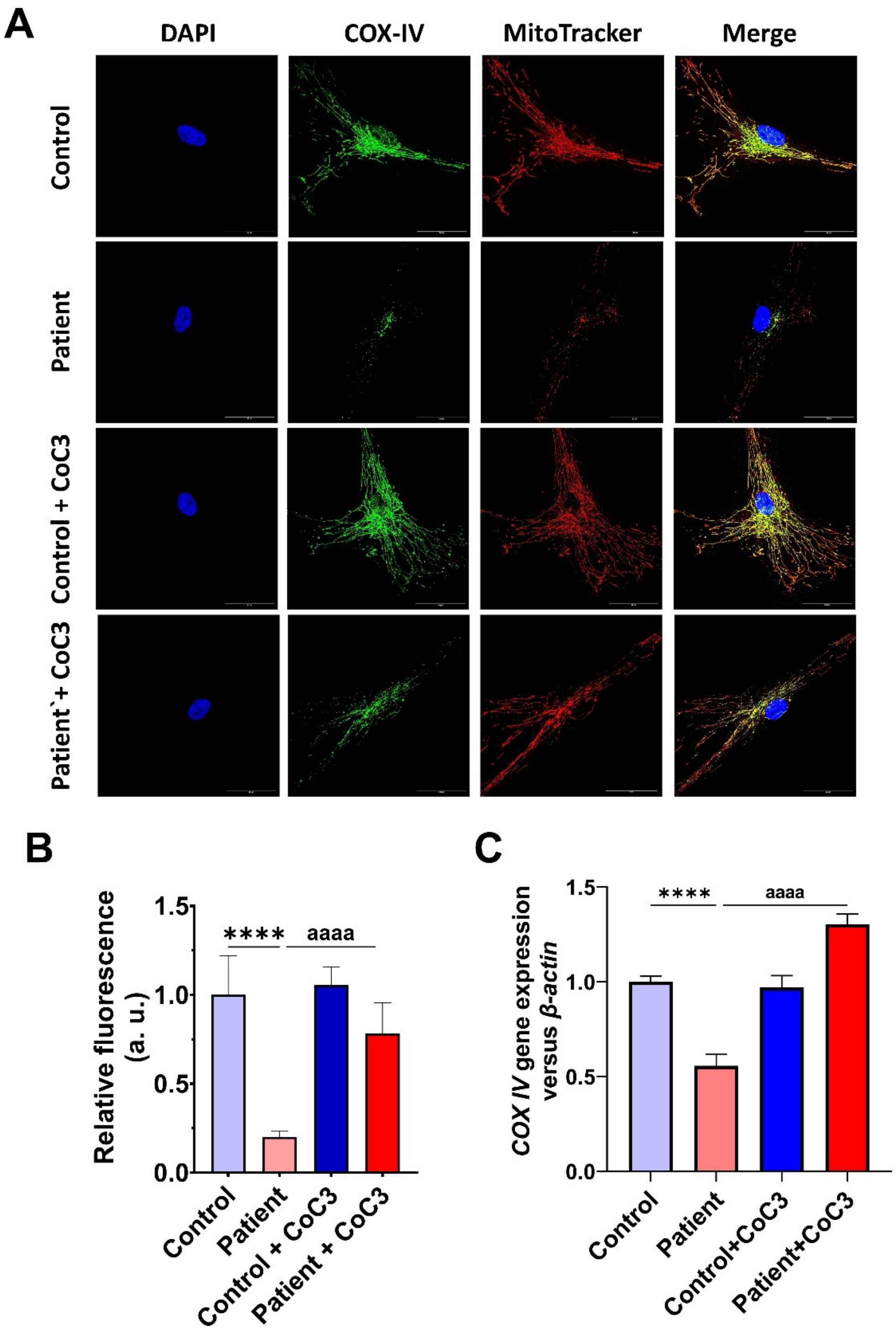

Figure S5. **Effects CoC3 treatment on COX-IV localization and expression levels. (A)** Immunofluorescence assay was performed in untreated and treated control and patient fibroblasts. Cells were fixed and immunostained with the anti-COX-IV antibody. MitoTracker™ DeepRed was used as a mitochondrial marker and nuclei were stained with DAPI. Scale bar: 50 µm. (**B**) Quantification of fluorescence intensity of the ETHE1 antibody signal. (C) *COX-IV* transcript levels in CoC3 treated and untreated control and mutant ETHE1 fibroblasts assessed by qPCR. Images were analyzed by ImageJ software (at least 30 images were taken and analyzed from each condition and experiment). Data were referred to control and represent the mean ± SD of 3 independent experiments. **** $p < 0.0001$ between control and patient fibroblasts. [aaaa] $p < 0.0001$ between untreated and treated patient fibroblasts. a.u.: arbitrary units.

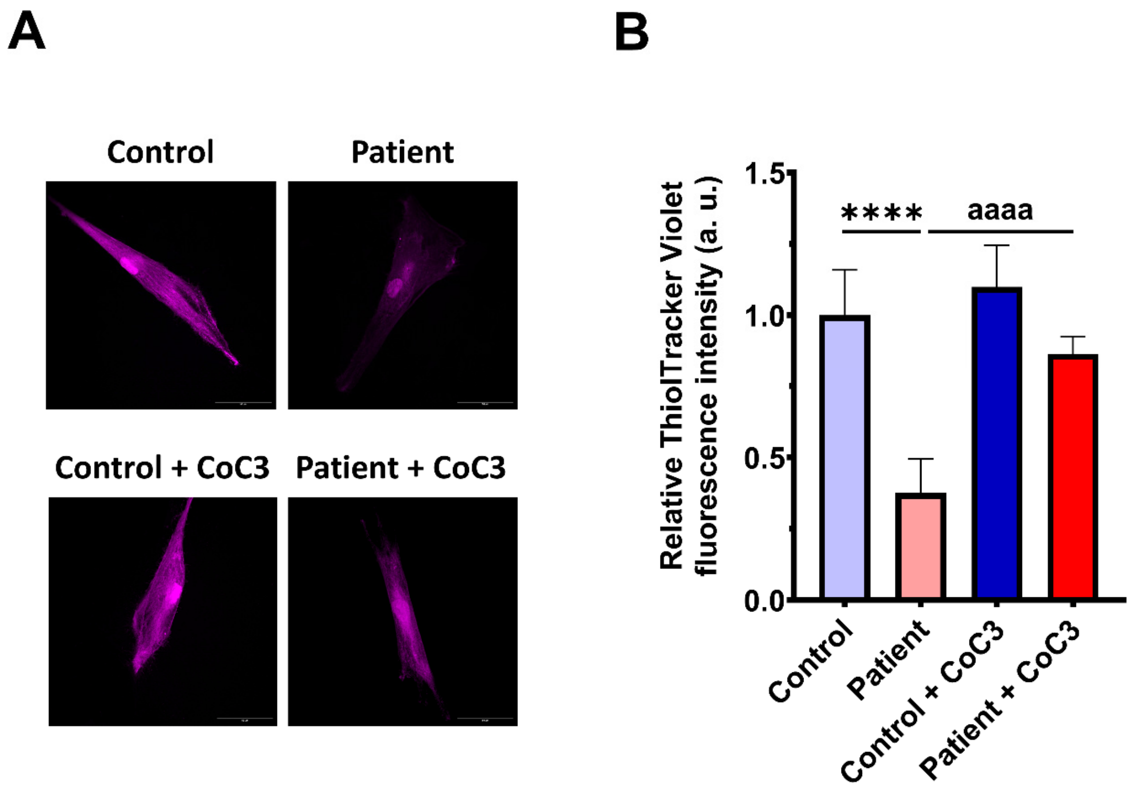

Figure S6. **Effects of CoC3 treatment on GSH levels.** (**A**) Representative images of GSH levels in untreated and treated control and patient fibroblasts using ThiolTrackerTM Violet staining. Scale bar: 50 µm. (**B**) Quantification of fluorescence intensity. Data were referred to control and represent the mean ± SD of 3 independent experiments. ****p<0.0001 between the control and patient fibroblasts. [aaaa] p< 0.0001 between untreated and treated patient fibroblast. a.u.: arbitrary units.

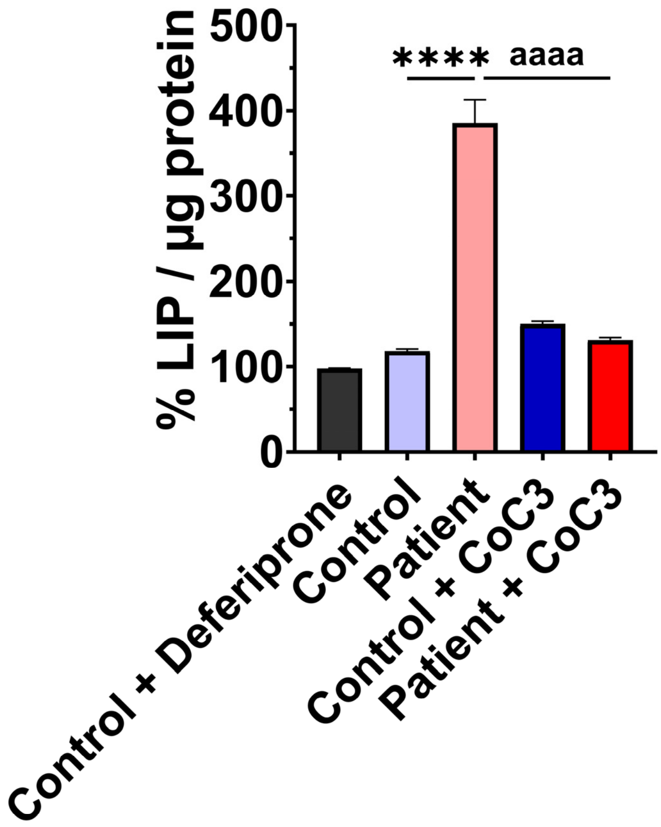

Figure S7. **Effects of CoC3 treatment on LIP levels**. Calcein assay was performed as described in Materials and Methods in control and patient fibroblasts. Control cells treated with 100 µM deferiprone were used as negative control. Data represent the mean ± SD of 3 independent experiments. ****$p < 0.0001$ between control and patient fibroblasts. [aaaa] $p < 0.0001$ between untreated and treated patient fibroblasts. a.u.: arbitrary units.

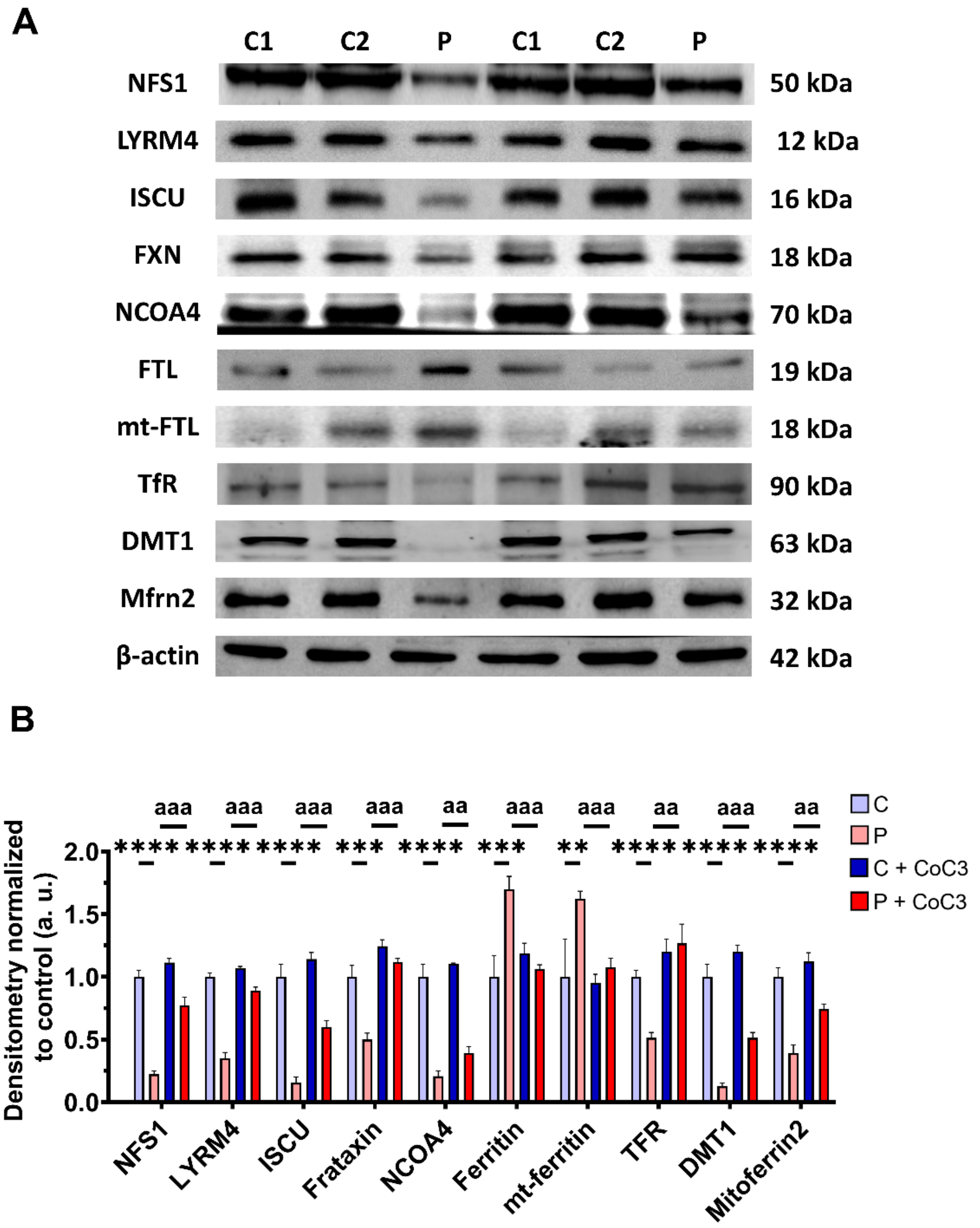

Figure S8. **Effects of CoC3 treatment on the expresión levels of iron metabolism-related proteins**. **(A)** Cellular extracts from untreated and treated control (C1 and C2) and patient fibroblasts (P) were analyzed by Western blot. Membranes were immunostained using antibodies against NFS1, LYRM4, ISCU, FXN, NCOA4, FTL, mt-FTL, TfR, DMT1, and Mfrn2. Expression levels of β-actin were used as a loading control. **(B)** Densitometry of Western blotting. Untreated and treated control samples were unified in one value for each condition (C and C + CoC3 respectively) representing the mean of each experimental condition. Densitometry was referred to untreated control (C) value. Data were referred to control and represent the mean ± SD of 3 independent experiments. ** $p < 0.01$, *** $p < 0.001$ and **** $p < 0.0001$ between control and patient fibroblasts. [aa] $p < 0.01$ and [aaa] $p < 0.001$ between untreated and treated patient fibroblasts. a.u.: arbitrary units.

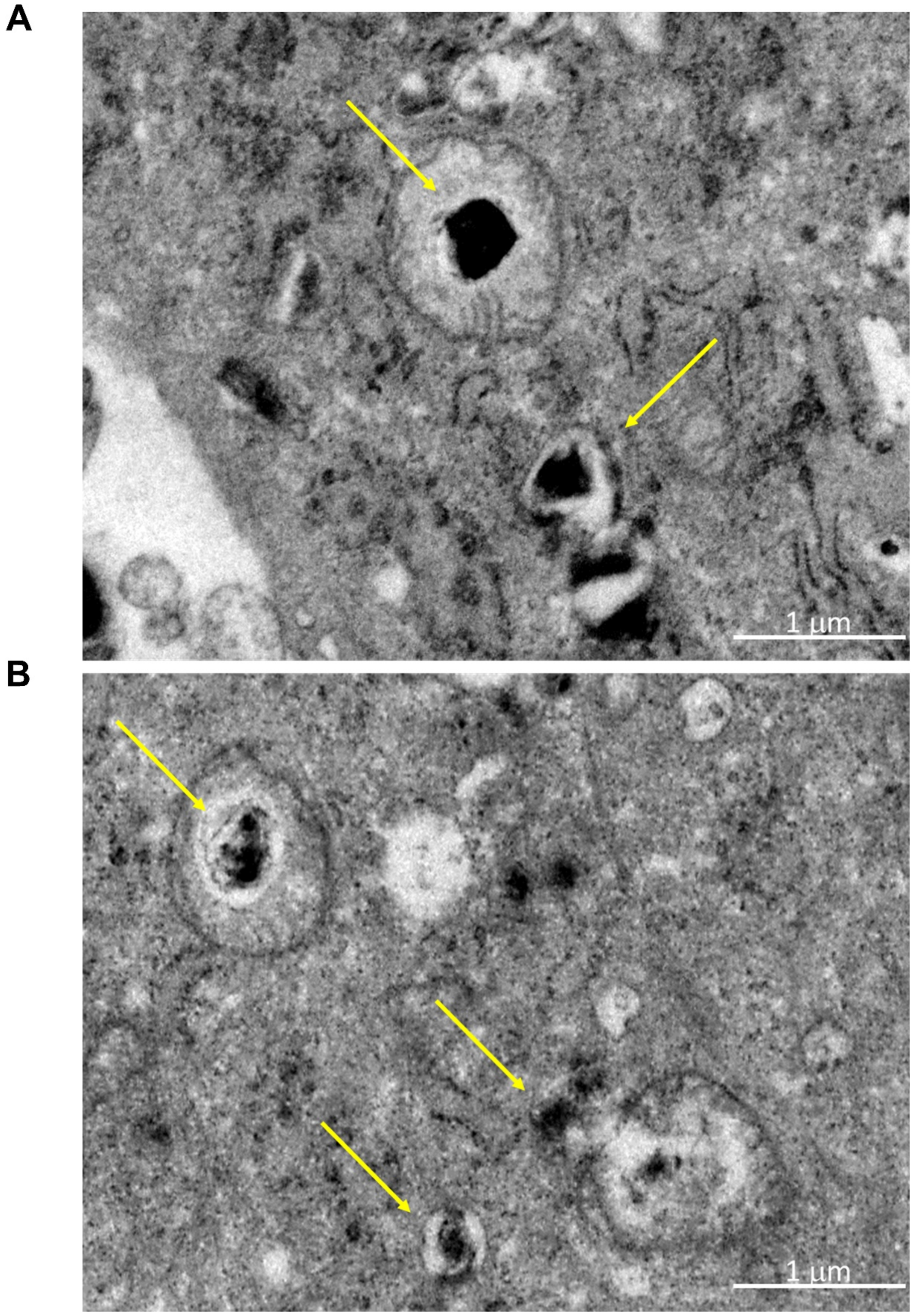

Figure S9. **Magnified TEM images of EE patient fibroblasts**. (**A, B**). Representative images of lipofuscin-like granules formed inside mitochondria. Scale bar: 1 μm. Yellow arrows: lipofuscin-like granules.

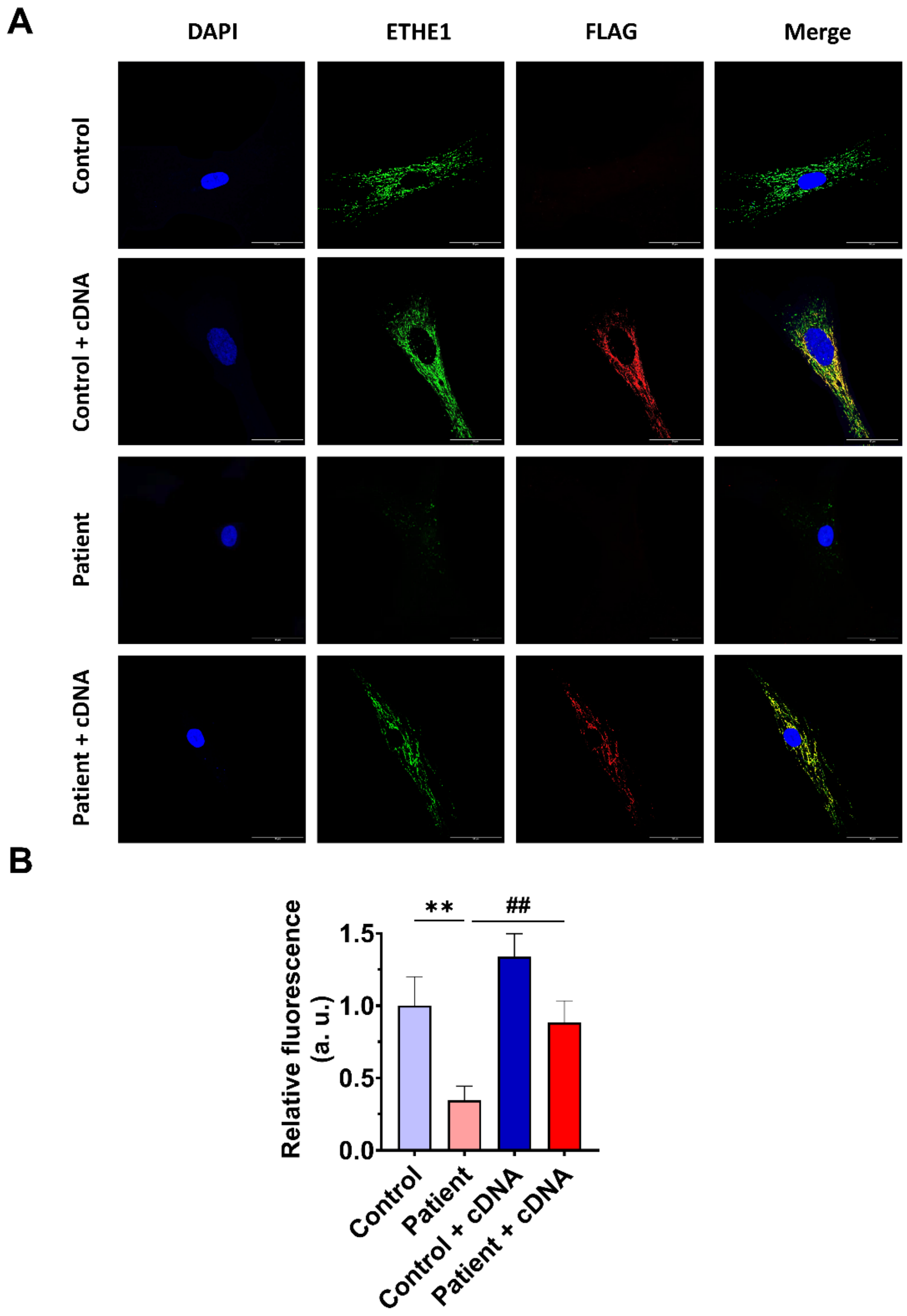

Figure S10. **Effects of transfection with human ETHE1 plasmid (cDNA) on ETHE1 expression levels.** Control and patient fibroblasts transfected and untransfected with human ETHE1 plasmid (cDNA) were fixed and inmunostained with anti-ETHE1 and anti-DYKDDDDK tag (FLAG) antibodies. **(A)** Representative images were acquired with DeltaVision microscope. DAPI was used to stain nuclei. Scale bar: 50 µm. **(B)**. Quantification of fluorescence intensity of ETHE1 antibody signal. Data were referred to control and represent the mean ± SD of 3 separate experiments (at least 30 images for each condition and experiment were analyzed). ** $p < 0.01$ between control and patient fibroblasts. $^{\#\#}p<0.01$ between patient fibroblasts with and without cDNA complementation. a.u.: arbitrary units.

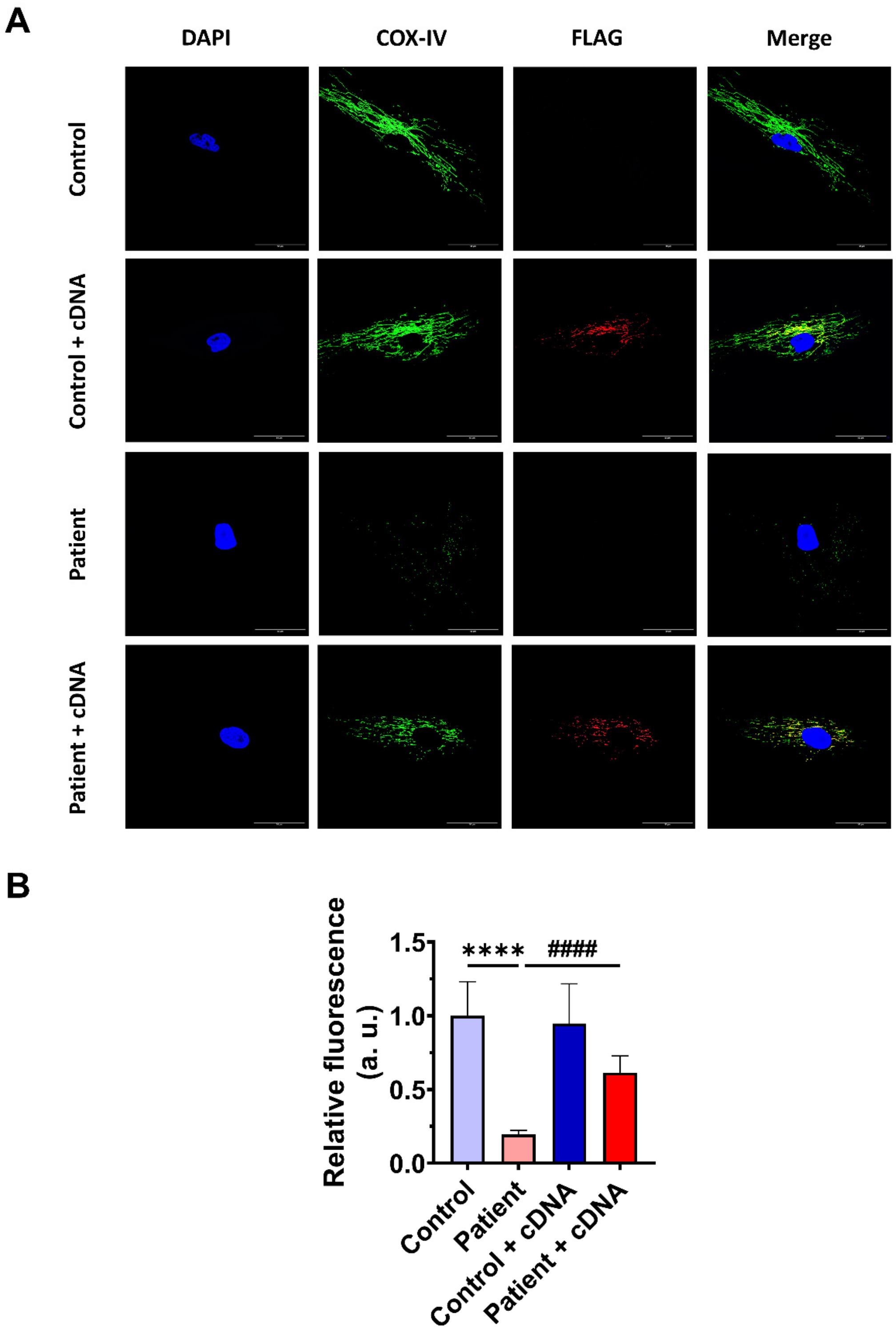

Figure S11. **Effects of transfection with human ETHE1 plasmid (cDNA) on COX-IV expresión levels**. Control and patient fibroblasts transfected and untransfected with human ETHE1 plasmid (cDNA) were fixed and inmunostained with anti-COX-IV and anti-DYKDDDDK tag (FLAG) antibodies. **(A)** Representative images were acquired with DeltaVision microscope. DAPI was used to stain nuclei. Scale bar: 50 µm. **(B)**. Quantification of fluorescence intensity of COX-IV antibody signal. Data were referred to control and represent the mean ± SD of 3 separate experiments (at least 30 images for each condition and experiment were analyzed). **** $p < 0.0001$ between control and patient fibroblasts. $^{####}$ $p<0.0001$ between patient fibroblasts with and without cDNA complementation. a.u.: arbitrary units.

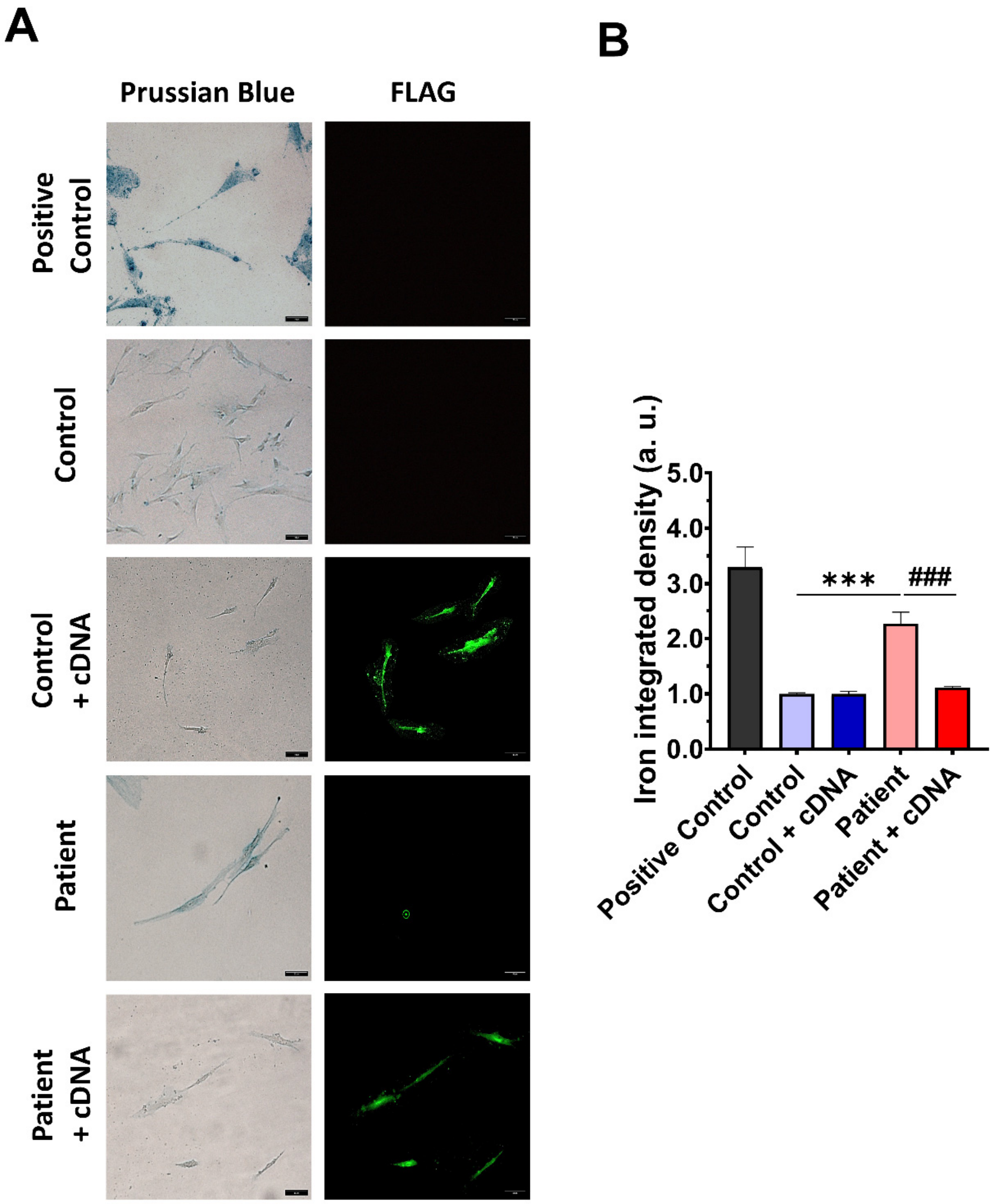

Figure S12. Effect of cDNA complementation on iron accumulation. (**A**) Control and EE patient's cells transfected and untransfected with human ETHE1 plasmid (cDNA) were stained with Prussian blue staining. A PKAN (pantothenate kinase-associated neurodegeneration) cell line was used as a positive control of iron accumulation. Images were acquired by Zeiss Axio Vert V1 microscope. Scale bar: 50 µm. (**B**) Quantification of Prussian Blue staining integrated density. Images were analyzed by the ImageJ software (at least 30 images were analyzed per each condition and experiment). Data represent the mean±SD of 3 independent experiments. *** $p < 0.001$ between control and patient fibroblasts. $^{###}$ $p < 0.001$ between patient fibroblasts with and without cDNA complementation. a.u.: arbitrary units.

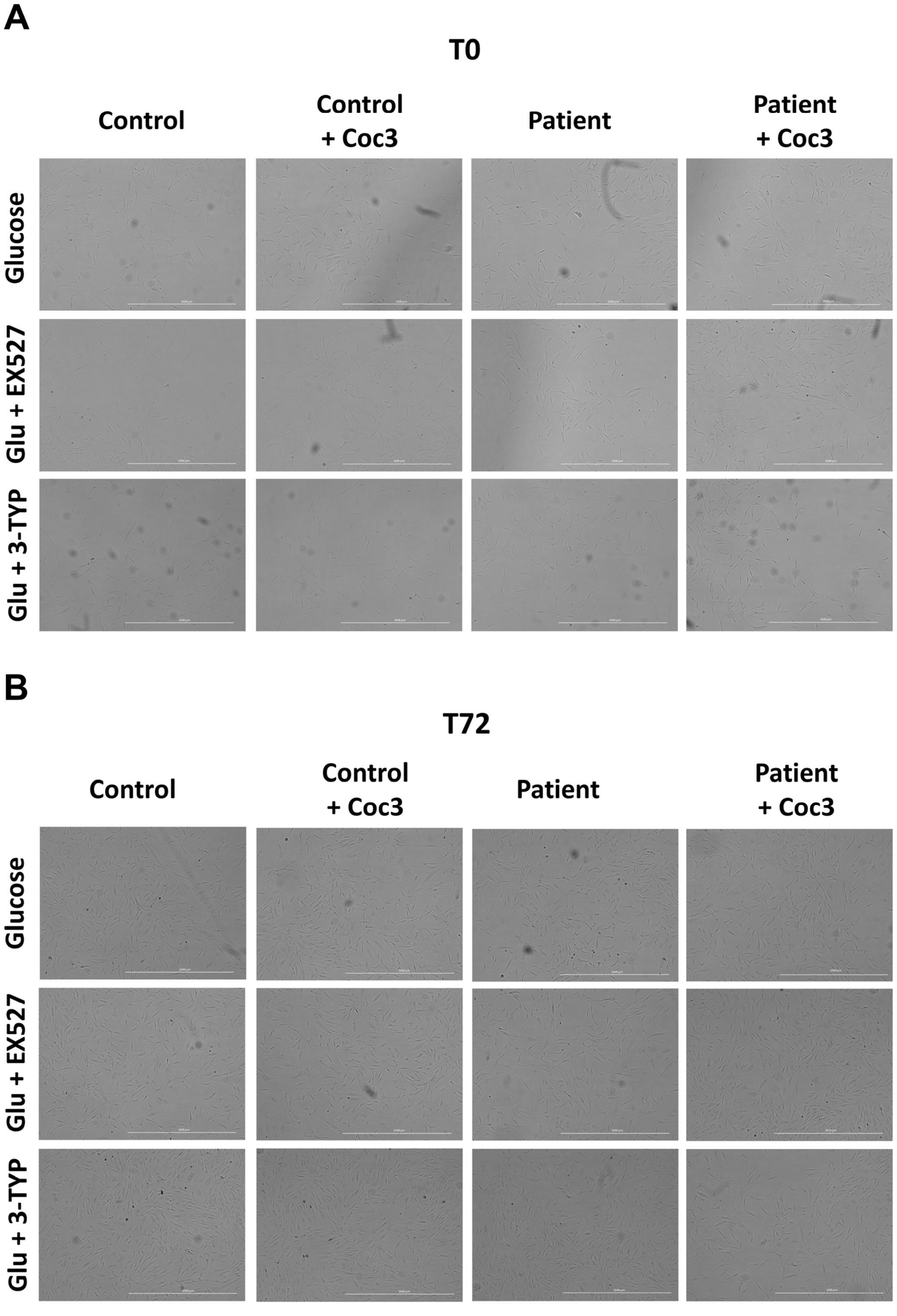

Figure S13. Effect of CoC3 supplementation on control and patient cells in glucose medium with EX-527 and 3-TYP after 0h (T0) and 72h (T72) of incubation. Cells were seeded in glucose medium and treated with CoC3 for seven days along with 150 nM EX-527 and 50 nM 3-TYP separately (added the last 72h of the treatment). (**A**) Representative images of control and patient fibroblasts. Photos were taken at that moment of the addition of EX-527 and 3-TYP (T0). **(B)** Representative images of control and patient fibroblasts in the pharmacological screening. Photos were taken 72h after the moment of the addition of EX-527 and 3-TYP (T72). Scale bar=1000 µm.

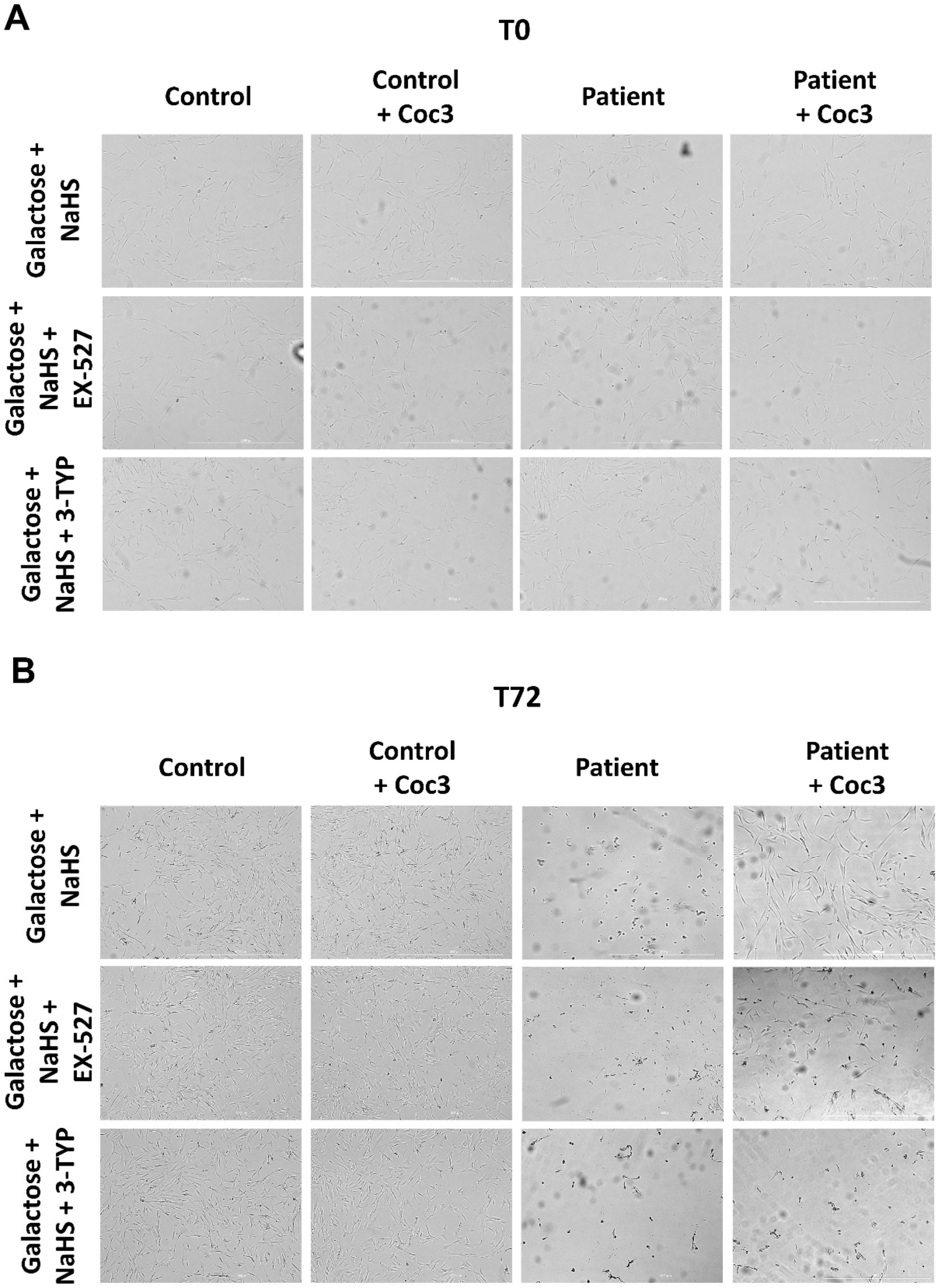

Figure S14. Effect of CoC3 supplementation on control and patient cells in stress medium (galactose + NaHS) with EX-527 and 3-TYP after 0h (T0) and after 72h (T72) of incubation. Cells were seeded in glucose medium and treated with CoC3 for seven days along with 150 nM EX-527 and 50 nM 3-TYP separately (added the last 72h of the treatment). Then, glucose medium was changed to stress medium (galactose with NaHS) and the two inhibitors. (**A**) Representative images of control and patient fibroblasts. Photos were taken at that moment of the medium change and the addition of EX-527 and 3-TYP (T0). (**B**) Representative images of control and patient fibroblasts. Photos were taken 72h after the moment of the medium change and the addition of EX-527 and 3-TYP (T72). Scale bar=1000 µm.

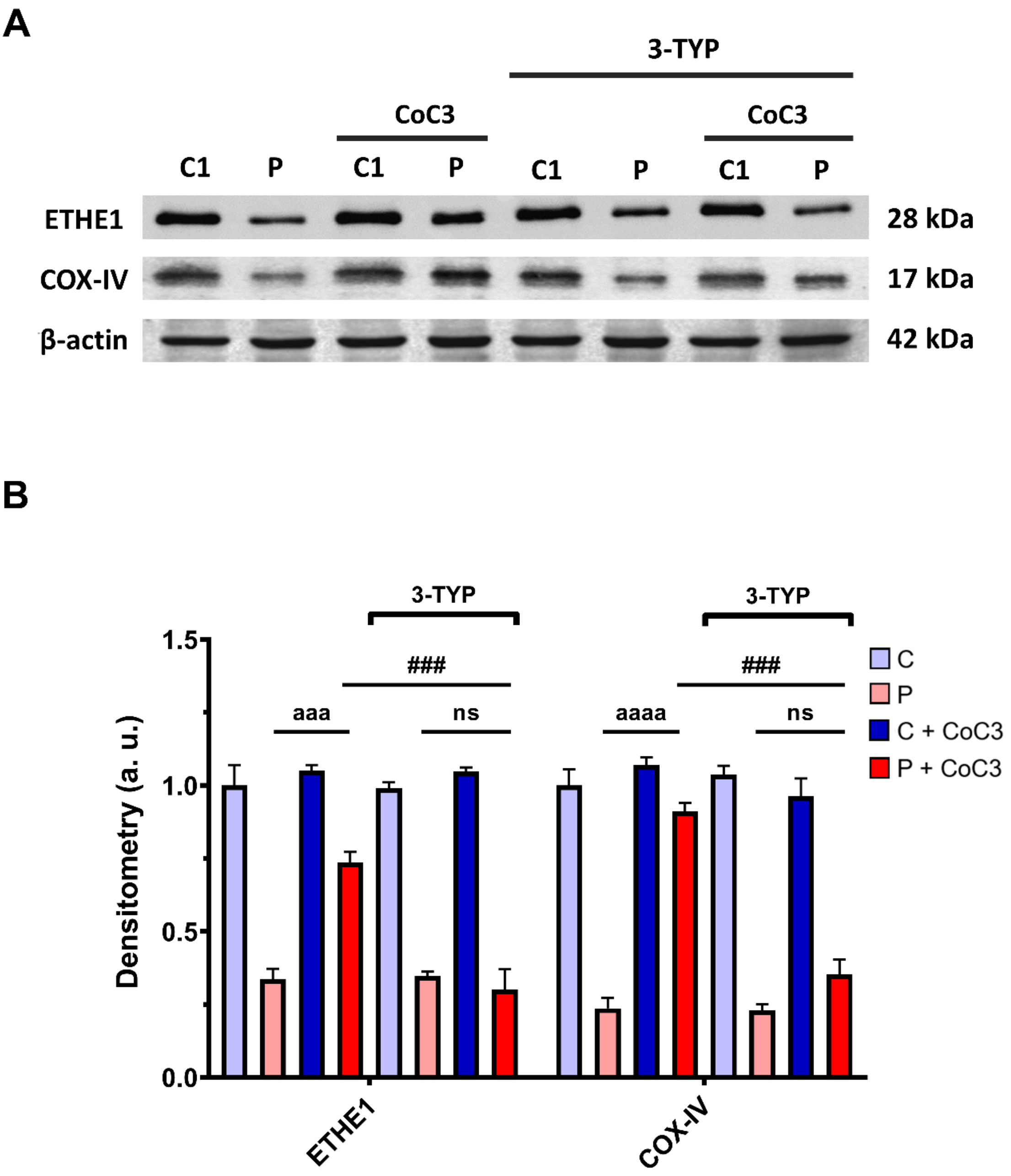

Figure S15. **Effects of 3-TYP alongside treatment with CoC3 for seven days on the expression levels of ETHE1 and COX-IV. (A)** Cellular extracts from control (C1) and EE patient fibroblasts (P), untreated and treated with CoC3 and CoC3 plus 3-TYP were examined by Western blot. Membranes were immunostained using antibodies against ETHE1, COX-IV and β-actin, the latter used as a loading control. **(B)** Densitometry of Western blotting. Densitometry was referred to untreated control (C1) value. ^{aaa} $p < 0.001$, ^{aaaa} $p < 0.0001$ between untreated and treated patient fibroblasts. $^{\#\#\#}$ $p < 0.001$ between patient fibroblasts treated with CoC3 and CoC3 plus 3-TYP; ns, non-significative; a.u.: arbitrary units.

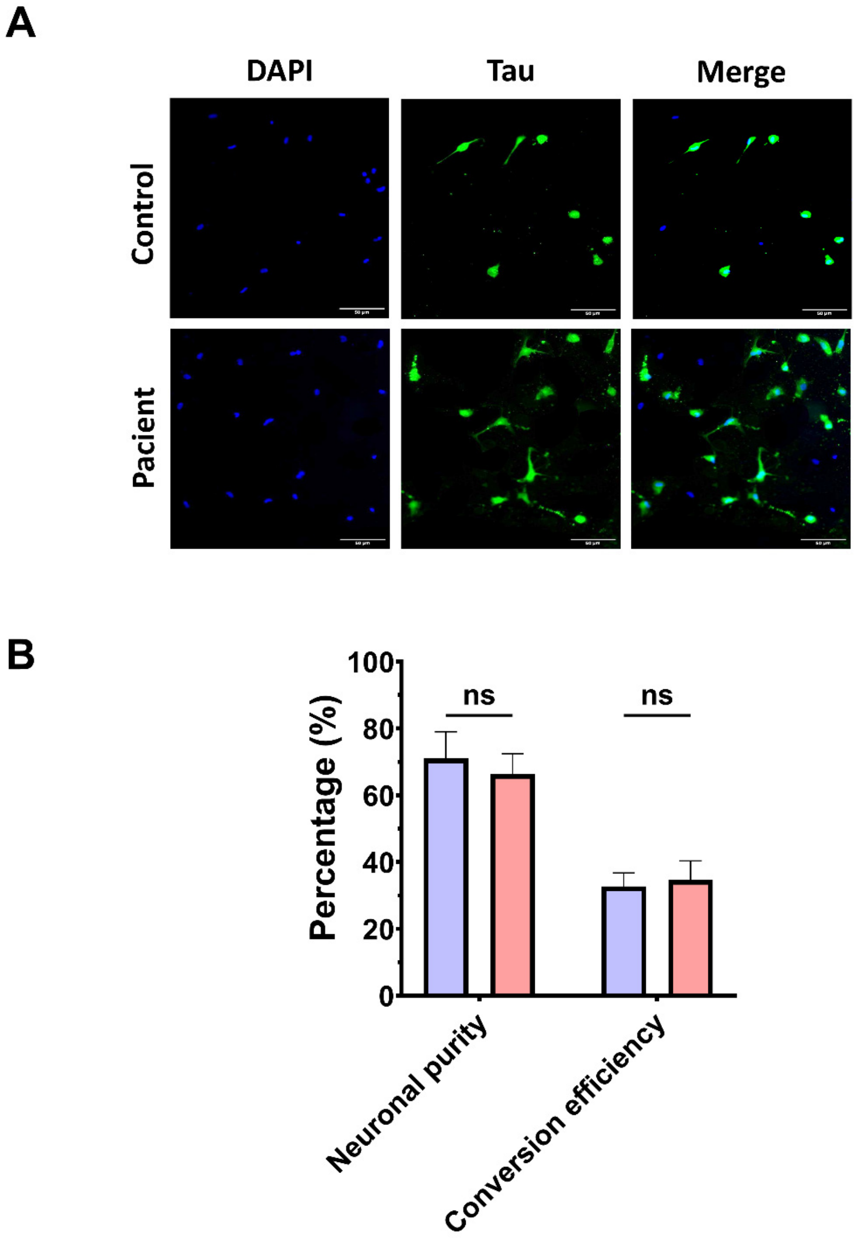

Figure S16. **Neuronal purity and conversion efficiency of iNs generated by direct reprogramming from control and EE patient's fibroblasts. (A)** Representative images of control and patient iNs marked with anti-Tau, a neuronal marker. Undifferentiated cells showed only DAPI staining for the nuclei. Scale bar: 50 μm. **(B)** Neuronal purity: number of Tau+ cells over the total of cells after reprogramming. **(C)** Conversion efficiency: number of Tau+ cells over the total of cells seeded at the beginning of the assay; ns non-significative.

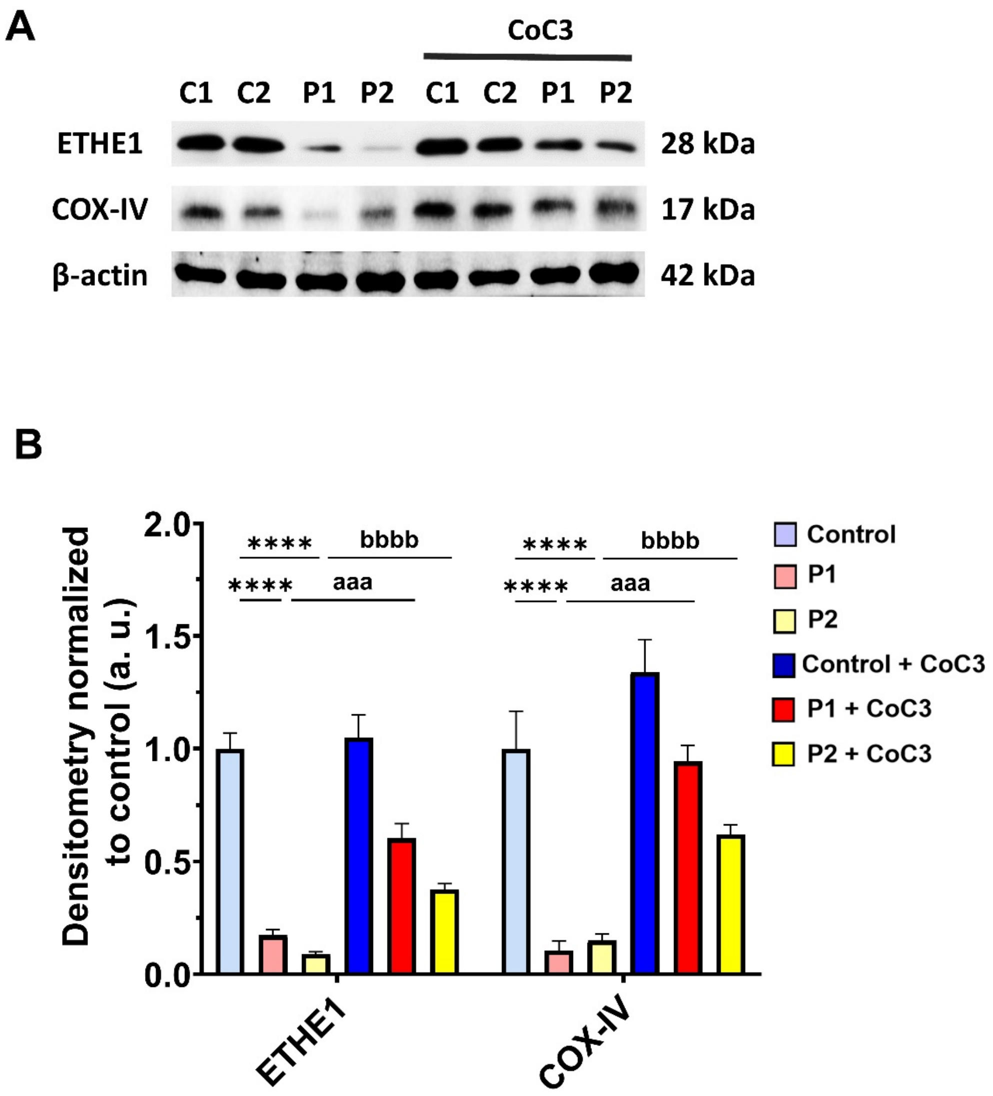

Figure S17. **Effect of CoC3 supplementation on the expression levels of ETHE1 and COX-IV in fibroblasts from two EE patients.** Cells were treated with CoC3 for seven days. **(A)** Cellular extracts from untreated and treated control (C1 and C2) and EE patient fibroblasts (P1 and P2) were examined by Western blot. Membranes were immunostained with antibodies against ETHE1, COX-IV and β-actin, the latter was used as a loading control. **(B)** Densitometry of Western blotting. Untreated and treated control samples were unified in one value for each condition (C and C + CoC3 respectively) which represent the mean of each experimental condition. Densitometry was referred to untreated control (C) value. Data represent the mean ± SD of 3 independent experiments. **** $p < 0.0001$ between the control and patient fibroblasts. [aaaa] $p < 0.0001$ between untreated and treated patient fibroblasts. a.u.: arbitrary units.

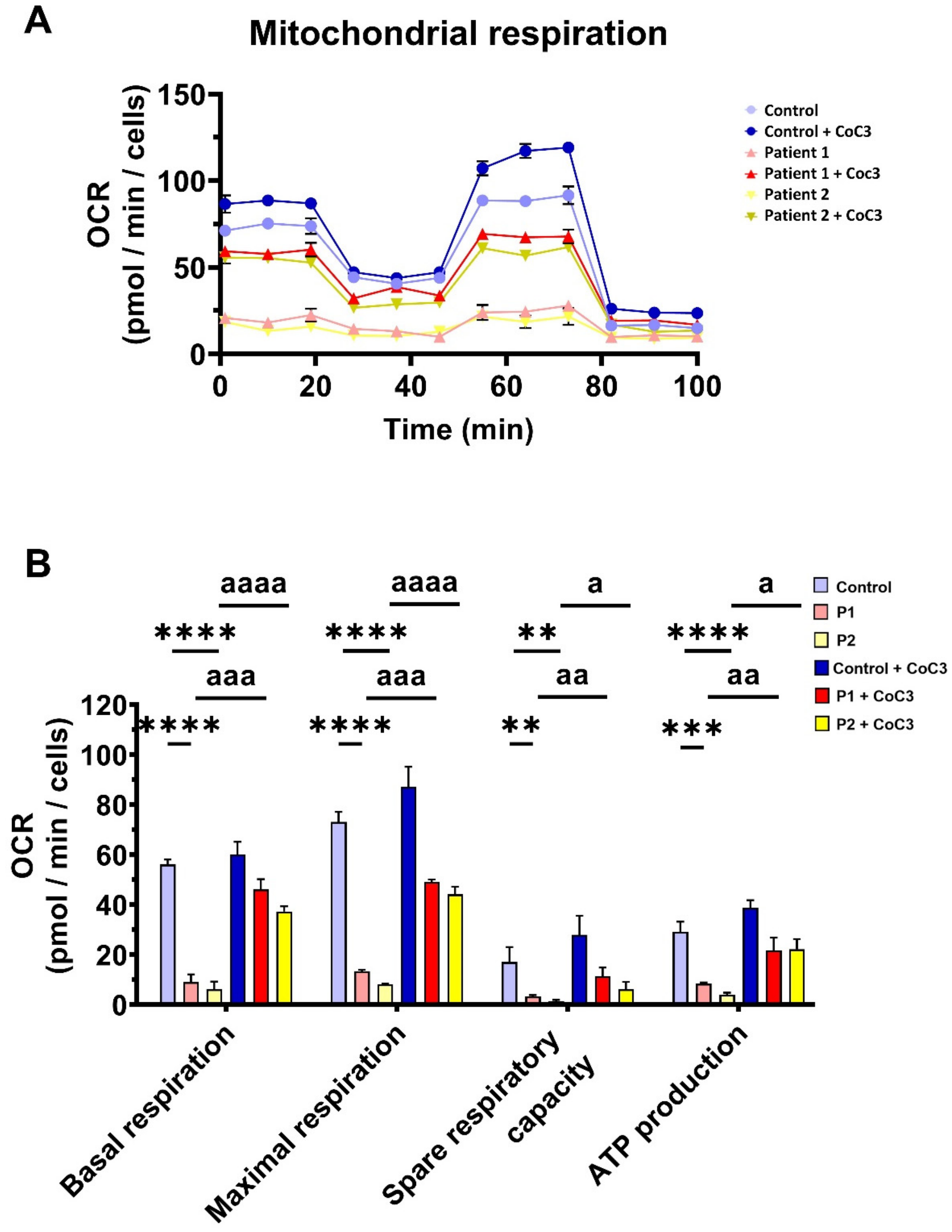

Figure S18. **Effects of CoC3 supplementation on mitochondrial bioenergetics in fibroblasts from two EE patients**. **(A)** Mitochondrial bioenergetics of control and two patient fibroblasts (P1 and P2) cells using the Seahorse analyzer and normalized to 15,000 cells as described in the Material and Methods. **(B)** Values of basal and maximal respiration, spare respiratory capacity and mitochondrial ATP production. ** $p < 0.01$ *** $p < 0.001$ and **** $p < 0.0001$ between the control and patient fibroblasts. ^{a} $p < 0.05$, ^{aa} $p < 0.01$, ^{aaa} $p < 0.001$ and ^{aaaa} $p < 0.0001$ between untreated and treated patient fibroblasts.

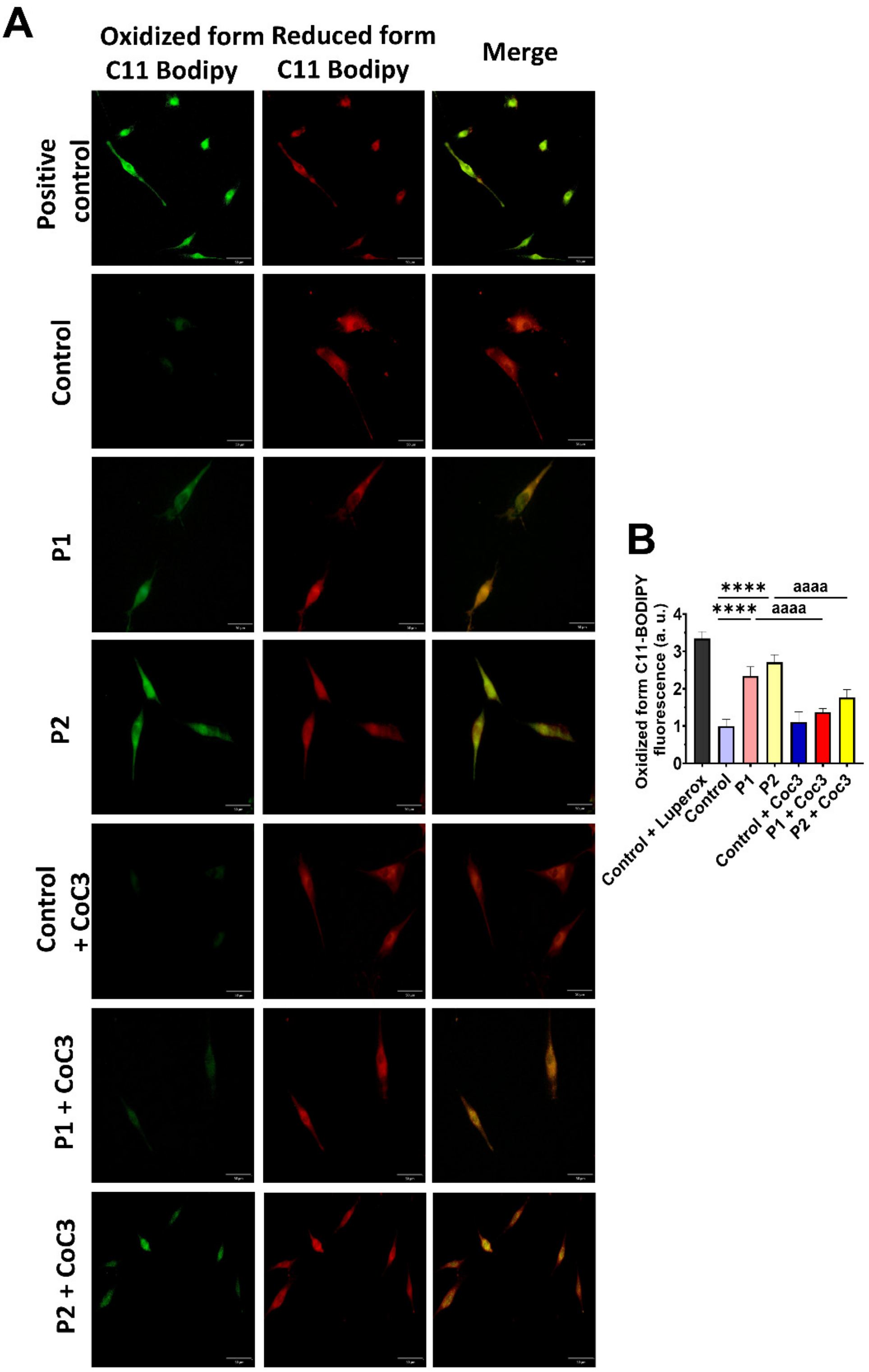

Figure S19. **Effects of CoC3 treatment on lipid peroxidation in fibroblasts from two EE patients**. (**A**) Representative images of lipid peroxidation with BODIPY 581/591 C11 staining of untreated and treated with CoC3 control and EE patient fibroblasts (P1 and P2). Control cells treated with Luperox at 500 μM were used as positive control. Scale bar: 50 μm. **(D)** Quantification of fluorescence intensity. Data were referred to control and represent the mean ± SD of 3 independent experiments. **** $p < 0.0001$ between control and EE patient fibroblasts. [aaaa] $p < 0.0001$ between untreated and treated EE patient fibroblasts. a.u.: arbitrary units.

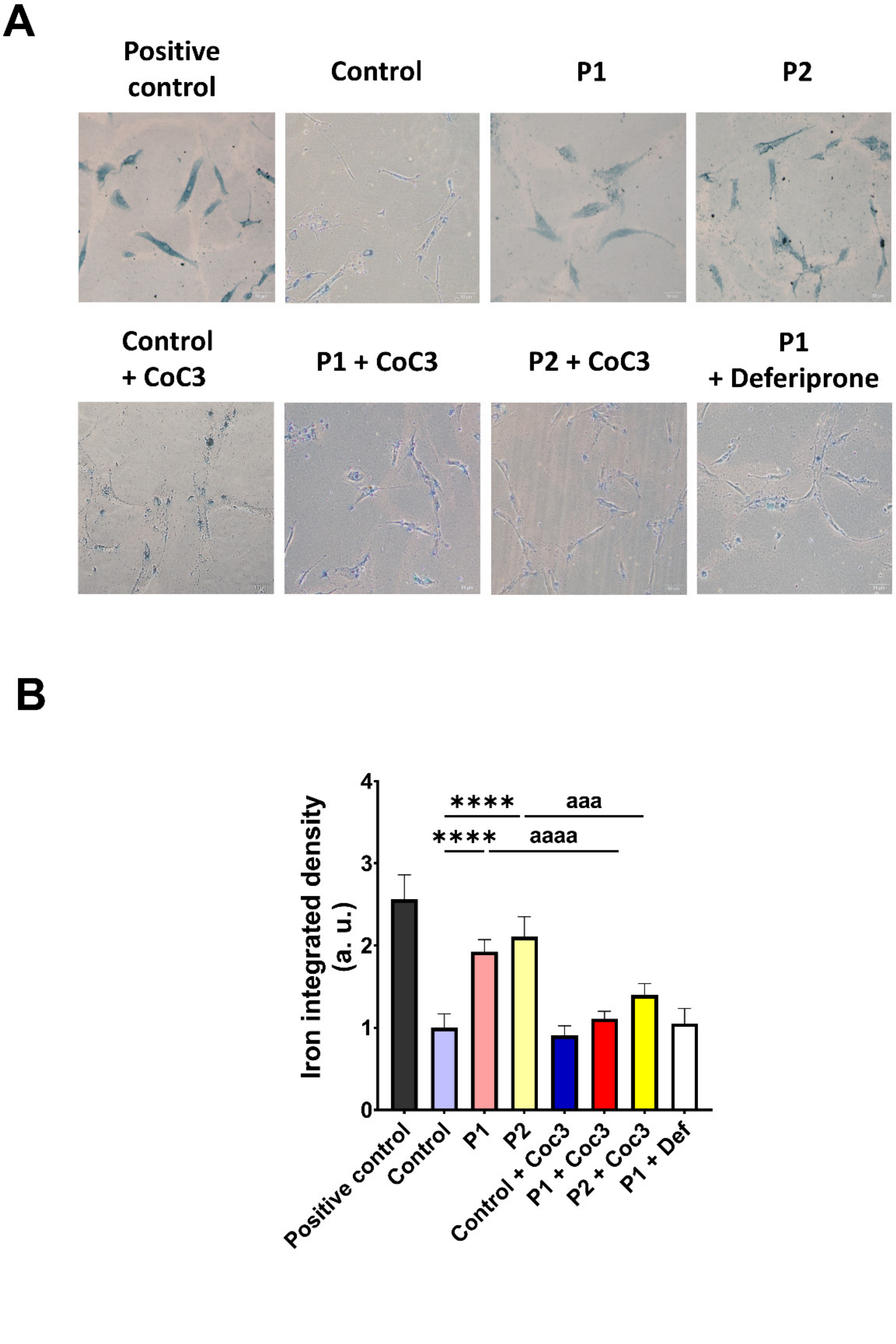

Figure S20. **Effect of CoC3 treatment on iron accumulation in fibroblasts from two EE patients**. (**A**) Untreated and treated control and EE patient fibroblasts (P1 and P2) were stained with Prussian blue. Patient cells were treated with 100 μM Deferiprone as negative control. A PKAN (pantothenate kinase-associated neurodegeneration) cell line was used as a positive control of iron accumulation. Images were acquired by a Zeiss Axio Vert A1 microscope. Scale bar: 50 μm. (**B**) Quantification of Prussian blue staining-integrated density. Images were analyzed by the ImageJ software (at least 30 images were analyzed per each condition and experiment). Data were referred to control and represent the mean ± SD of 3 independent experiments. **** $p < 0.0001$ between control and patient fibroblasts. [aaa] $p < 0.001$ and [aaaa] $p < 0.0001$ between untreated and treated patient fibroblasts. a.u.: arbitrary units.
